# In situ enhancement of coral larval settlement using acoustic enrichment, engineered habitats and living materials

**DOI:** 10.1038/s42003-026-10932-3

**Published:** 2026-10-08

**Authors:** Aaron Thode, Natalie Levy, Océane Boulais, Samapti Kundu, Marnie Freckelton, Daniel Schar, Michael Hadfield, Jessica Reichert, Hendrikje Jorissen, Netanel Kramer, Joshua Levy, Kira Hughes, the R3D consortium, Daniel Schar, Jessica Reichert, Hendrikje Jorissen, Joshua Levy, Kira Hughes, Benjamin Jones, Sean Mahaffey, Aricia Argyris, Mark Aruda, Ian Robertson, Zhenhua Huang, Ayrton Medina-Rodriguez, Mert Gokdepe, Brady Halvorson, Jon Chase, Charlotte White, Cami Dillon, Kristian McDonald, Anna Mikkelsen, Josh Madin, Mollie Asbury, Nina Schiettekatte, Marion Chapeau, Robert Toonen, Christopher Suchocki, Van Wishingrad, Christopher Jury, Madeleine Hardt, Claire Lewis, Claire Bardin, Joshua Kualani, Crawford Drury, Josh Hancock, Carlo Caruso, Andrea Grottoli, Shannon Dixon, Josh Voss, Allison Klein, Sid Verma, Alejandro Alvaro, Richard Argall, Kevin Chun, William Hicks, Alex LeBon, John Yeh, Aaron Thode, Oceane Boulais, Daniel Wangpraseurt, Samapti Kundu, Natalie Levy, Lindsey Badder, Stefan Kolle, Joshua Madin, Daniel Wangpraseurt

**Affiliations:** 1 Marine Physical Laboratory, Scripps Institution of Oceanography, University of California San Diego, 9500 Gilman Dr, La Jolla, CA USA; 2 Coral Reef Ecophysiology and Engineering Laboratory, Scripps Institution of Oceanography, University of California San Diego, 9500 Gilman Dr, La Jolla, CA USA; 3 Kewalo Marine Laboratory, University of Hawai’i, 41 Ahui St., Honolulu, HI USA; 4 Hawai’i Institute of Marine Biology, University of Hawai’i at Mānoa, 46-007 Lilipuna Rd., Kāneohe, HI USA; 5 Applied Research Laboratory, University of Hawai’i, 2800 Woodlawn Dr., Suite 263, Honolulu, HI USA; 6 University of Hawai’i at Mānoa, Honolulu, HI USA; 7 Ohio State University, Columbus, OH USA; 8 Florida Atlantic University, Boca Raton, FL USA; 9 Makai Ocean Engineering, Waimanalo, HI USA

**Keywords:** Conservation biology, Restoration ecology, Biogeography

## Abstract

Worldwide reef ecosystem degradation is inspiring efforts to enhance in-situ coral larvae settlement onto degraded or artificial reefs. Here we simultaneously test three approaches for enhancing settlement over two weeks in Kāne’ohe Bay, O’ahu, Hawai’i. Nineteen artificial structures, mimicking natural reef mesoscale roughness, were deployed along two transect lines at distances between 1 and 42 m from an “acoustic enrichment” underwater speaker broadcasting healthy reef sound. Twelve additional cinder blocks supporting micro-habitat structures designed to attenuate flow were also deployed at various distances. Some micro-habitats were coated with a living bacteria reef ink (Brink) to further induce larval settlement. Statistical regressions identify Brink presence, structure type, and speaker distance to 20 m as significant factors in predicting coral larval settlement density, but conclusions about acoustic enrichment efficacy are tentative. The cumulative effects of acoustic enrichment, living microbial materials, and custom-designed habitats on enhancing in-situ larval settlement justify further scaled-up reef restoration trials.

## Introduction

Coral reef ecosystems face increasing pressure from both local and global stressors. Pollution, urban development, and coastal modification have led to habitat degradation, while climate change-induced ocean warming and acidification further threaten coral survival^1,2^. The accelerating decline of coral reefs has raised global concern, given their crucial role in buffering coastlines from erosion, storm damage, and sea-level rise. They also provide critical ecosystem services, including coastal protection, biodiversity support, and fisheries production^3,4^. In response to this decline, significant efforts have been directed toward reef conservation and restoration, ranging from passive approaches that reduce environmental stressors, to active interventions that enhance coral recruitment and survival^3,5–8^. Examples of active approaches include natural reef restoration and even the construction of entire artificial structures to facilitate coral settlement and growth^3,9–14^. Advances in understanding the mechanisms that promote coral settlement and early-stage survival can improve both natural and assisted restoration efforts^15^. To address this challenge, researchers have explored various approaches to assist in coral settlement and recruitment^15–17^. These include custom-designed settlement surfaces with enhanced surface area and crevices, synthetic biofilms that release chemical attractants, and acoustic enrichment (AE), the process of broadcasting sounds recorded at healthy reefs to stimulate settlement behavior in both fish larvae and coral larvae at degraded or artificial reef sites. The Supplementary background provides further details and previous literature on these three approaches.

In this paper, we analyze a two-week July 2024 in situ field experiment in Kāne’ohe Bay, O’ahu, Hawai’i, during a natural spawning event of a native Hawaiian stony rice coral, *Montipora capitata*. This deployment was part of an effort by the Rapid Resilient Reefs for Coastal Defense (R3D) consortium to build an artificial coral reef off the windward shore of O’ahu. The experiment integrated the three approaches for enhancing coral larval settlement reviewed in the Supplementary background: acoustic enrichment (AE), custom-designed artificial structures, and living microbial materials applied to a subset of these structures. The experiment also deployed additional structures and autonomous long-term cameras to measure how mature fish and fish larvae presence were affected by AE^18^, making this deployment among the first to measure potential AE effects on both fish and coral simultaneously. The fish observations are important because fish activities, such as algal and coral grazing, can impact coral settlement, growth, and recovery^19,20^.

The field experiment took place over a sandy bottom in 4.5 m depth water. We deployed 37 artificial structures at distances up to 42 m from an underwater acoustic transducer broadcasting healthy reef sounds. Nineteen structures were “coral settlement modules” (CSMs), 3D printed clay structures that incorporate crevices of varying depths to increase the cryptic surface area of the structure^21^. An additional six structures consisted of cinder blocks with cm-scale volcano-shaped microhabitats glued to them (“Microhabitat cinder blocks,” or MCB-), designed to disrupt and slow current flow over the surface. Another six MCB structures were coated with “bio-reef ink” (Brink), a settlement-inducing synthetic microbial^22^ coating (“Microhabitat cinder blocks with Brink,” or MCB+). The final six structures were fish habitat modules (FHMs), designed to provide shelter for fish and fish larvae while also documenting their presence with autonomous cameras^18^.

**Fig. 1:**
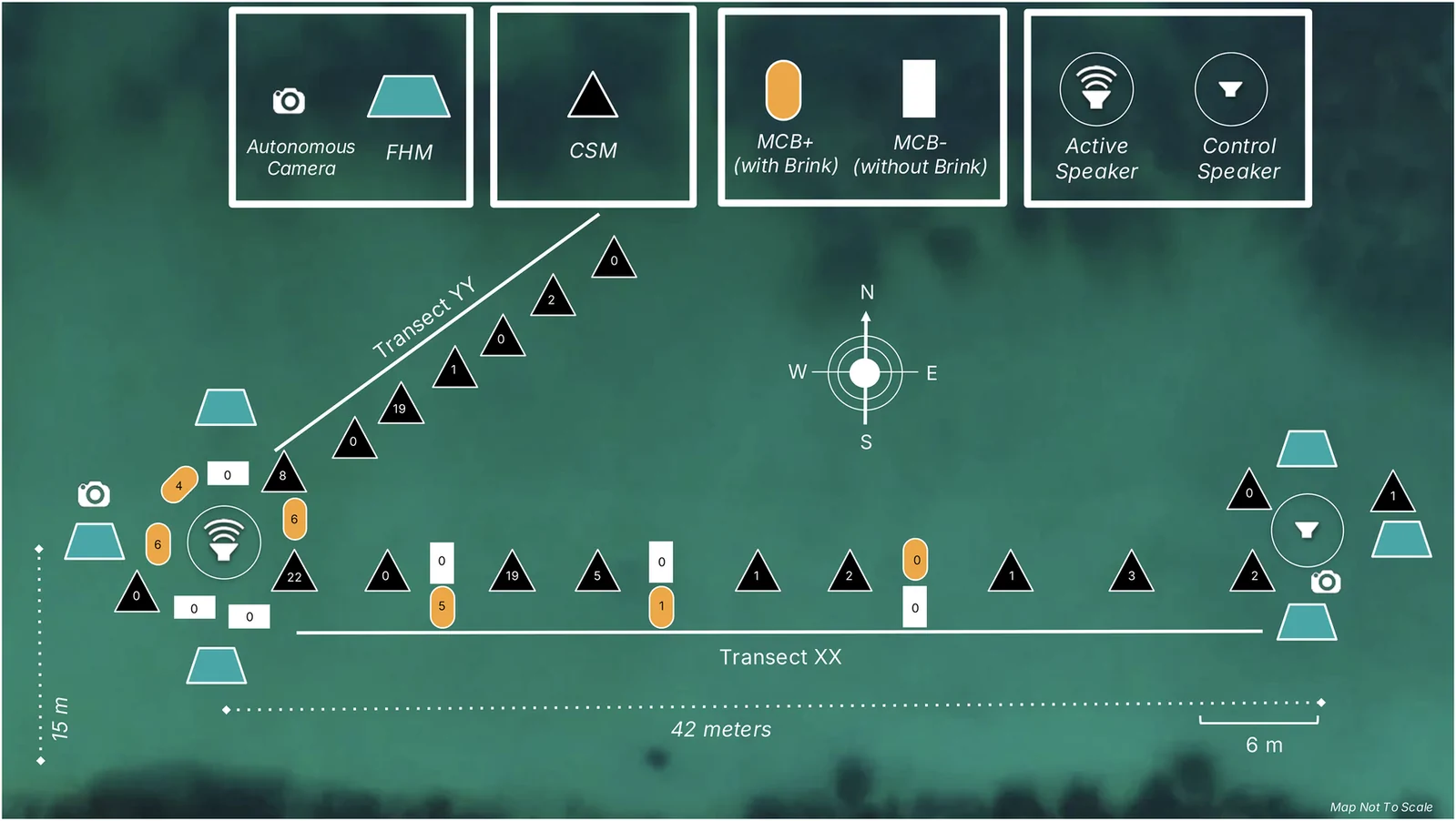
Experimental layout and raw coral larvae settlement counts. Symbol shapes and colors indicate different structure types. Numbers within symbols represent settlement counts on that structure on July 18th, 14 days after the new moon. Black triangles represent 19 coral settlement modules (CSMs), white rectangles represent six microhabitat cinder blocks (MCB-), orange ovals represent six additional MCB structures with “Brink” synthetic biofilm (MCB+), and teal trapezoids represent six fish habitat modules (FHM). See Tables 1 and 2 for precise distances of individual structures to the active playback speaker. The control speaker broadcast no sound to investigate whether acoustic playbacks attract fish. The map is not to scale. Background photograph provides an indication of the location of natural coral reefs surrounding the sandy bottom.

Figure 1 maps the location of all structures relative to the active speaker. The CSMs were deployed along two radial lines, or transects, from the active speaker, to test whether coral settlement counts decreased as distance increased relative to the speaker. Both the MCB+ and MCB- structures were also deployed side-by-side at various distances along one transect, permitting potential Brink effects to be statistically discerned from potential acoustic effects.

The total area covered by the experiment was nearly 700 m^2^, permitting the settlement structures to experience wide variations in acoustic playback level, while also accounting for uncontrolled variations in larval supply and potential fluctuations in chemical or other cues produced by the surrounding natural coral reef. One of the transects ran parallel to a fringing reef to address this latter uncontrolled factor. Over half the structures were placed in locations where no acoustic playback signal was detectable, permitting us to estimate background larval settlement rates in the experimental area.

Potential variation in fish presence along the transects was a potential confounding factor in interpreting changes in coral larvae settlement along the transects, and so the FHMs were deployed to document this potential effect. The FHMs were not deployed along a linear transect; instead, three structures were placed in a circle of 2-m radius around the active speaker, and another three structures were placed in a similar circle around a dummy speaker deployed 42 m away from the active one, at the end of one transect. An autonomous camera was mounted near both speakers to document whether fish presence differed at each end of the transect. While the species of mature fish could be identified by camera, fish larvae cannot be reliably identified to species from camera images, because many species’ larvae are morphologically very similar and often share overlapping traits. Thus, species-level larval fish ID typically requires physical collection, genetic analysis, and/or detailed microscopic examination^23^. Full details on the fish aspect of the experiment are provided in ref. ^18^.

Ambient acoustic recordings collected from a healthy reef 300 m from the site were broadcast nightly from the speaker, with the broadcast schedule centered around the new moon. Settlement count surveys were conducted via SCUBA operations 7 and 14 days after the new moon spawning of the coral species *Montipora capitata*. Fish presence at both the treatment and control speakers were documented via manual reviews of burst images collected from the autonomous cameras. Furthermore, on August 20, roughly one month after the speaker transmissions ended, photographs were taken of the MCB and CSM structures to document relative algal coverage, to determine whether the high concentration of structures around the active speaker were associated with increased fish grazing activity that could impact coral larvae settlement.

## Results

### Raw plots and tables of settlement density

**Fig. 2:**
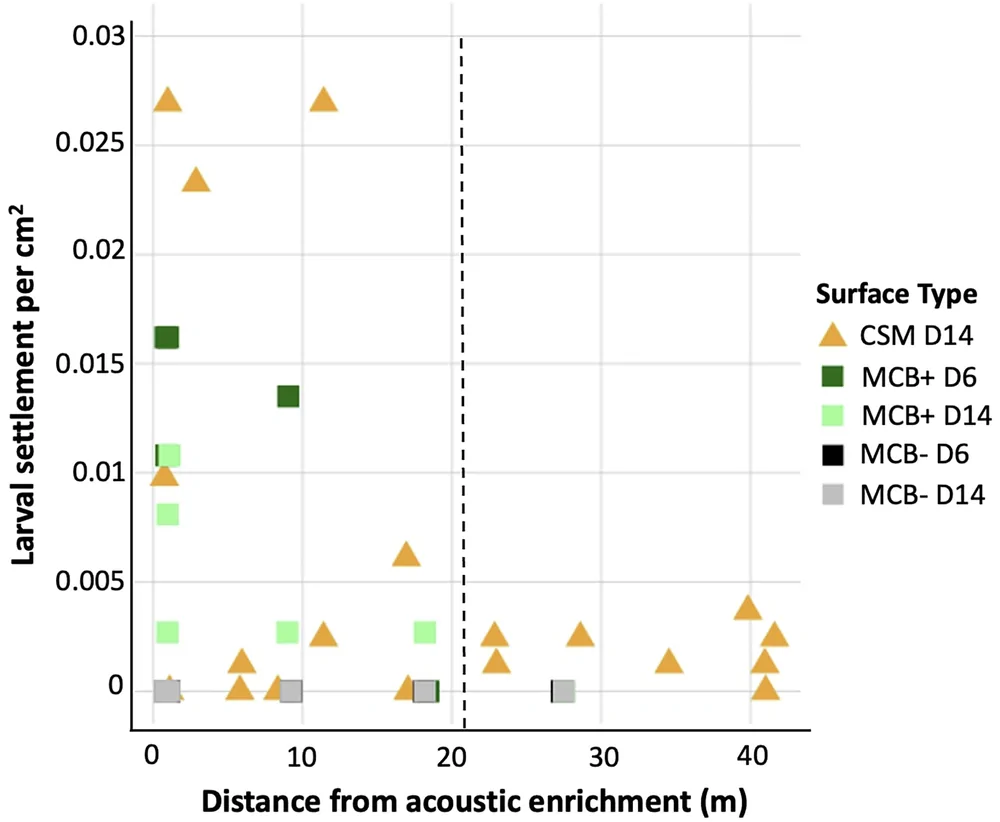
Relationship between coral larvae settlement density, playback speaker distance, and settlement surface type. Symbols represent combinations of survey dates (“D6” = July 11th; “D14” = July 18th) and three different surface types [coral settlement modules (CSM), and microhabitat cinder blocks with (MCB+) and without (MCB−) brink coatings]. The vertical dashed line indicates the modeled maximum distance (21 m) at which the active speaker playback becomes indistinguishable from the surrounding ambient coral reef sound.

Scatter plots of the raw settlement count data indicate that acoustic enrichment and microbial treatments are associated with higher observed settlements of coral larvae. Figure 2 plots coral settlement density vs. distance from the acoustic playback speaker, with symbol color and shape indicating a particular combination of surface type and observation date. The vertical dashed line at 21 m represents the distance at which acoustic modeling predicts that the AE playback levels fall below ambient sound levels generated by nearby coral reefs. Figure 2 suggests that coral larvae settlement counts beyond the 21 m speaker distance have a baseline settlement density of 0.0025 larvae per square centimeter. Higher densities are observed closer to the playback speaker, with densities greater than 0.005 counts/cm^2^ visible up to 18 m range from the speaker. A more quantitative analysis of this possible distance effect is provided in the next section.

Table 1 lists raw data for the MCB+ and MCB- structures on July 11th and July 18th, one and 2 weeks after the new moon spawning. The data include raw settlement counts, converted settlement densities, and distance from the speaker. The last two columns describe algal cover estimated from in situ photos, as described and discussed in the Supplementary Materials. Table 2 lists corresponding results for the CSM deployments on July 18th. (No CSM surveys were conducted on July 11 due to insufficient SCUBA support). The algal coverage for CSM structures was determined using methods described in the Supplementary Materials. Figure 1 also displays the raw counts on all structures on July 18th. No counts were noted on any of the FHM structures. Table 1 shows that while coral larvae settled on the MCB+ structures, no settlement was detected on the MCB- structures. The highest densities for MCB+ structures were observed on July 11th, with densities dropping 30–60% lower the following week. The settlement density on CSM structures are roughly twice as high as those observed on MCB structures, when observed on the same date (July 18).

**Table 1:** Settlement results on MCB surfaces measured one and two weeks after the new moon, expressed in terms of both raw counts and settlement density

Block	July 11	July 18	July 11	July 18	Distance	Algae July 18	Algae August 20
	Raw counts	Raw counts	counts/m^2^	counts/m^2^	(m)	Mean fraction	Mean fraction
a.1	0	0	0.0	0.0	27.4	0.31	1.00
b.1	0	1	0.0	27.0	18.3	0.85	1.00
c.1	5	1	135.0	27.0	9.1	0.93	1.00
d.1	6	3	162.0	81.0	0.9	0.79	0.98
e.1	4	1	108.0	27.0	0.9	0.76	1.00
f.1	6	4	162.0	108.0	0.9	0.75	0.99
a.0	0	0	0.0	0.0	27.4	0.95	1.00
b.0	0	0	0.0	0.0	18.3	0.88	1.00
c.0	0	0	0.0	0.0	9.1	0.98	1.00
d.0	0	0	0.0	0.0	0.9	0.94	1.00
e.0	0	0	0.0	0.0	0.9	0.96	1.00
f.0	0	0	0.0	0.0	0.9	0.94	0.99

The distance column shows the distance of a given MCB from the playback speaker in meters, and the last two columns indicate the fraction of the MCB surface covered by algae that was not crustose coralline algae (CCA). Surfaces that share the name letter label are equidistant from the playback speaker. A “0” label suffix indicates an MCB− structure and a “1” suffix indicates an MCB+ (Brink treated) structure.

**Table 2:** Settlement results on CSM surfaces measured on July 18th, expressed in terms of raw counts and settlement density

CSM	Transect	July 18	July 18	Distance	Algae August 20
		Raw counts	counts/m^2^	(m)	Mean fraction
A	XX	2	24.5	41.8	0.92
B	XX	1	12.2	40.8	0.98
C	XX	0	0.0	40.8	0.95
D	XX	3	36.7	39.9	N/A
E	XX	1	12.2	34.4	0.93
F	XX	2	24.5	28.7	0.97
G	XX	1	12.2	22.9	0.97
H	XX	5	61.2	17.1	N/A
I	XX	19	232.6	11.6	1.00
J	XX	0	0.0	5.8	0.92
K	XX	22	269.4	0.9	0.95
L	XX	0	0.0	0.9	0.15
M	YY	8	98.0	0.9	0.60
N	YY	19	232.6	3.0	0.93
O	YY	1	12.2	5.8	1.00
P	YY	0	0.0	8.2	0.95
Q	YY	2	24.5	11.6	0.87
R	YY	0	0.0	17.1	0.90
S	YY	2	24.5	22.9	0.63

The distance column shows the distance of CSMs from the playback speaker, and the last column indicates the fraction of CSMs covered by algae during an August 20th survey.

No significant difference in mature fish presence was detected between the active and control speaker; however, higher numbers of fish larvae were documented at the active speaker for a few days before and after the new moon during the July 2024 deployment^18^.

### Statistical regression of effects of acoustic enrichment, surface type, and living material on settlement density

Generalized linear model regressions assuming a zero-inflated Poisson (ZIP) process were fit to the raw settlement counts, with different coefficients evaluating the impacts of speaker distance, structure type, and Brink presence. Separate regressions were conducted for the CSM and MCB structures; both incorporated range (meters) as a continuous independent variable, but the CSM regression included transect line **TRANSECT** as an additional categorical variable, while the MCB regression included Brink presence **BRINK** as a categorical variable.

**Fig. 3:**
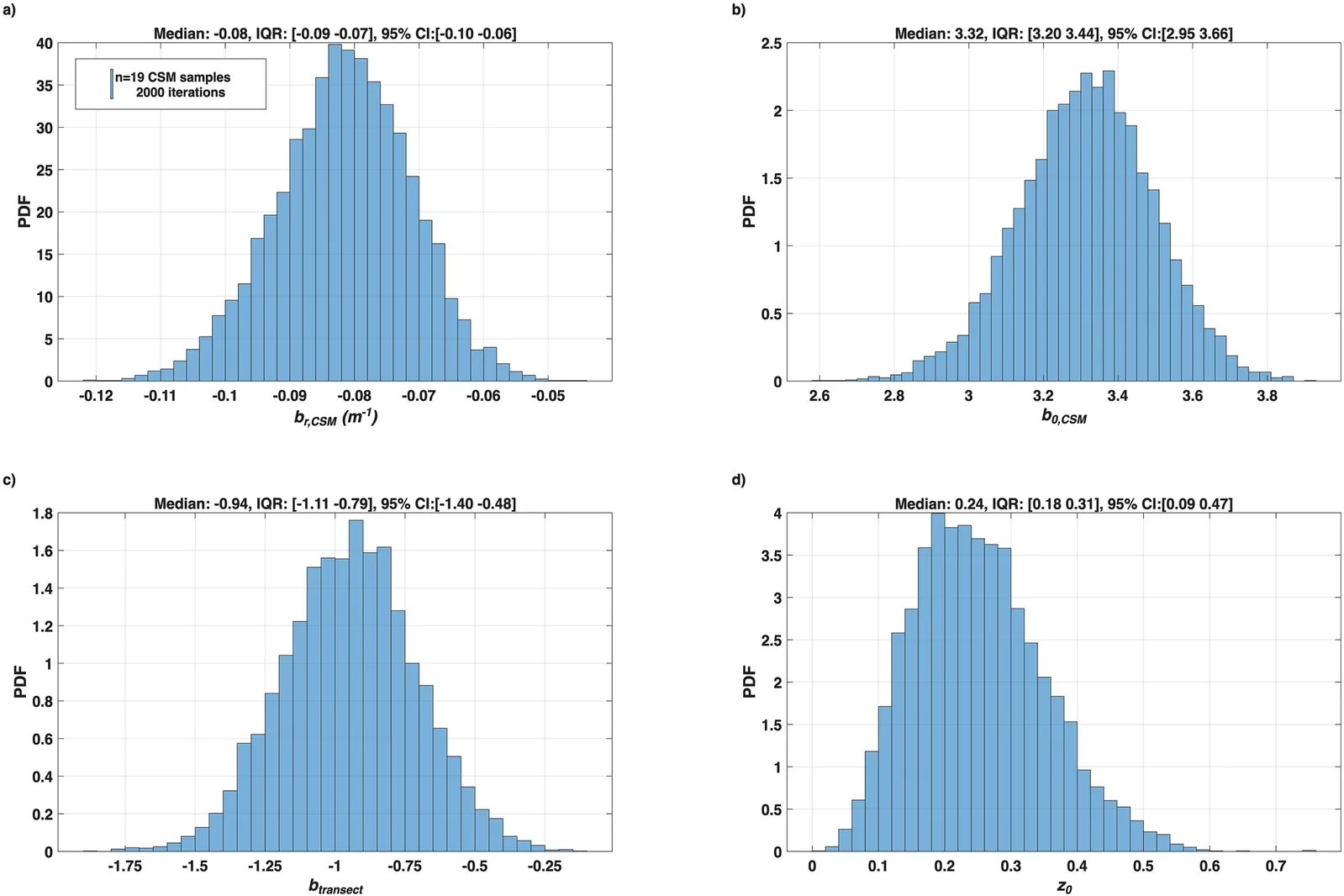
Estimated posterior probability density functions (PDF) for regression parameters for CSM surfaces (Eq. (1)). Median, interquartile range (IQR), and 95% credible intervals (CI) are also listed for each distribution. **a** *b*_*r*,CSM_, regression coefficient for distance from speaker; **b** *b*_0,CSM_, the intercept parameter; **c** *b*_transect_, coefficient for categorical variable **TRANSECT** when value is **YY**; **d** *z*_0_, parameter for zero-inflated Poisson model.

**Fig. 4:**
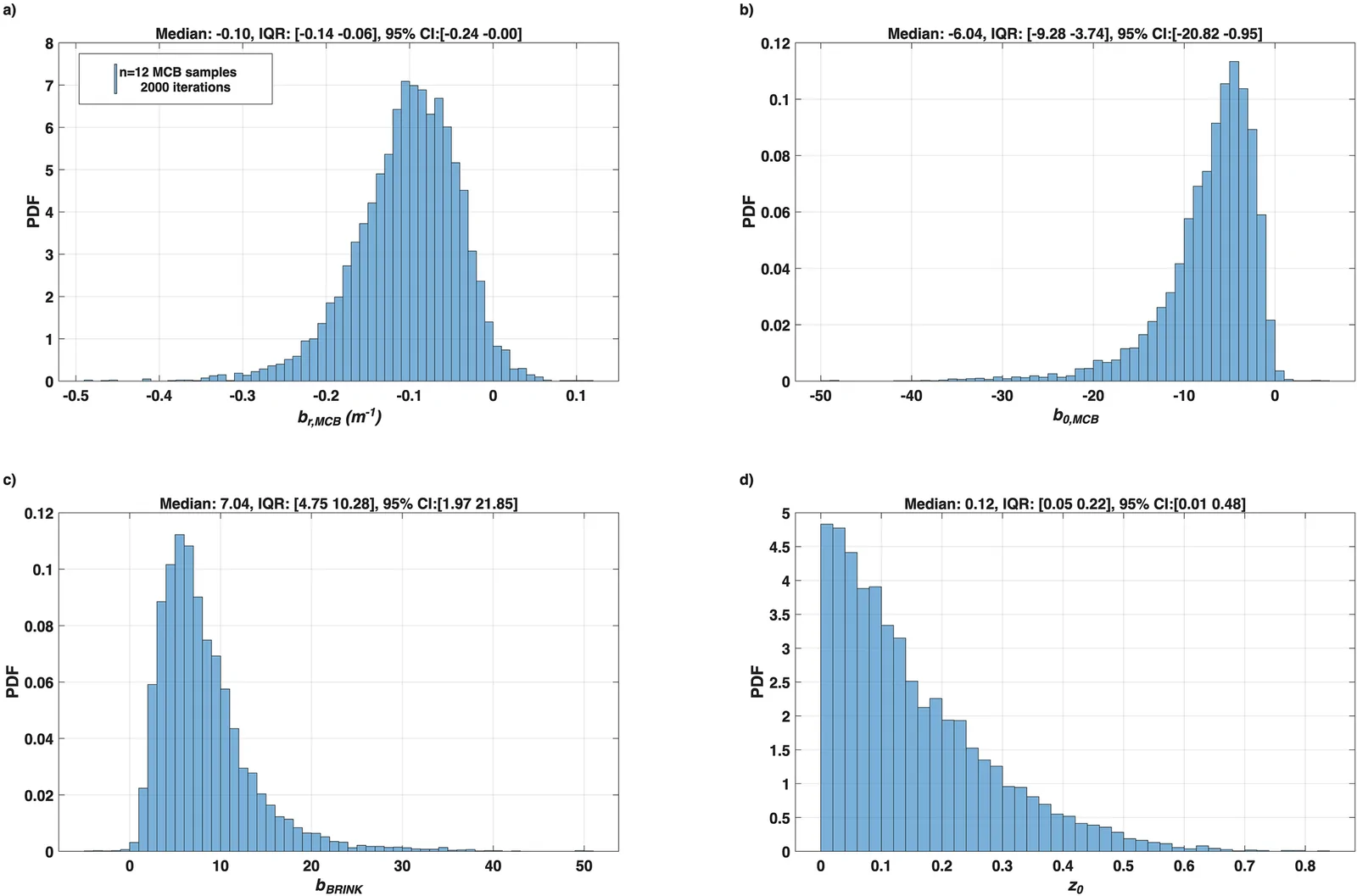
Estimated posterior probability density functions (PDF) for regression parameters for MCB surfaces (Eq. (2)). Median, interquartile range (IQR), and 95% credible intervals (CI) are also displayed for each distribution. **a** *b*_*r*,*MCB*_, regression coefficient for distance from speaker; **b** *b*_0,*MCB*_, the intercept parameter; **c** *b*_*BRINK*_, coefficient for categorical variable **BRINK** when value is **TRUE**; **d** *z*_0_, parameter for zero-inflated Poisson model.

Figures 3 and 4 show histograms of the estimated posterior distributions of all coefficients of the ZIP regressions. Note that ZIP regressions model the logarithm of the response variable, so the multiplicative impact of any effect is determined by increasing the coefficient to the power of *e*. No cross-terms between any independent variables were found to be significant. The 95% credible intervals for all regression coefficients exclude zero, demonstrating a statistically strong effect of speaker distance and **BRINK** on larval settlement density. Both independent CSM and MCB regressions yield similar regression coefficients for distance, with median values *b*_*r*,CSM_ = −0.08 and *b*_*r*,MH_ = −0.10. However, the CSM regression 95% credible interval is much tighter [−0.10, −0.06], due to its larger sample size. The MCB structures provided only six effective samples, yielding a wider credible interval, including a 95% upper limit that is just below zero. The two surfaces diverge in estimates of the underlying *z*_0_ parameter for the zero-inflated Poisson process, with median values of 0.24 (CSM) and 0.12 (MCB).

**Fig. 5:**
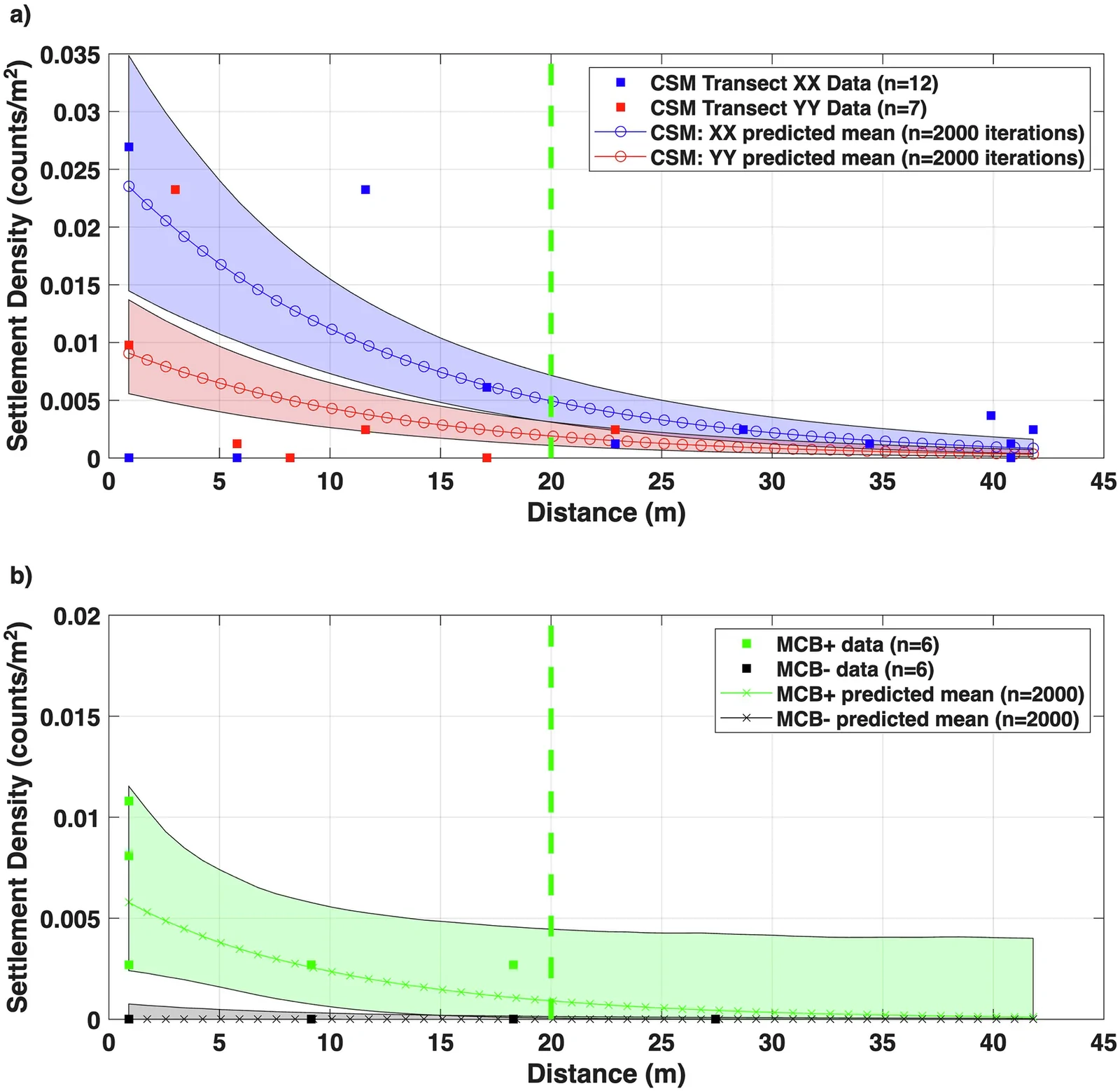
Predicted settlement density vs. speaker distance and three settlement surfaces. **a** Predicted CSM settlement density along both transects; **b** predicted surface density for MCB+ and MCB− surfaces. Shaded areas indicate the 95% confidence interval for the expected prediction value. The vertical dashed line indicates the estimated range at which playback sounds merge into the ambient sound (21 m).

Figure 5 uses the regression models to predict coral density as a function of range for the CSM, MCB−, and MCB+ surfaces, along with the 95% prediction confidence levels of the mean. Because the link function for a Poisson regression uses a natural logarithm, the negative coefficient for range creates an exponential decrease in mean settlement density with range, with mean density halving with every 7 m increment of range (Fig. 5).

Unsurprisingly, given the lack of settlers on the MCB− structures, the MCB regression analysis produces large values for the **BRINK** coefficient *b*_BRINK_, with posterior estimates yielding a median value of 7.04 and 95% credible interval between 1.97 and 21.85. The exact magnitude of the coefficient has little meaning, given that no settlement was detected on the MCB− structure, but the implied scale of the results (i.e., a factor of *e*^7.04^ = 1100 increase in density) indicates the strength of the **BRINK** effect.

The CSM regression also found that settlement rates along Transect XX were roughly 2.5 [*e*^0.94^] times greater relative to settlement rates along Transect YY (Figs. 3c and 5a).

The regression results provide clear evidence that synthetic microbial coatings enhance coral larvae recruitment, but interpreting the distance effect as a response to AE is not straightforward. Alternative explanations for the distance effect include a random hotspot of settlement coincidentally near the active speaker, and more successful larval recruitment on structures with less algal coverage arising from increased fish herbivory near the active speaker. The Discussion examines evidence for these alternative interpretations.

### Fish presence results

Examples of frequent fish species sighted at both sites include the Ringtail surgeonfish (*Acanthurus blochii*), yellowfin surgeonfish (*Acanthurus xanthopterus*), Hawaiian trevally (*Caranx melampygus*), Hawaiian goby (*Sicyopterus stimpsoni*), and their symbiotic partner, the Hawaiian snapping or pistol shrimp (*Alpheus* spp.). Also sighted were conspicuous sea cucumbers (*Stichopus conspicuous*) and green sea turtles (*Chelonia mydas*).

No significant difference in mature fish presence was detected between the active and inactive speaker sites, using either a non-parametric Wilcoxon rank-sum test (WRST) or a parametric ZIP regression. Table 3 shows that *p* values for the WRST were 0.18 and 0.49 for two metrics of fish presence: mean fish counts per camera and MaxN, the maximum number of fish visible in a single image. The ratio of the estimated mean values of the MaxN metric (AE multiplier) at the active/control speakers was only 0.7, and the regression coefficient *b*_MF,treatment_ associated with **TREATMENT** (Eq. (3)) has a 95% confidence interval [−1.21, 0.42] that encompasses zero. Two separate deployments that focused entirely on fish response (June 2024 and August 2023) also yielded no significant differences in mature reef fish counts^18^.

**Table 3:** Summary of statistical tests for mature fish during July 2024

Month Year	Wilcoxon RS test (*p* value)		ZIP regression (Estimate [95% CI])		
	Counts/Camera	MaxN	*b*_treament_	*b*_MoonPhase_	AE multiplier
July 2024	0.18	0.49	−0.38 [−1.21 0.42]	**−1.88** [−3.51 −0.46]	0.7

Results in **bold** indicate statistically significant (*p* <;0.05) differences between treatment and control sites for the rank-sum test. The AE multiplier, ${e}^{{b}_{{{\mathrm{treatment}}}}}$ is the estimated ratio of MaxN between treatment and control sites.

Significant differences in fish larvae counts were detected between the active and control speakers around the new moon. However, the absolute numbers were small during this July deployment: roughly 25 larvae versus 2 at the active and control speaker sites. Fish larvae near both speakers also disappeared within a few days of the new moon. See ref. ^18^ for further details on the analysis of AE impacts on fish larvae presence for both this deployment (July 2024) and two other deployments (August 2023, June 2024) at the same location. We therefore conclude that differences in fish presence along a transect are a less likely explanation for the coral larvae settlement distance effect presented in the previous subsection.

## Discussion

Although this full experiment was conducted just once, the existence of multiple settlement structures and microbial/control surfaces permitted statistically significant conclusions to be drawn about the relative efficacy of the Brink synthetic biofilms and different settlement surfaces. For example, Figs. 4c and 5b show that the application of Brink to MCB structures was associated with higher coral larvae settlement, because no settlement at all occurred on the MCB− control blocks. The settlement density was also clearly higher on CSM structures vs MCB+ structures, indicating that crevices were more relevant for settlement than boundary flow disruption in this low-current environment. Combining volcanic microhabitat structures with the helical dome design may capture the advantages of both designs; although this combination was not tested here, upcoming designs incorporate both approaches.

The independent regression analyses for both the CSM and MCB surfaces also found a significant and similar relationship between settlement counts and distance. This experiment monitored settlement over a much larger area (≈700 m^2^) compared with previous published field efforts, which helped compensate for uncontrolled factors like larval surface density and potential variations in chemical and natural acoustic cues across the site. For example, 16 structures were far enough from the speaker such that they experienced no acoustic enrichment effect; therefore, we were able to estimate background settlement rates that showed little variance or spatial variation.

To check whether the CSM regressions were dominated by structures within two meters from the speaker, we conducted a sensitivity analysis by removing various CSM samples and then recomputing the regression coefficients. Table 4 lists the various combinations tested, along with the resulting summary statistics for the distribution for *b*_*r*,CSM_ (the range coefficient plotted in Fig. 3a). It was found that five data samples (K,L,M,N,I) had to be removed before the distance effect became insignificant (i.e., 95% CI spans zero). Even if all CSM samples at distances less than 4 m from the speaker are removed, the resulting regression coefficient distribution remains similar due to the counts at CSM I (11.6 m range) and H (17.1 m range). We conclude that the smooth, model-supported distance response curve shown in Fig. 5 is not just an artifact resulting from high settlement counts immediately adjacent to the speaker and low background counts everywhere else.

**Table 4:** Sensitivity of the CSM distance coefficient *b*_*r*,CSM_ to various data points in Table 2

Description	Samples	Estimate	Error	Lower 95% CI	Upper 95% CI
All samples	19	−0.08	0.01	−0.10	−0.06
Remove all samples ≤2 m range	16	−0.11	0.02	−0.15	−0.07
Remove all samples ≤3 m range	15	−0.09	0.02	−0.13	−0.05
Remove CSM I only	18	−0.08	0.01	−0.11	−0.06
Remove all samples >7 and ≤3 m removed	14	−0.02	0.03	−0.07	0.04

The CSM settlement results were also influenced by transect, with the **XX** transect associated with higher settlement. This result may simply be an artifact of how the **K**,** L**, and **M** CSMs next to the speaker were arbitrarily assigned to different transects. A more intriguing possibility is that the **XX** transect was parallel to a reef wall, and thus may have been more exposed to possible chemical or acoustic natural reef cues than the **YY** transect. Unfortunately, the spatial extent of the sandy patch used by this particular experimental setup was too small to permit a second transect to be placed in the opposite direction of the **XX** transect, also parallel to the reef wall. Now that the effective range of the distance effect has been established to be around 17–20 m, a future experiment may be able to accommodate multiple transect designs.

The speaker distance effect seems both statistically significant and robust, and the acoustic measurements and propagation models are consistent with an interpretation that the observed settlement arises over distances where the playback signal levels exceed existing background sound levels. Despite this, we cannot definitively conclude that acoustic enrichment is responsible for this distance effect. At least two possible alternative explanations exist besides AE for the observed range effect. Below, we list each explanation and discuss the evidence for and against each one.

- **Random settlement:** Because only one experiment was conducted, it’s possible that the observed effect arose from a coral settlement “hotspot” randomly occurring near the same location as the active speaker. However, the experimental design sampled a relatively large area (≈700 m^2^) and found no evidence of other random hotspots in the area. For example, the area beyond the estimated influence of the active speaker (distances >21 m, or 527 m^2^) showed a nearly uniform range-independent diffuse level of settlement of about 0.0025 larvae/cm^2^ (Fig. 2). Additional experiments with the active source placed in different locations will further reduce the likelihood of this alternative explanation.
- **Fish herbivory:** Herbivorous fish are known to impact fleshy algal cover on reefs^24^. Therefore, if larger numbers of herbivorous fish are present near the active speaker than along the transect lines, then less algal cover may occur near the speaker, permitting higher levels of successful settlement and recruitment. The increased fish presence may be due to the presence of the active speaker, or because of the high concentration of MCB, CSM, and FHM structures around the active speaker (Fig. 1). We investigated this hypothesis two ways: by monitoring the active and control speakers with autonomous cameras (Methods: Fish presence analysis) and by measuring algal coverage across the site after all AE work had been completed (Supplementary Methods). As discussed previously, no statistically significant difference in mature fish presence at the active or control sites was observed during the July 2024 experiment^18^. Furthermore, two additional deployments of FHMs with the active speaker switched to a different location also found no difference in mature fish presence between the active speaker and control sites. However, the same analysis also found that AE was associated with higher numbers of fish larvae over the course of a few days surrounding the new moon. The additional photographic analysis of algal coverage on the CSM and MCB (Supplementary Fig. 1) found no statistically significant linear correlation between algal coverage and distance from the active speaker. The lack of observed difference in mature reef fish presence at the treatment and control site would seem to provide evidence against this alternate hypothesis. However, we cannot definitively rule out the possibility that increased fish *larvae* presence at the AE site might indirectly affect coral settlement, either through increased herbivory or even the generation of potential chemical cues by the fish larvae, that could then attract coral larvae. No specific chemical mechanism between fish presence and coral larvae response seems to exist in the literature, but future experiments in this vein might consider collecting water samples to test for possible differences in chemical cue levels.

We believe that a direct coral larvae behavioral response to acoustic enrichment is the most likely explanation for our observations, but further replicate experiments are clearly needed to eliminate the possibility of random “hotspot” settlements. Furthermore, additional experiments are also needed to better understand potential seasonal and physical oceanographic factors that may degrade responses to AE playback.

**Fig. 6:**
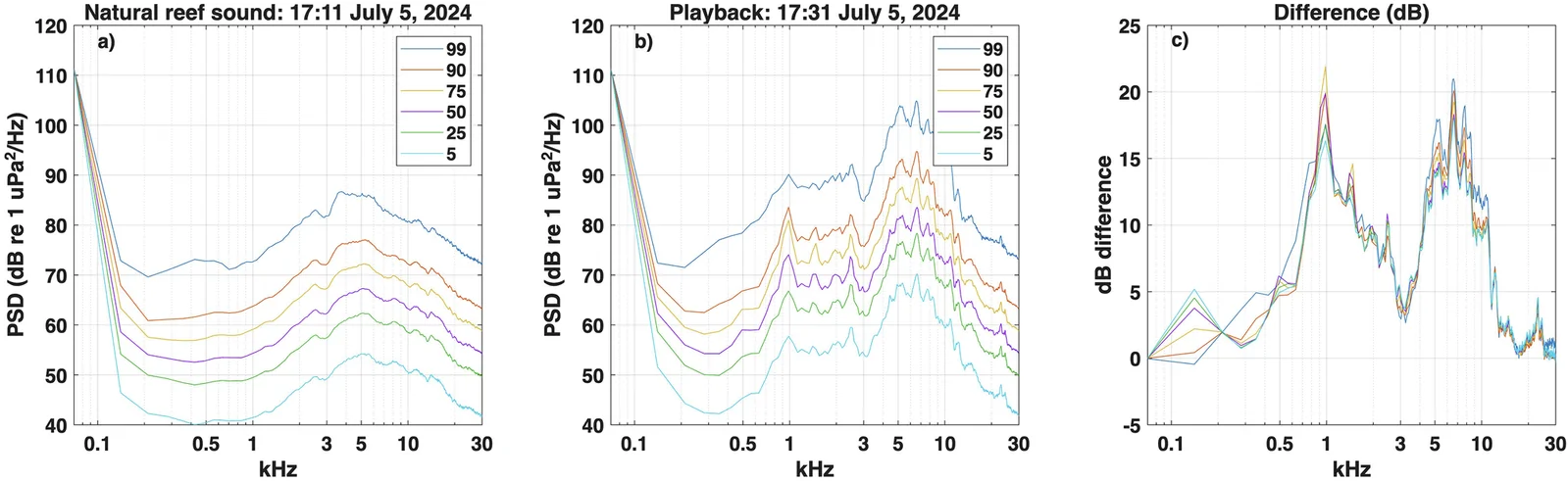
Measured power spectral density levels (dB re 1 μPa^2^/Hz) at 1 m range from the speaker. **a** Background level percentiles measured at 17:11 local time on July 5th, 2024 (new moon); **b** levels after playback began at 17:30; **c** difference in levels (signal-to-noise ratio) in dB.

Two additional aspects of the acoustic enrichment results are worth mentioning. First, the distance over which the AE effect is observed (21 m) is similar to the 30 m limit reported by AE work from ref. ^25^ in the US Virgin Islands, although Fig. 6 from that paper finds few differences in settlement behavior with distance from the playback speaker.

Second, both our results and the ref. ^25^ leaves open the question as to which frequency bands or signals from a coral reef environment are particularly relevant to coral larvae response, or whether the entire broadband field, including ultrasonic frequencies, is relevant. Both studies broadcast acoustic energy that exceeded background ambient levels across a couple of frequency bands: between roughly 500 Hz and 1 kHz, and roughly 3 to 15 kHz. In our particular study, we even broadcast detectable energy into the ultrasonic range (21 kHz) at 5–6 dB SNR (Fig. 6c).

Existing literature tends to focus on low-frequency (1 kHz and below) sounds as cues for both fish and coral larvae (e.g., ref. ^26^), based on analogies to other invertebrate responses and the results of one study^27^ that found higher levels of settlement behavior at a reef with higher ambient levels below 1 kHz.

The low-frequency content of reef sound may indeed be relevant to coral larvae behavior, but we are also intrigued by the relatively high levels of mid-frequency (3–15 kHz) and ultrasonic (20+ kHz) sound present in the natural ambient environment off Kaneohe Bay. Figure 6a shows that the ambient sound levels associated with 20–30 kHz equivalent to or higher than the levels associated with 500 Hz to 1 kHz. This energy arises from pulses from crustaceans similar to snapping shrimp. A review of the SoundTrap recordings sampled at 96 kHz finds that numerous pulses have peak intensities that exceed 40 kHz. Ultrasonic sound is relatively rare in most ambient marine environments, aside from marine mammal echolocation clicks, so the considerable ultrasonic energy present around coral reefs would seem to be a simple yet distinctive acoustic cue for reef presence. Propagation simulations indicate that over ranges of a few tens of meters, ultrasonic sounds experience relatively little attenuation compared to lower-frequency sounds. The reason for this is the relatively small spatial scales covered by the playbacks: higher in-water (Thorpe) attenuation associated with ultrasonic sounds only becomes significant over extended ranges of hundreds of meters, not tens of meters. We thus speculate that ultrasonic sound bands, along with low and mid-frequency bands, may be a distinctive identifying acoustic feature of coral reefs and are worth measuring and broadcasting in future AE experiments.

## Methods

### Study location

**Fig. 7:**
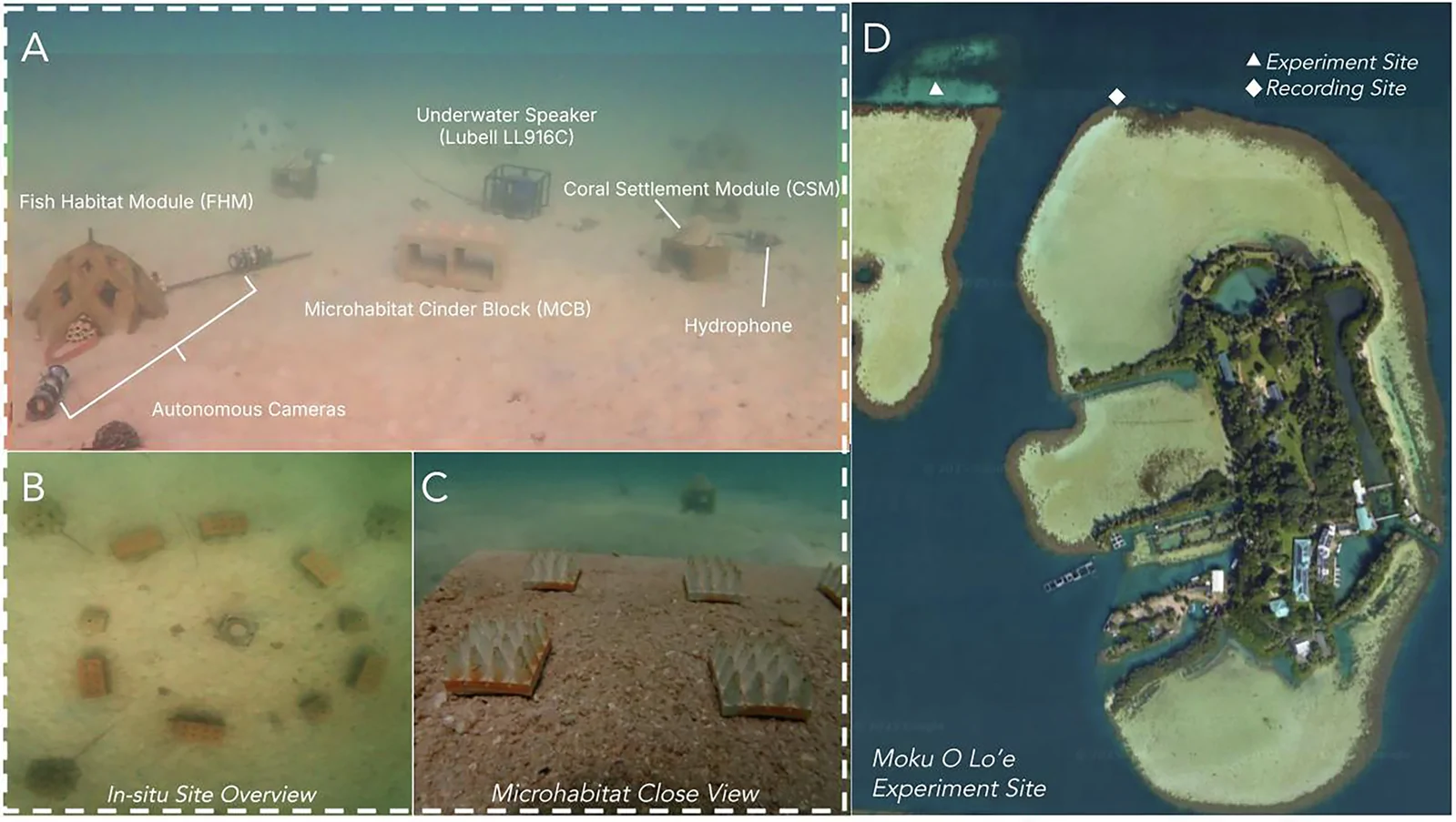
Photographs of the experimental site. **A** Experimental layout of the remote test site in 2024. Among the equipment visible are autonomous cameras, coral settlement modules (CSM), fish habitat modules (FHM), microhabitat cinder blocks (MCB), an autonomous SoundTrap ST300 hydrophone, and a Lubell LL916C speaker. **B** Aerial view of the playback site during 2024 deployments. The diameter of the “fairy ring” circle is 2 m. **C** Close up of microhabitat structures glued to an MCB. **D** Location of study sites in July 2024, at Reef 3 in Kāne-ohe Bay, O'ahu. The location where the playback files were recorded in August 2022 is also shown.

Field trials were conducted during July 2024 on a sandy seafloor in 4.5 m (mean tide level) depth water next to Reef 3, a patch reef on the northwestern side of Moku o Lo’e Island in Kāneohe Bay, Hawai’i $(2{1}^{\circ }2{6}^{{\prime} }1{6}^{{\prime\prime} }{{{\rm{N}}}}15{7}^{\circ }4{7}^{{\prime} }3{1}^{{\prime\prime} }{{{\rm{W}}}})$ in close vicinity of the Hawai’i Institute of Marine Biology (HIMB) (Fig. 7). The site, which encounters relatively little tourist vessel traffic, was surrounded by regions of natural coral reef, with one side of the sandy area bounded by straight fringing reef wall, and the other sides characterized by patchy reef. All speakers were at least 15 m away from any reef cropping.

### Acoustic enrichment equipment and recordings

#### Equipment

The design and implementation of an autonomous underwater acoustic playback system is centered around a programmable PIC-32 microcontroller, powered by 180 D-cell NiMH batteries to achieve an energy storage capacity of 3.8 kW-h, sufficient for three weeks of continuous nighttime operation without the need for a surface buoy. The batteries are divided between two pressure cases, each with a length of 81.3 cm and a diameter of 21.6 cm. A 10 m cable connects a Lubell LL916C underwater electrodynamic loudspeaker^28^(200 Hz–23 kHz [500 Hz–21,000 Hz ± 10 dB]) to the primary case, which contains the playback electronics. A second 10-m cable connects the primary case to the auxiliary battery pressure case. The long length of this second cable was chosen to permit each pressure case to be lowered independently from a boat without requiring connections to be made underwater. The power cable also contained a spare wire in case damage to the line occurred during a field deployment.

At the site, the gray pressure cases were placed side-by-side 10 m to the west of the active speaker to reduce the odds of the cases visually attracting animals. The speaker itself was mounted in a cage 15 m away from any nearby natural reef outcroppings, oriented vertically to broadcast sound towards the ocean surface, under the hypothesis that any coral larvae response would be predominantly vertical descent from the surface. The playback system features a magnetic switch which activates a 6-min playback test of natural reef noise, independent of any preset scheduling, permitting in situ testing of the playback functionality.

#### Recordings

Passive acoustic data used by the playback device were collected by a SoundTrap ST300 (Ocean Instruments, NZ; sensitivity 173.3 dB re 1 uPa/V) at $2{1}^{\circ }2{6}^{{\prime} }1{5}^{{\prime\prime} }{{{\rm{N}}}}15{7}^{\circ }4{7}^{{\prime} }2{5}^{{\prime\prime} }{{{\rm{W}}}}$ in Kāne‘ohe Bay during August 2022, a healthy reef location 300 m from the eventual test site (Fig. 7D). Snorkeling surveys by one of the co-authors (DS) confirmed the area has a high biodiversity of coral and fish species, relative to the typical habitats found within Kāne‘ohe Bay. The SoundTrap continuously recorded coral reef sound at a 96 kHz sampling rate for 8 days centered around the full moon phase, and an additional 8 days centered around the new moon phase. These data were downsampled to 36 kHz WAV files using a finite impulse response (FIR) lowpass filter, trimmed to begin at sunset and end at sunrise, and transferred to the 128 Gb flash memory on the playback equipment.

#### Playback schedule

The playback software had a unique “playlist” for each day of the month, listing the names of multiple WAV-formatted audio files recorded in 2022 that match the same lunar phase as the playback day. The software also had a master schedule specifying start and stop times for each day of the month. Acoustic playbacks were scheduled daily from roughly 17:30 to 07:00, beginning with a standard six-min reef noise file, followed by a looped playback of the playlist files. The standardized six-minute playback file facilitated confirmation that the playbacks activated successfully in the SoundTrap data. If a particular playback day had no corresponding recorded data from a corresponding lunar phase, a recording day with the closest match was used. An internal log file recorded the specific playlist file and the activation times. Playbacks were only conducted at night because *Montipora capitata* is a nocturnal broadcast spawner whose gamete release is synchronized to nights around the new moon in summer^29^.

Playbacks began the evening of June 28th, 2024, 1 week before the new moon, and ended July 20th, roughly 3 weeks after activation, 1 week after the last coral settlement survey.

#### Playback field measurements and propagation modeling

The propagation characteristics of the source were both measured and modeled.

Propagation loss from the speaker was initially measured on July 6th, 2023, during initial engineering trials at the site, using the same SoundTrap ST300 originally used to record the playback data. The recorder was placed 6.1, 12.2, 24.4, and 48.8 m away from the speaker. At each location, the playback system broadcast a distinctive song along with a set of tones between 500 Hz and 10 kHz, from which an effective propagation loss of 3 dB per doubling of range was estimated across all measured frequencies. The song and tones were distinctive enough to be readily identified in the acoustic data.

The playback source level was then adjusted such that the root mean square (RMS) received levels, integrated between 1 and 15 kHz, would match ambient RMS levels (signal-to-noise ratio of 0 dB) at an estimated 21 m range from the speaker. The dominant acoustic energy from the reef occurred over the 1–15 kHz band (Fig. 6a) due to impulsive crustacean sounds. However, the playback also included significant energy between 500 Hz and 1 kHz, a band typically associated with fish sounds and dubbed the “low-frequency range” in previous coral reef acoustic literature^25,26^.

**Fig. 8:**
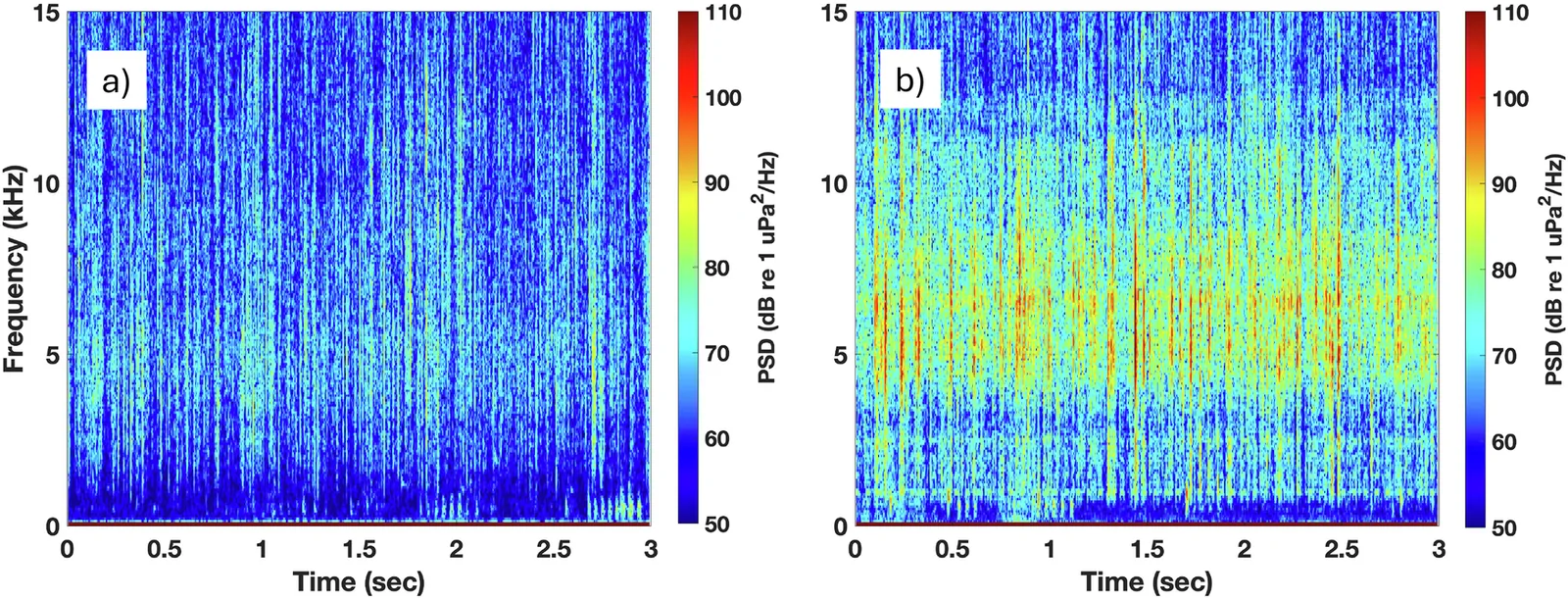
Example of a playback signal. Spectrogram of power spectral density (dB re 1 μPa^2^/Hz) for **a** background sound on July 5, 2024, 17:19 local time; and **b** AE sound, broadcasting at 17:30. Both figures use 2048 pt FFTs, 50 kHz sampling rate, 75% overlap. Data were recorded on a SoundTrap ST300 placed on the ocean floor 1 m away from the playback speaker.

During the full experiment in July 2024 the same SoundTrap was placed 1 m away from the speaker to independently verify playback levels. Figure 8a, b shows spectrograms of the sound field a few minutes before and after playback began on July 5th, the evening of the new moon. Figure 6 shows the measured percentile spectra of both the ambient (Subplot a) and playback sound expressed in terms of 1 m range from the speaker (Subplot b), with units expressed as power spectral density (PSD). The percentile curves span 30 dB, indicating the high impulsive content of both the ambient sound and playback data. Both the ambient and playback sounds are dominated by crustacean snaps over a frequency band between 2 and 15 kHz. Sounds below 1 kHz are 5–10 dB lower than the crustacean band. The high intensities visible below 100 Hz seem to arise from flow and other non-acoustic artifacts; manual reviews of fish sounds in the recordings find little energy below 100 Hz.

Figure 6c shows that the largest signal-to-noise ratios (SNR) of the playbacks, relative to ambient sound, are between 15 and 20 dB, occurring between 500 Hz and 1 kHz and between 5 and 10 kHz. Thus, frequency bands associated with both fish and crustaceans have similar playback SNRs relevant to ambient background sound. The frequency-dependent SNR characteristics of this output, including the high-SNR 3–10 kHz components, are similar to those detected during another AE experiment at the Virgin Island sites in ref. ^25^, Fig. 3, although the Hawaii site has higher levels of low-frequency reef noise, so the lowest frequency attaining a 5 dB SNR is 500 Hz, instead of 200 Hz at the Virgin Island site. Some ultrasonic output with up to 5 dB SNR occurs between 20 and 25 kHz, which may be electrical hum from the speaker, as the peak frequency in the recordings was 16 kHz.

**Fig. 9:**
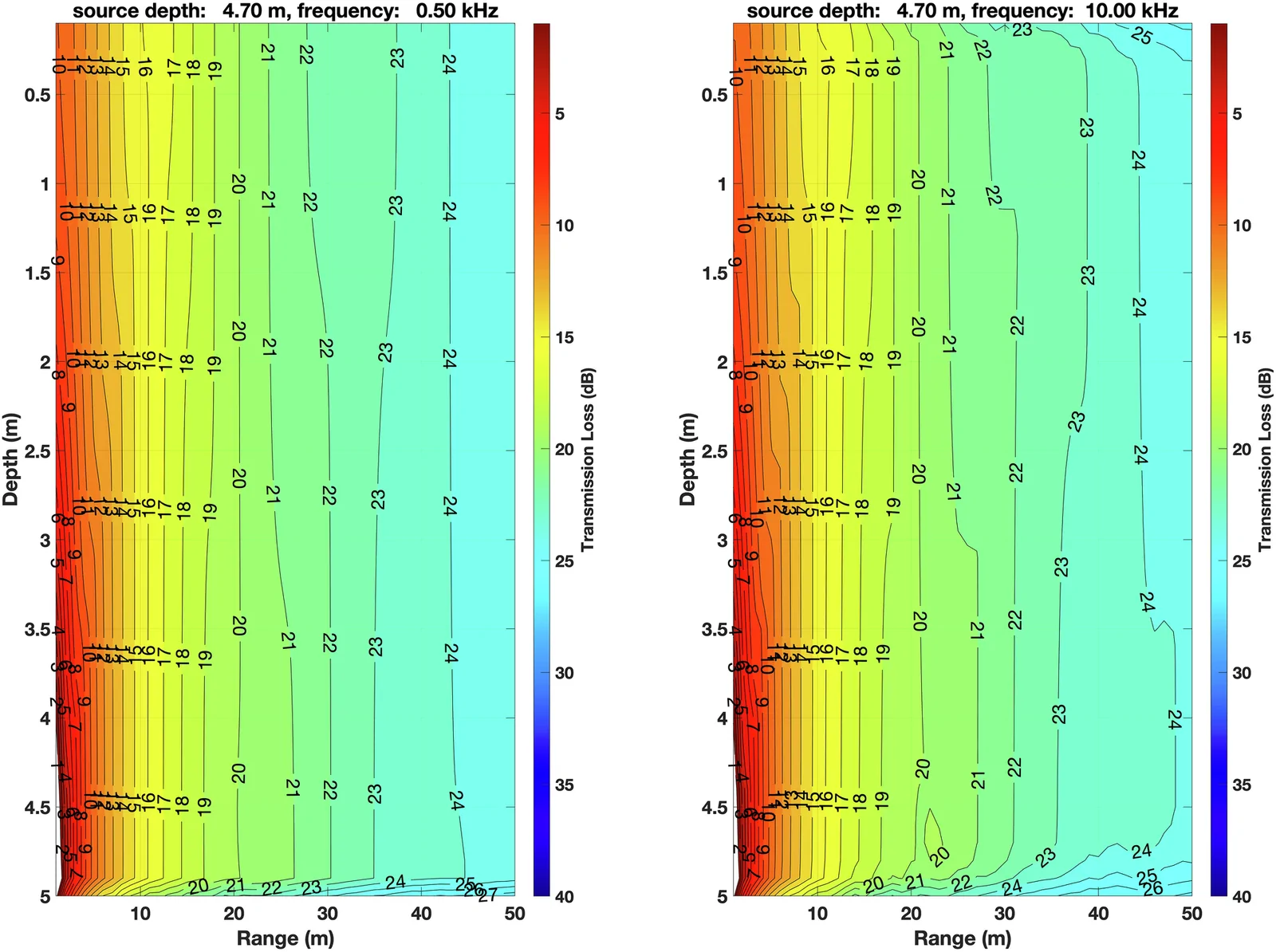
Modeled propagation loss (dB) from directional Lubell speaker. Contour plots of loss are shown for 500 Hz (left) and 10 kHz (right) frequency.

Acoustic modeling was used to confirm the propagation range of the acoustic playback vs frequency. The site’s flat bathymetry and sandy bottom significantly simplified acoustic modeling of propagation loss, using the incoherent version of the ray-tracing program BELLHOP^30^. Inputs to the model include the directional properties of the Lubell speaker^28^, a water depth of 5 m, and bottom properties associated with limestone (3000 m/s compressional speed; 2.4 g/cm^3^ density). Although the bottom/water interface was composed of white sand, simulations using a deep sand bottom yielded higher propagation losses with range than measured in the field, suggesting that the sand is only a thin layer over a larger coral/limestone basement. Figure 9 shows a contour map of the predicted propagation loss for both 500 Hz and 10 kHz. The propagation loss varies little with depth, except for higher losses a few cm above the ocean floor. A 15–20 dB SNR measured in the data at one meter suggests that acoustic enrichment broadcast at 15 dB SNR (Fig. 6c) would disappear into the background noise at ranges where the propagation loss was between 16 and 21 dB, which corresponds to 11–26 m range from the speaker. Simulations at lower frequencies (100 Hz) showed similar loss characteristics as the 500 Hz results shown here.

In summary, components of both the 500 to 1 kHz “low-frequency fish” band and 3–10 kHz “crustacean” band had similar detection ranges, since both had similar SNR and propagation properties. However, the SNR for frequencies below 1 kHz drops sharply (down to 5 dB SNR at 500 Hz), so at 21 m range, any detectable components of the acoustic playback are above 1 kHz. The 5 dB SNR of the ultrasonic component is predicted to merge into the ambient background at only 2–3 m range.

Acoustic modeling also showed that the acoustic particle velocity of the broadcast was typically proportional to the pressure field at ranges more than a few wavelengths from the source, except within a wavelength of a reflecting boundary (e.g., the surface). Thus, the 21 m propagation range estimated here is consistent for both pressure and particle velocity, since the lowest detectable frequency (1 kHz) has a 1.5 m wavelength, many times smaller than the source distance. This consistency in detection distance is an important point, given that larvae are expected to be more sensitive to particle velocity than acoustic pressure (e.g., fish larvae in ref. ^31^). These conclusions are consistent with experimental guidance^32^ and recent high-quality field measurements^33^.

### Settlement surfaces

Three types of artificial structure were deployed at the experimental site: the coral settlement module (CSM), the microhabitat cinderblock (MCB), and the fish habitat module (FHM). Some MCB structures were coated with a synthetic “Brink” biofilm (MCB+), and the other MCB structures were not (MCB−). One group of co-authors (JR, HJ, JM) designed and built the CSM and FHM, and another group (NL, SK, DW) built the MCB structures and synthesized the Brink biofilms. The design and purpose of each structure is detailed below.

#### Coral settlement modules (CSMs)

**Fig. 10:**
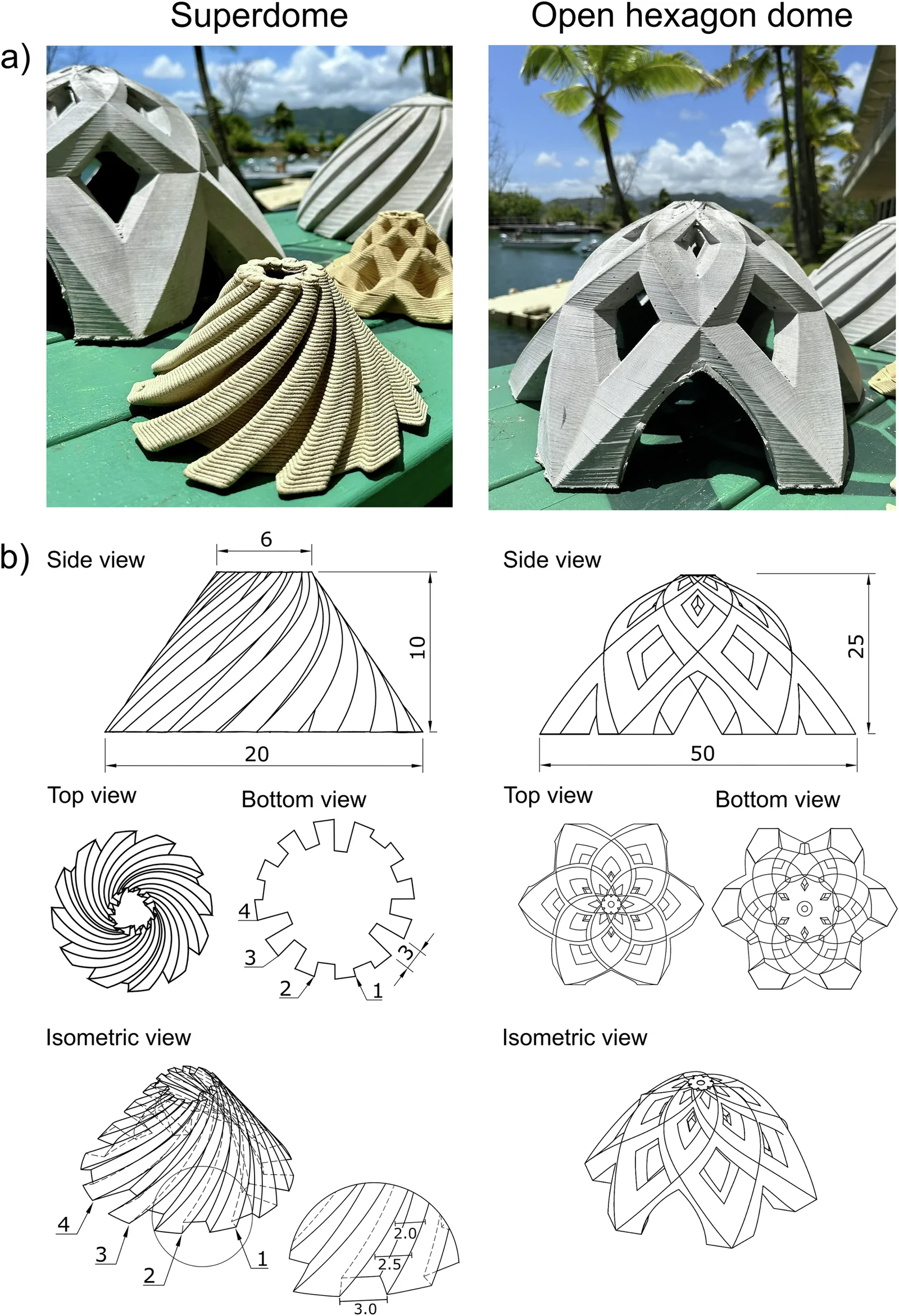
Design details for CSM and FHM structures. **a** Photographs of CSM (left) and FHM (right) designs. **b** Technical drawings of both designs (unit: cm). FHMs were not the focus of this study, but documented relative numbers of fish at active and inactive speakers.

The CSMs are 3D printed clay structures that have helical spiral grooves designed to provide recesses and other cryptic spaces to encourage coral larvae settlement^21^. Nineteen CSM units (Fig. 10, left column) were printed and placed on concrete cinder blocks at varying distances from the acoustic speaker. Each dome is about 20 cm in diameter and 10 cm high, with 816.74 cm^2^ of surface area, of which 461.74 cm^2^ (56%) consists of crevice surface area. Module designs were constructed in Blender v3.4 and printed in clay with the Delta Wasp 40100 3D clay printer (Wasp, Italy wirmid-fire pottery clay, Soldate 60, Aardvark Clay and Supplies, United States). Printed modules were air-dried for 10 days before firing in a kiln (Jupiter Sectional Kiln, L&L Kilns, United States).

#### Fish habitat modules (FHM)

The fish habitat modules (FHM) are also 3D printed structures that were deployed on-site for observing mature and larval fish. These larger “open hexagon domes” (Fig. 10, right column) had a diameter of 50 cm and a height of 25 cm and were cast in concrete using a silicone mold. Autonomous cameras were mounted 0.33 m away from the large openings visible at the base. During daylight hours, the cameras recorded five burst photos across five seconds, sampled every five minutes. Further details on the FHMs, cameras, and camera mounting systems are provided in ref. ^18^. The hexagon domes displayed little to no coral settlement and are not included in the settlement analysis.

#### Microhabitat cinder blocks (MCB)

**Fig. 11:**
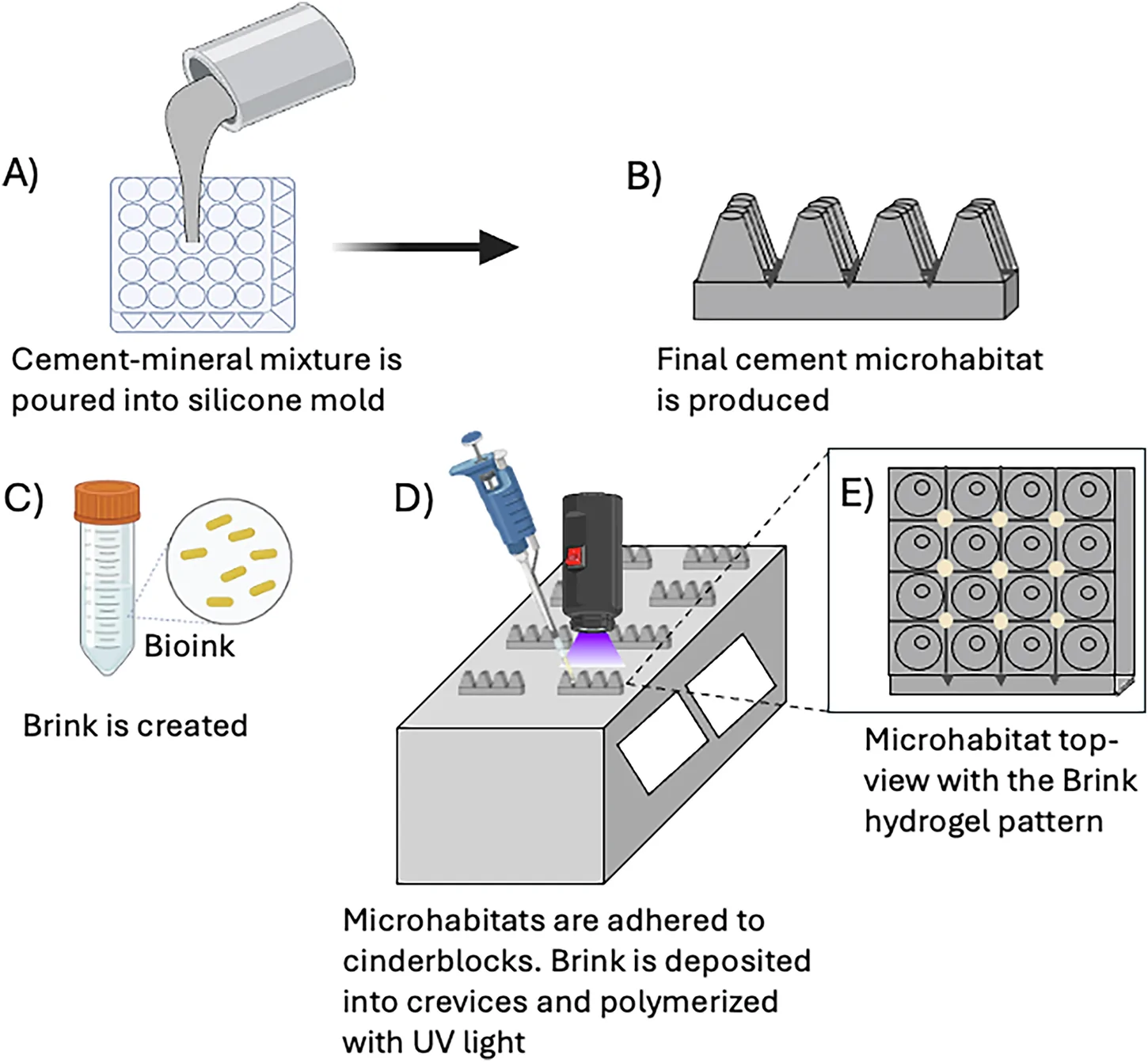
Development and integration process of microhabitat cinder blocks (MCB). **A** Cement-mineral mixture is poured into a silicone mold and left to dry for 48 h. **B** After drying, the final product is retrieved from the mold, and an MH baseplate is produced. **C** Brink is developed by combining the bacterial strain, *T. euphylliae*, and a combination of biopolymers (PEGDA-GelMA). **D** MHs (*n* = 8) are adhered to the remaining unconditioned cinder blocks (*n* = 6), where Brink is deposited in the interstitial spaces of the MHs, before being polymerized with UV light. **E** Top view of a single MH unit displaying the pattern of nine Brink hydrogel [beige] dots in the interstitial spaces.

Each MCB unit contains multiple “Volcano” microhabitat (MH) baseplates, designed to enhance larvae settlement in the presence of currents (μm to mm scale). The dimensions of the square baseplate of an individual MH unit were 31 × 31 mm, with the cylindrical cone volcano heights set to 10 mm with a cone base diameter of 7.75 mm, resulting in 16 evenly spaced volcanoes on the baseplate in a 4 by 4 grid. Crevices were debossed into the base (0.5 mm × 1.5 mm) to provide cryptic regions for larvae to settle. The total surface area of one volcano MH was 46.23 cm^2^, while the projected planar surface area of the baseplate was roughly 10 cm^2^ (Fig. 11B).

Fusion360 design software was used to create the master MH model, which was then 3D printed in a bioplastic polymer (PLA) using a PrusaMini printer. This model was then placed into a custom 3D printed mold frame, and a degassed silicone mix (ECOFLEX 00-35) was poured into the mold cavity, over the PLA model, and left to cure, leaving the remaining negative mold. MH fabrication was implemented with a scalable molding approach using a custom Portland cement mixture (60%) enriched with a mineral sand mix (20%) and calcium carbonate (20% limestone). Water was added until the concrete mix gained a surface sheen when the container was tapped several times to settle. The concrete was then poured into the master mold and tapped, scraped, and rotated to release air and prevent bubbles in the MH. The unit was cured for 2̃ days before being removed from the mold to allow for strengthening. After the drying period was complete, MHs were rinsed several times over three days to remove any particulates.

A MCB unit was created when eight MHs were glued with reef-safe aquaria glue onto cinder blocks, spaced into two columns of four (Figs. 7C and 11D). An MCB unit with eight baseplates yielded a combined MH area of 370.35 cm^2^ per unit.

#### Brink application to MCB+ blocks

The settlement-inducing bacterial strain, *Thalassotalea euphylliae* (H1), was previously isolated and described by Kewalo Marine Laboratory from tissues of a native Hawaiian coral, *Montipora capitata*^22,34–38^. *T. euphylliae* (H1) was recently used to develop a living bacteria reef ink (Brink, see details in ref. ^22^). Briefly, Brink was prepared as described previously by streaking bacterial strains from glycerol stocks onto 1/2 filtered seawater (FSW) tryptone (1/2 FSWt) and maintained at 25 ^∘^C for 24 h. About 200 mL of dense liquid cultures (1/2 FSWt) were grown for 24 h at 25 ^∘^C on a shaker at 170 rpm^22,35,36^.

The liquid culture was spun (5000×*g*, 20 min) to collect the bacterial pellet, which was resuspended in 200 mL of 10% 1/2 FSWt media diluted in filtered sea water at 1.5 OD600. Brink coatings were optimized and produced according to ref. ^22^, using a photocrosslinkable biopolmyer mixture made from poly(ethylene glycol)diacrylate (PEGDA) at 30% and gelatin methacrylate (GelMA) at 15% stock solutions (Millipore-Sigma, St. Louis, MO). Here, we developed our Brink coating by mixing 2 separate aliquots of 500 μl of PEGDA (30%), 500 ul of GelMA (15%), 500  ul of bacteria solution, and 187.5 ul of the photoinitiator lithium phenyl-2,4,6 trimethylbenzoylphosphinate (LAP) (TCI America^TM^) at 4%.

Six MCB− units were deployed at the site(*n* = 3) (*n *= 3) 10 days before acoustic playbacks, to condition with a natural biofilm before the start of the experiment(Fig. 1). Another six MCB+ units had 60 μl of Brink deposited in nine dots at the crevice intersection points of each MH on the block. Photopolymerization was induced via a UV light source (ThorLabs) (405 nm) for 30 s at an irradiance intensity of 17 mW/cm^−2^ (Fig. 11D), which was optimized to facilitate successful cross-linking, while minimizing potential cell damage^22,39^. Directly after photopolymerization, the MCB+ were placed in a large holding bin containing filtered sea water (1 um filtered), until transferred to the deployment site by boat, three days before coral spawning.

### Site layout

Figure 1 displays the layout of all 37 structures deployed in July 2024 at Reef 3. Four different types of structure were deployed at the site: 19 CSMs (upper case labels), six MCB− and six MCB+ structures (both lower case labels), and six fish habitat modules (FHM). Figure 1 also shows the location of the active (playback speaker) and the inactive (control) speaker placed 42-m away.

CSMs were placed along the two transect lines. One line (Transect XX) ran parallel to a large fringing reef, with CSMs placed at distances of 5.8, 11.6, 17.1, 22.9, 28.7, 34.4, and 39.9 m from the active speaker. The second line (Transect YY) was placed to act as an independent gradient test, beginning at the active speaker and then stretched along a line 60^∘^ relative to the first transect line, away from the fringing reef, with domes placed at distances of 3.0, 5.8, 8.2, 11.6, 17.1, and 22.9 m from the active speaker. The distances along this latter line were shorter than the first line, as the domes would have run into natural reef outcroppings to the north of both sites. Transect YY was not run directly west of the playback speaker due to the presence of a reef 20 m away.

Finally, three additional CSMs were placed symmetrically around both the active and inactive speakers at 1 m range, for a total of six additional CSMs. The “L” and “K” units around the playback speaker were arbitrarily assigned to Transect XX, and the “M” unit was assigned to Transect YY. All three CSMs surrounding the inactive speaker were assigned to Transect XX.

The layout also had three MCB+ and three MCB- units deployed in a ring with a radius of 1 m around the playback speaker. Additional pairs of MCB+ and MCB− units were placed at distances of 10, 20, and 30 m from the playback speaker, with each MCB+ and MCB- pair separated by approximately 1 m (Fig. 1).

We also deployed six fish habitat modules (FHMs) and two autonomous cameras to characterize fish presence at distances of 2 and 42 m from the speaker. One camera and three FHMs were deployed in a 2-m radius around each active and control speaker.

### Coral larvae census procedure

Coral larval settlement was measured one and two weeks after the new moon (July 11th and 18th, 2024) using SCUBA operations. Individual coral larvae were counted using a handheld blue light source (Sola Nightsea Light) and a yellow filter (via green fluorescent protein (GFP) response) on MHs. Larval settlement that occurred on MCB units but not inside volcano MHs were documented, but not counted towards the total settlement numbers in the survey. CSM counts were conducted only once, two weeks after the new moon, due to the larger logistical effort required to survey the complex surface geometry of the 19 structures. Species identification was not attempted as settled larvae are difficult to identify outside of laboratory conditions, and due to the need for in situ counting, it wasn’t possible to do a blind count (the divers were aware of the distance to speaker). However, in Kāneohe Bay, coral assemblages are dominated by a few species, including *Montipora capitata* and *Pocillopora acuta*. *M. capitata* participates in synchronized broadcast spawning events around the new moon^40^, whereas *P. acuta* primarily releases brooded larvae in cycles associated with the full moon^41^.

### Statistical methods

#### Regression analysis

Generalized linear models were used to isolate and evaluate the effects of various factors on coral settlement. Separate multivariate zero-inflated Poisson regression analyses were conducted for both CSM and MCB units, to determine whether any derived distance-dependent effects were consistent between the surfaces.

The dependent variable for both models is the raw integer counts of larval settlement on each surface. Coral settlement counts across all eight MHs on a single MCB were summed and treated as one independent sample (treatment). Given the large number of null counts, a zero-inflated Poisson (ZIP) was selected as the model distribution, with *z*_0_ designating the probability of a count being drawn from a “zero” generating process instead of a Poisson process. The Poisson rate parameter *λ* was then determined by a linear regression on the independent variables, including an intercept value, using a log link function^42^.

The independent variables for the CSM regression were (1) a continuous variable *r* representing the distance in meters of a CSM unit to the active speaker, and (2) a categorical variable **TRANSECT** assigning the CSM to Transect XX or YY, to see whether the replicate transects had systematic differences.

The regression formula for the CSM surfaces thus becomes

1 $$\ln [{\lambda }_{CSM}(r,{{{\bf{TRANSECT}}}})] \sim {b}_{0,CSM}+{b}_{r,CSM}\cdot r+{b}_{transect}\cdot {{{\bf{TRANSECT}}}}$$

with *b*_0,CSM_, *b*_*r*,CSM_, and *b*_transect_ being the regression coefficients for the intercept, *r*, and **TRANSECT** respectively, and *λ*_CSM_ being the Poisson rate parameter to be estimated for CSM surfaces.

The MCB regression retained distance as a continuous independent variable, but used a categorical variable **BRINK** to distinguish an MCB+ unit from an MCB−. **TRANSECT** was not used as a variable, as all MH structures were placed along Transect XX. The MCB regression becomes

2 $$ln[{\lambda }_{{{\mathrm{MCB}}}}(r,{{{\bf{BRINK}}}})] \sim {b}_{0,{{{\mathrm{BRINK}}}}}+{b}_{r,{{{\mathrm{MH}}}}}\cdot r+{b}_{{{\mathrm{BRINK}}}}\cdot {{{\bf{BRINK}}}}$$

Assuming a non-informative prior, the regressions were solved using the Bayesian generalized multivariate model **brms** package^43^ in the statistical software package R^44^. The package provides an interface for fitting Bayesian regression models using the probabilistic programming language Stan, which in turn uses Hamiltonian Monte Carlo (HMC) sampling algorithms to solve multidimensional integrals^45^. Each regression ran four chains with 4000 iterations each, of which 2000 were used for the “warm up” phase, using a target average acceptance probability (**adaptive**_**delta**) of 0.95. Models with interactive terms between **BRINK,TRANSECT**, and *r* were also tested, and the leave-one-outcross-validation (LOOCV) metric^46^ evaluated whether to include these additional terms in the model.

The 95% credible intervals of each regression coefficient were constructed from the 2000 posterior distribution samples using the **posterior_epred** command in R. Intervals that excluded zero were interpreted as indicating a statistically significant effect of that coefficient on settlement density. Prediction curves for any distance effect were generated using 4000 posterior samples evaluated along 50 distance points evenly spaced between 1 and 50 m. To allow predicted fits between structure types to be compared directly, predicted raw counts were divided by the surface area of the settlement surface to yield a settlement density. For example, the CSMs have a surface area of 817 cm^2^ (including crevices), while all MH on a single MCB have a combined surface area of 370 cm^2^.

All graphics were plotted using MATLAB (R2022b) and the ggplot2^47^ and dplyr^48^ packages in R v4.4.1^44^.

### Fish presence analysis

Complete details on the cameras and visual analysis of images collected near the active and control speakers are presented in ref. ^18^. We used an open-source camera system based on the ESP32-S chip and OV2640 camera with a 2MP 1600 × 1200 resolution and 65-degree field of view. A custom “Kilocam” circuit board (Ecologis Consulting, Inc.) uses an Arduino microcontroller to act as a scheduler and intervalometer for activating the ESP32-CAM image acquisition. Cameras were mounted 0.33 m from the entrance of an FHM using rebar, and every 5 min during daylight hours they gathered five images over a 3-s window, under the assumption that most herbivorous fish grazing occurs during daylight hours^49,50^. Images were manually analyzed for both fish and fish larvae. The “MaxN” metric, which computes the maximum number of fish visible in a single photo taken over a time interval, is used as a proxy for fish presence, as it removes the possibility for double-counting across multiple photos^51^. Another metric, “mean counts per camera” (CC), was also documented to capture a measure of fish persistence in an area. A non-parametric Wilcoxon rank-sum test explored whether a statistically significant difference existed between the treatment and control metrics. Zero-inflated Poisson regressions on the MaxN metric were also performed in a manner identical to that described in the previous subsection, with **TREATMENT** being an independent categorical variable indicating whether the active or control speaker is being modeled, and **MoonPhase** an independent continuous variable encoding moon phase.

The regression formula for the mean MaxN value for mature fish (MF) is thus

3 $${\ln}[{{\lambda }_{{{\mathrm{MF}}}}}] \sim {b}_{{{{\mathrm{MF}}}},0}+{b}_{{{\mathrm{MF}}}},{{{\mathrm{MoonPhase}}}}\cdot {{{\bf{MoonPhase}}}}+{b}_{{{\mathrm{MF}}}},{{{\mathrm{treatment}}}}\cdot {{{\bf{TREATMENT}}}}$$

with *b*_MF,0_, *b*_MF,MoonPhase_, and *b*_MF,treatment_ being the regression coefficients for the intercept, moon phase, and treatment site respectively.

## Conclusion

An integrated field test of three techniques for boosting coral larval settlement during natural spawning found robust and statistically significant effect sizes on coral larvae settlement for two of them: synthetic “Brink” biofilms, and customized settlement structures. Synthetic biofilm-coated surfaces (MCB+) resulted in coral settlement, while corresponding control surfaces (MCB−) showed no settlement at all. CSM surfaces displayed higher settlement densities than MCB. For practical applications, these surfaces are not exclusive, and a scaled-up CSM design is currently incorporating smaller MCB+ structures at spots along its surface to leverage the settlement-enhancing benefits of both techniques.

This study also identified a statistically significant effect of settlement with respect to distance from an acoustic enrichment speaker. However, since these results arose from a single deployment using a single speaker, alternative explanations for the observed distance effect remain viable. These explanations include the possibility of a random settlement hotspot that may be generated by natural chemical or acoustic cues released from natural reef structures that happened to coincide with the placement of the acoustic speaker. Another alternative explanation is that fish herbivory was greater near the acoustic speaker, due to the high concentration of structures there. Evidence against this latter explanation was provided by autonomous cameras that showed no significant difference in fish presence at 1 or 42 m away from the speaker, although there was a difference in fish larvae presence near the speaker for a few days surrounding the full moon. Full details of that analysis are provided in ref. ^18^.

A single AE deployment also cannot address seasonal or genetic variability in potential organism responses; nor can it determine whether acoustic enrichment would work under less benign oceanographic conditions with higher currents and/or wave action. Therefore, additional acoustic enrichment experiments, with a speaker in different locations, would be required to reduce the possibility of the observed distance effects being the result of a random settlement hotspot, and to demonstrate replication across a wider variety of locations that span different ocean conditions and reef environments. Acoustic recordings from other locations should also be randomly seeded in the replicate deployments to confirm that any attractant effect arises from coral reef ambient sound and not playback artifacts associated with a particular recording.

Based on lessons learned from this experiment, we have several recommendations for future deployments testing these settlement technologies, which expand on the current layout:

- The biggest single change we would advise is better transect design, to better capture confounding spatial and/or environmental variability. The transects should be arranged so that the speaker is centered inside all structure locations, with at least two transect lines deployed in parallel with a fringing reef, one traveling east from the speaker, and the other west, to ensure equal exposure to potential natural acoustic or chemical cues in the environment.
- The ends of each transect (furthest from the speaker) should have a small ring of structures that mirror the arrangement of structures around the speaker, to counter the possibility that more fish are being attracted to the speaker site due to a higher density of structures. No specific chemical link between higher fish presence and coral larvae response seems to have been identified in the literature, but future experiments in this vein might consider collecting water samples at opposite ends of transects to test for possible differences in chemical cue levels.
- Acoustic vector sensors should be deployed for at least one deployment at a new location to confirm that acoustic pressure and particle velocity are proportional to each other, validating acoustic modeling predictions.
- Sound recordings should include frequency components above 1 kHz, even up to the ultrasonic range, as shown in Fig. 6c. Acoustic recordings monitoring the deployment should measure and report levels for frequency ranges up to at least 30 kHz, for both the ambient and playback monitoring. Individual deployments that emphasize broadcasting energy at frequencies either above or below 1 kHz would also help provide insight into what features of ambient coral sound are most relevant to coral larvae settlement response.

The three approaches tested show enough promise to justify up-scaled tests on larger structures over larger areas. The scale of this field test (tens of meters) suggests that all approaches have the potential to be scaled up cost-effectively; however, one issue AE will face is the challenge of providing sufficient power to broadcast sound over a significant region, particularly in remote or surf-prone areas. Fortunately, our results suggest that AE needs only to be active 1 week out of the month for the case of coral larvae in Kāneohe Bay.

## Supplementary information

Supplementary Information

Transparent Peer Review file

## Data Availability

The authors declare that all data supporting the findings of this study are available within the paper as Tables 1 and 2
