# Supplementary material for: In situ enhancement of coral larval settlement using acoustic enrichment, engineered habitats and living materials
Source: Commun Biol. 2026 Oct 8;9:1288. doi: 10.1038/s42003-026-10932-3 (PMC13650121; doi:10.1038/s42003-026-10932-3)
Supplement: Supplementary file 1 [file 42003_2026_10932_MOESM1_ESM.pdf]

Supplementary Information for *In situ* enhancement of coral larval settlement using acoustic enrichment, engineered habitats and living materials

Aaron Thode[1*], Natalie Levy[2], Océane Boulais[1], Samapti Kundu[2], Marnie Freckelton[3], Daniel Schar[4], Michael Hadfield[3], Jessica Reichert[4], Hendrikje Jorissen[4], Netanel Kramer[2], Joshua Levy[5], Kira Hughes[4], the R3D consortium†[6], Joshua Madin[4], Daniel Wangpraseurt[2]

[1*]Marine Physical Laboratory, Scripps Institution of Oceanography, University of California San Diego, 9500 Gilman Dr, La Jolla, 92109, CA, USA.
[2]Reef Ecophysiology and Engineering Laboratory, Scripps Institution of Oceanography, University of California San Diego, 9500 Gilman Dr, La Jolla, 92109, CA, USA.
[3]Kewalo Marine Laboratory, University of Hawai'i, 41 Ahui St., Honolulu, 96813, HI, USA.
[4]Hawai'i Institute of Marine Biology, University of Hawai'i at Mānoa, 46-007 Lilipuna Rd., Kāneohe, 96744, HI, USA.
[5]Applied Research Laboratory, University of Hawai'i, 2800 Woodlawn Dr., Suite 263, Honolulu, 96822, HI, USA.
[6]† Please see Author Contributions for full consortium author list for Rapid Resilient Reefs for Coastal Defense (R3D).

*Corresponding author(s). E-mail(s): athode@ucsd.edu;
Contributing authors: nrlevy@ucsd.edu; oboulais@ucsd.edu; s1kundu@ucsd.edu; marnief@hawaii.edu; schar@hawaii.edu; hadfield@hawaii.edu; jhaver@hawaii.edu; jorissen@hawaii.edu; nkramer@ucsd.edu; levyjosh@arl.hawaii.edu; kirawa@hawaii.edu; jmadin@hawaii.edu; dwangpraseurt@ucsd.edu;

Supplementary background on coral larvae recruitment techniques

Acoustic enrichment

Acoustic enrichment (AE) is a technique whereby sounds produced by healthy reefs are broadcast by an underwater speaker to encourage coral larvae settlement in a different area. Many coral reef taxa have a planktonic larval phase, during which larvae settle in response to environmental cues such as light, chemical signals, and hydrodynamics [1–3]. Sound is increasingly recognized as another important settlement cue. Early studies showed fish larvae use reef ambient sound to orient toward suitable habitats [4, 5], with acoustic enrichment increasing fish recruitment by 50% on degraded reefs [6].

Field experiments have shown individual coral larvae also respond to reef ambient sound, independent of light and chemical cues [7]. Off the Virgin Islands *Porites astreoides* larvae have demonstrated settling behavior in response to acoustic stimuli [8]. More recent field experiments observing captured larvae in glass containers show acoustic enrichment significantly increases coral larvae settlement behavior, with effects measurable up to 30 meters from the source[9] .

Customized settlement surfaces

The structural complexity of artificial reef substrates plays a critical role in enhancing coral settlement[10, 11]. Engineered reef structures, such as artificial reefs, settlement tiles, and 3D-printed substrates, provide essential habitat complexity that facilitates benthic larval recruitment [12]. Coral larvae readily settle on artificial structures ranging from breakwaters to shipwrecks, with cryptic substrates often preferred for their protective microhabitats [13, 14]. Structural complexity has been shown to improve settlement rates and post-settlement survival. For example, [15] demonstrated that ceramic tiles with complex surfaces supported higher coral settlement and survival rates compared to simpler substrates. Similarly, [16] found that 3D-printed reef structures enhanced settlement space and increased biodiversity in recovering reef systems.

These findings have motivated the development of optimized artificial habitats to improve recruitment efficiency [17]. Recent research has emphasized the importance of incorporating specific surface textures and spatial patterning at sub-meter scales to enhance larval entrapment and retention [18–20]. At smaller spatial scales, cm-scale topographical features provide refuge from predation and competition while maintaining access to light[21]. Studies have shown that these features improve early survival rates, with [22] demonstrating that small-scale refugia reduced predation pressure on coral recruits, with survival rates exceeding 55% for recruits settled in narrow grooves after one year[23]. Recent work by one of our co-authors (JR) found that helix recesses in 3-D printed structures increased settlement 80-fold and post-settlement survival over a year by 20–50-fold. [21]. These helical recesses have been incorporated into "coral settlement modules" (CSM)[21], one of the two artificial surface types used in this study, described in more detail in the Methods. The other artificial surface type used in this study is the "volcano microhabitat", a grid of cm-scale size cylindrical cones designed to disrupt current flow and provide easier settlement opportunities for drifting[17].

Synthetic biofilms

Chemical cues play an important role in recruiting coral reef larvae[24, 25]. Biohybrid substrates, which combine artificial substrates with living components, provide still another avenue for enhancing coral recruitment and ecosystem resilience [26–29]. Certain marine bacteria have been identified as key drivers of larval settlement, producing bioactive compounds that induce coral attachment and metamorphosis [30–33]. For example, previous studies have demonstrated that certain strains of marine bacteria—like *Cellulophaga lytica* [34–36], and *Thalassotalea euphylliae* [35–37]—produce lipopolysaccharides (LPS) that induce benthic larval settlement and metamorphosis [35]. Innovative biofabrication approaches, such as the development of living bacteria-driven materials, have been explored to induce larval settlement [26–28, 36, 38, 39], enabling new possibilities for coral restoration. Building on this concept, several of our co-authors (NL, SK, DW) developed a bacterial reef ink ('Brink') for the fabrication of a settlement-inducing microbial coating on top of restoration substrates [36].

Supplementary methods

Evaluating algae coverage across site

This section details an ancillary photography analysis into whether fleshy algal cover varied with distance from the speaker. If true, changes in algal coverage with distance would provide an alternative explanation for the observed differences in coral settlement with distance from the source.

To estimate algal coverage and its potential correlation with coral settlement on MCB units, photographs were taken two weeks after deploying the structures (July 18th, 2024), and then reassessed a month later (August 20th). Any appearance of crustose coralline algae (CCA) was not included in the estimate.

Algal coverage estimates on the MHs were conducted on both July 18 and August 20th, using Adobe Photoshop 2025 (© Adobe) on a subset of MHs from each treatment (n= 4). Using the color range tool, we calculated the proportion of each image occupied by specific colors indicative of algal presence. First, we used the eyedropper tool to target areas of uncovered substrate, then adjusted the fuzziness setting to include similar hues. Once highlighted, this color range could be visualized on a separate layer to enhance clarity. Subsequently, we inverted the selection to focus on regions that were not substrate-covered. The histogram function provided the number of selected pixels, which we then divided by the total pixel count in each image to obtain the percentage of algal coverage for each MCB.

The CSMs were photographed on August 20th, 16 days after the August new moon and over 30 days after all acoustic playbacks had ceased. The fraction of the surface covered by fleshy algae on the CSMs was estimated visually by HIMB researchers, using photos taken from all sides of a CSM unit(n= 3 photos per CSM). The results were averaged to provide a "mean fraction" of coverage for a CSM surface. The estimated uncertainty in fractional cover for both techniques is ± 0.1.

Tables 1 and 2 from the manuscript are reproduced below as Supplemental Tables 1 and 2. The last two columns in Supplemental Table 1 display the fractional non-CCA

algal cover on the MCB, estimated from photographs on July 18th and the follow-on survey on August 20th. The final column of Supplemental Table 2 displays the estimated algal cover on the CSMs on August 20.

Supplemental Figure 1 displays a scatterplot between playback speaker distance and algal cover for these three datasets, with the marker size of the plot points proportional to relative larval settlement on July 18th, the last day of the AE playback.

For the July 18 algal survey, Supplemental Fig. 1 and Supplemental Tables 1 and 2) show that most surfaces had algal coverages of 80% and above, but three MCB within 1 m of the speaker had between 70 and 80% coverage (Supplemental Table 1), and these units were associated with relatively large larval densities.

By the August 30 data all the MHs and most of the CSMs had nearly 100% algal coverage. Two CSM structures within a meter of the speaker did display low estimated algal coverage on that date, but one of these CSMs (labels **L**) also had no larval counts on July 18th.

A Spearman rank correlation test was independently applied between algal cover and coral counts on MCB+ units for July 18th, the one date where both algal cover and count data were available. The MCB- units were not used in the analysis, to ensure that settlement counts and algal estimates were taken from the same location. The statistical significance of the correlation value was assessed by recomputing the correlation for all randomized perturbations of the six samples.

The correlation analysis found no significant linear correlation between algal coverage and distance. The coefficient between settlement count and algal cover (value; *p-value*) for the MCB July 18 data is (0.091; *0.883*), or (-0.67; *0.3*) if a possible outlier MCB **a.1** is removed. We also recomputed the Spearman coefficient for two additional datasets where the algal estimates took place on different dates than the coral counts: (1) MH July 11th counts, reusing the July 18th algal cover data (0.088; *0.87*) ; and (2) CSM July 18th counts, using the August 20th algal coverage (0.10; *0.69*). In all cases, the measured linear correlation between algal cover and settlement density is poor and can easily arise from random chance (i.e., the null hypothesis of an uncorrelated relationship cannot be rejected).

References

[1] Moeller M, Nietzer S, Schils T, Schupp PJ. Coral larval settlement preferences linked to crustose coralline algae with distinct chemical and microbial signatures. Scientific Reports. 2021;11(1):14610.

[2] Pawlik JR. Chemical ecology of the settlement of benthic marine invertebrates. Oceanography and Marine Biology: An Annual Review. 1992;30:273–335.

[3] Hadfield MG, Paul VJ. Natural chemical cues for settlement and metamorphosis of marine invertebrate larvae. Marine Chemical Ecology. 2001;p. 431–461.

[4] Simpson S, Meekan M, McCauley R, Jeffs A. Attraction of settlement-stage coral reef fishes to reef noise. Marine Ecology Progress Series. 2004;276:263–268. https://doi.org/10.3354/meps276263.

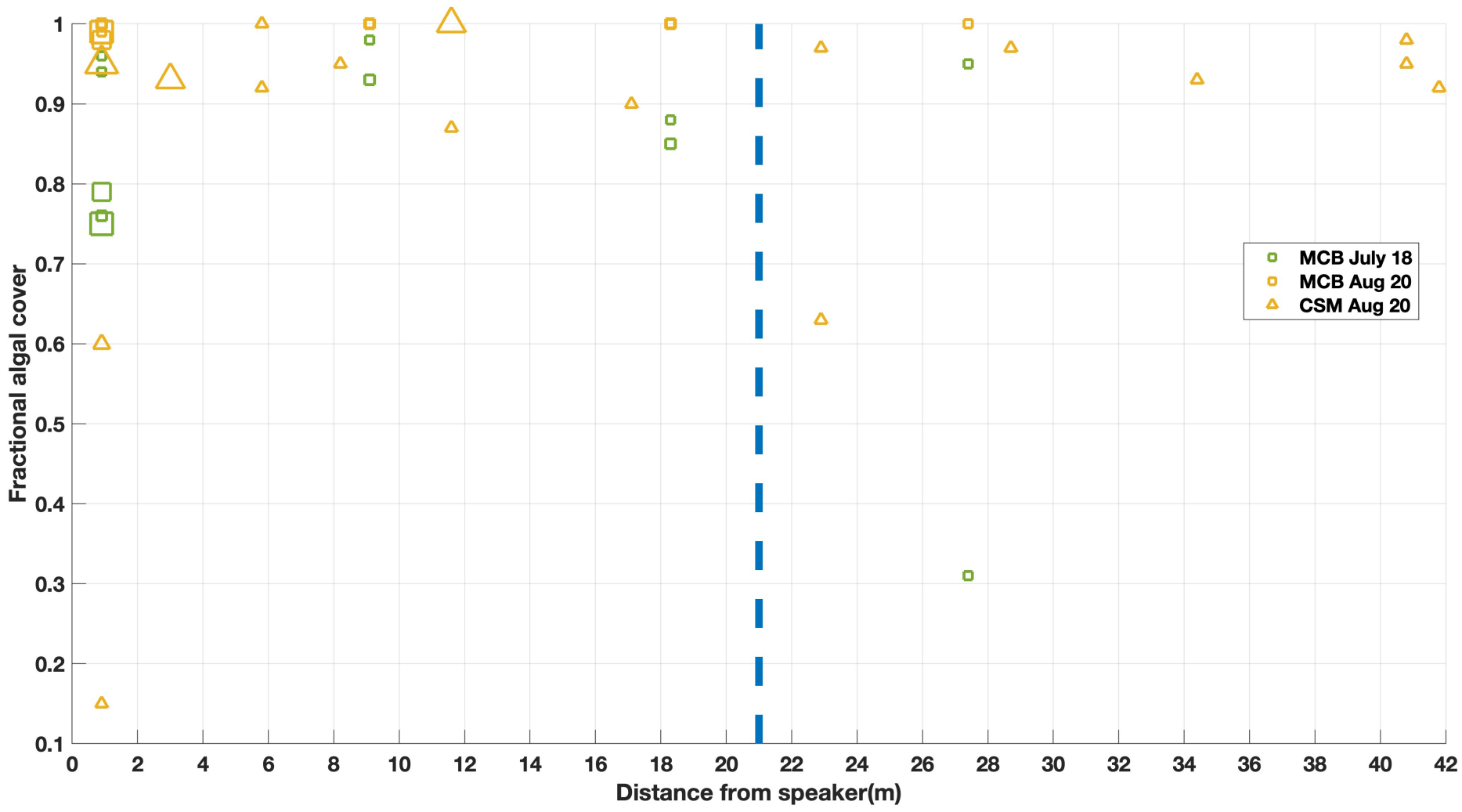

Supplemental Figure 1: **Scatterplot of surface algal cover fraction vs. distance from playback speaker (m) for three combinations of date and surface type**. Marker area is proportional to larval settlement counts on July 18th for that surface. The uncertainty in fractional cover is ± 0.1. The vertical dashed line is the estimated maximum range where AE playback sounds become indistinguishable from surrounding ambient sound (21 m).

[5] Tolimieri N, Haine O, Jeffs A, McCauley R, Montgomery J. Directional orientation of pomacentrid larvae to ambient reef sound. Coral Reefs. 2004;23:184–191.

[6] Gordon TA, Radford AN, Davidson IK, Barnes K, McCloskey K, Nedelec SL, et al. Acoustic enrichment can enhance fish community development on degraded coral reef habitat. Nature Communications. 2019;10(1):5414.

[7] Vermeij MJ, Marhaver KL, Huijbers CM, Nagelkerken I, Simpson SD. Coral larvae move toward reef sounds. PloS one. 2010;5(5):e10660.

[8] Lillis A, Apprill A, Suca JJ, Becker C, Llopiz JK, Mooney TA. Soundscapes influence the settlement of the common Caribbean coral Porites astreoides irrespective of light conditions. Royal Society Open Science. 2018;5(12):181358.

[9] Aoki N, Weiss B, Jézéquel Y, Zhang WG, Apprill A, Mooney TA. Soundscape enrichment increases larval settlement rates for the brooding coral *Porites astreoides*. Royal Society Open Science. 2024;11(3):231514. https://doi.org/10.1098/rsos.231514.

[10] Carlson RR, Crowder LB, Martin RE, Asner GP. The effect of reef morphology on coral recruitment at multiple spatial scales. Proceedings of the National Academy

of Sciences. 2024;121(4):e2311661121.

[11] Edmunds PJ. Coral recruitment: patterns and processes determining the dynamics of coral populations. Biological Reviews. 2023;98(6):1862–1886.

[12] Higgins E, Metaxas A, Scheibling RE. A systematic review of artificial reefs as platforms for coral reef research and conservation. PLoS One. 2022;17(1):e0261964.

[13] Burt J, Bartholomew A, Bauman A, Saif A, Sale PF. Coral recruitment and early benthic community development on several materials used in the construction of artificial reefs and breakwaters. Journal of Experimental Marine Biology and Ecology. 2009;373(1):72–78.

[14] Boström-Einarsson L, Babcock RC, Bayraktarov E, Ceccarelli D, Cook N, Ferse SC, et al. Coral restoration–A systematic review of current methods, successes, failures and future directions. PLoS One. 2020;15(1):e0226631.

[15] Fujiwara S, Kezuka D, Hagiwara K, Ishimori H, Tabata H. Effect of substratum structural complexity of coral seedlings on the settlement and post-settlement survivorship of coral settlers. In: Oceans. vol. 4. MDPI; 2022. p. 1–12.

[16] Loke LH, Ladle RJ, Bouma TJ, Todd PA. Creating complex habitats for restoration and reconciliation. Ecological Engineering. 2015;77:307–313.

[17] Levy N, Berman O, Yuval M, Loya Y, Treibitz T, Tarazi E, et al. Emerging 3D technologies for future reformation of coral reefs: Enhancing biodiversity using biomimetic structures based on designs by nature. Science of The Total Environment. 2022;830:154749.

[18] Hata T, Madin JS, Cumbo VR, Denny M, Figueiredo J, Harii S, et al. Coral larvae are poor swimmers and require fine-scale reef structure to settle. Scientific Reports. 2017;7(1):2249.

[19] Levenstein MA, Gysbers DJ, Marhaver KL, Kattom S, Tichy L, Quinlan Z, et al. Millimeter-scale topography facilitates coral larval settlement in wave-driven oscillatory flow. PLoS One. 2022;17(9):e0274088.

[20] Limer BD, Payne O, Brancato A, Mitchell M, Abu-Kubie C, Figueiredo J, et al. Life history and early ontogeny determine vertical swimming behaviors in the larvae of Caribbean corals. Journal of Experimental Marine Biology and Ecology. 2024;578:152035.

[21] Reichert J, Jorissen H, Drury C, Hancock JR, Haynes C, Nims AD, et al. Helix recesses boost coral larvae settlement and survival. Biological Conservation. 2025;311:111407. https://doi.org/https://doi.org/10.1016/j.biocon.2025.111407.

[22] Doropoulos C, Roff G, Bozec YM, Zupan M, Werminghausen J, Mumby PJ. Characterizing the ecological trade-offs throughout the early ontogeny of coral recruitment. Ecological Monographs. 2016;86(1):20–44.

[23] Randall CJ, Giuliano C, Heyward AJ, Negri AP. Enhancing coral survival on deployment devices with microrefugia. Frontiers in Marine Science. 2021;8:662263.

[24] Lecchini D, Nakamura Y. Use of chemical cues by coral reef animal larvae for habitat selection. Aquatic Biology. 2013;19(3):231–238.

[25] Rittschof D, Forward Jr R, Cannon G, Welch J, McClary Jr M, Holm E, et al. Cues and context: larval responses to physical and chemical cues. Biofouling. 1998;12(1-3):31–44.

[26] Wangpraseurt D, You S, Sun Y, Chen S. Biomimetic 3D living materials powered by microorganisms. Trends in Biotechnology. 2022;40(7):843–857.

[27] Roger LM, Lewinski NA, Putnam HM, Roxbury D, Tresguerres M, Wangpraseurt D. Nanobiotech engineering for future coral reefs. One Earth. 2023;6(7):778–789.

[28] Roger L, Lewinski N, Putnam H, Chen S, Roxbury D, Tresguerres M, et al. Nanotechnology for coral reef conservation, restoration and rehabilitation. Nature Nanotechnology. 2023;18(8):831–833.

[29] Quinlan ZA, Bennett MJ, Arts MG, Levenstein M, Flores D, Tholen HM, et al. Coral larval settlement induction using tissue-associated and exuded coralline algae metabolites and the identification of putative chemical cues. Proceedings of the Royal Society B. 2023;290(2009):20231476.

[30] Sneed JM, Demko AM, Miller MW, Yi D, Moore BS, Agarwal V, et al. Coral settlement induction by tetrabromopyrrole is widespread among Caribbean corals and compound specific. Frontiers in Marine Science. 2024;10:1298518.

[31] Giorgi A, Monti M, Paul VJ, Sneed JM, Olson JB. Larvae from three Caribbean corals settle differently in response to crustose coralline algae and their bacterial communities. Marine Ecology Progress Series. 2024;751:53–69.

[32] Alker AT, Farrell MV, Demko AM, Purdy TN, Adak S, Moore BS, et al. Linking bacterial tetrabromopyrrole biosynthesis to coral metamorphosis. ISME communications. 2023;3(1):98.

[33] Sneed JM, Sharp KH, Ritchie KB, Paul VJ. The chemical cue tetrabromopyrrole from a biofilm bacterium induces settlement of multiple Caribbean corals. Proceedings of the Royal Society B: Biological Sciences. 2014;281(1786):20133086.

[34] Asahina AY, Hadfield MG. Complete genome sequence of Cellulophaga lytica HI1 using PacBio single-molecule real-time sequencing. Genome announcements.

2014;2(6):10–1128.

[35] Freckelton ML, Nedved BT, Cai YS, Cao S, Turano H, Alegado RA, et al. Bacterial lipopolysaccharide induces settlement and metamorphosis in a marine larva. Proceedings of the National Academy of Sciences. 2022;119(18):e2200795119.

[36] Levy N, Kundu S, Freckelton M, Dinasquet J, Flores I, Galindo-Martínez CT, et al. Microbial living materials promote coral larval settlement. PNAS nexus. 2025;4(9):pgaf268.

[37] Summers S, Freckelton ML, Nedved BT, Rice SA, Hadfield MG. Full-genome sequence of Thalassotalea euphylliae H1, isolated from a Montipora capitata coral located in Hawai'i. Microbiology resource announcements. 2018;7(20):10–1128.

[38] Wangpraseurt D, Sun Y, You S, Chua ST, Noel SK, Willard HF, et al. Bioprinted living coral microenvironments mimicking coral-algal symbiosis. Advanced Functional Materials. 2022;32(35):2202273.

[39] Freckelton ML, Nedved BT, Hadfield MG. Bacterial envelope polysaccharide cues settlement and metamorphosis in the biofouling tubeworm Hydroides elegans. Communications Biology. 2024;7(1):883.

Supplemental Table 1: Settlement results on MCB surfaces measured one and two weeks after the new moon, expressed in terms of both raw counts and settlement density. The distance column shows the distance of a given MCB from the playback speaker in meters, and the last two columns indicate the fraction of the MCB surface covered by algae that was not crustose coralline algae (CCA). Surfaces that share the name letter label are equidistant from the playback speaker. A '0' label suffix indicates a MCB- structure and '1' suffix indicates a MCB+ (Brink treated) structure.

Block	July 11 Raw counts	July 18 Raw counts	July 11 counts/m^2	July 18 counts/m^2	Distance (m)	Algae July 18 Mean fraction	Algae August 20 Mean fraction
a.1	0	0	0.0	0.0	27.4	0.31	1.00
b.1	0	1	0.0	27.0	18.3	0.85	1.00
c.1	5	1	135.0	27.0	9.1	0.93	1.00
d.1	6	3	162.0	81.0	0.9	0.79	0.98
e.1	4	1	108.0	27.0	0.9	0.76	1.00
f.1	6	4	162.0	108.0	0.9	0.75	0.99
a.0	0	0	0.0	0.0	27.4	0.95	1.00
b.0	0	0	0.0	0.0	18.3	0.88	1.00
c.0	0	0	0.0	0.0	9.1	0.98	1.00
d.0	0	0	0.0	0.0	0.9	0.94	1.00
e.0	0	0	0.0	0.0	0.9	0.96	1.00
f.0	0	0	0.0	0.0	0.9	0.94	0.99

Supplemental Table 2: Settlement results on CSM surfaces measured on July 18th, expressed in terms of raw counts and settlement density. The distance column shows the distance of CSMs from playback speaker, and the last column indicates the fraction of CSMs covered by algae during an August 20th survey.

**CSM**	**Transect**	**July 18 Raw counts**	**July 18 counts/m^2**	**Distance (m)**	**Algae August 20 Mean fraction**
**A**	**XX**	2	24.5	41.8	0.92
**B**	**XX**	1	12.2	40.8	0.98
**C**	**XX**	0	0.0	40.8	0.95
**D**	**XX**	3	36.7	39.9	N/A
**E**	**XX**	1	12.2	34.4	0.93
**F**	**XX**	2	24.5	28.7	0.97
**G**	**XX**	1	12.2	22.9	0.97
**H**	**XX**	5	61.2	17.1	N/A
**I**	**XX**	19	232.6	11.6	1.00
**J**	**XX**	0	0.0	5.8	0.92
**K**	**XX**	22	269.4	0.9	0.95
**L**	**XX**	0	0.0	0.9	0.15
**M**	**YY**	8	98.0	0.9	0.60
**N**	**YY**	19	232.6	3.0	0.93
**O**	**YY**	1	12.2	5.8	1.00
**P**	**YY**	0	0.0	8.2	0.95
**Q**	**YY**	2	24.5	11.6	0.87
**R**	**YY**	0	0.0	17.1	0.90
**S**	**YY**	2	24.5	22.9	0.63
