# Supplementary material for: In situ enhancement of coral larval settlement using acoustic enrichment, engineered habitats and living materials
Source: Commun Biol. 2026 Oct 8;9:1288. doi: 10.1038/s42003-026-10932-3 (PMC13650121; doi:10.1038/s42003-026-10932-3)
Supplement: Supplementary file 2 [file 42003_2026_10932_MOESM2_ESM.pdf]

nature portfolio

Peer Review File

In-situ enhancement of coral larval settlement using acoustic enrichment, engineered habitats and living materials

Corresponding Author: Dr Aaron Thode

Parts of this Peer Review File have been redacted as indicated to remove third-party material.

This file contains all reviewer reports in order by version, followed by all author rebuttals in order by version.

Version 0:

Reviewer comments:

Reviewer #1

(Remarks to the Author)
Review of:
In situ enhancement of coral larval settlement using acoustic enrichment, engineered habitats and living materials
Thode and colleagues.

Overall, a very interesting study.
I have quite a few points that would need attention before I can recommend publishing, especially publishing in Nature Communications.

General Points:
One is that the text is generally confusing to read. The names of the devices/structures are a bit jargony (Brinks, CSMs, MHs, microhabitats, superdomes, mini superdome CSM) and they are used a bit interchangeably, or at least variably, making it all a bit hard to follow. And there is a decent amount of repetitive text. Perhaps this was cowritten? But the text needs a strong read/edit from just one (perhaps new) set of eyes to streamline the message.
Additionally, re: the multiple factors, while novel, contribute to this tangled storyline. While there is certainly potential in putting all methods together the writing needs to be improved to convey the details and nuances. Sure, it's easy to gain the topline message 'It all works' but which devices, how and precise setup are confusing. This could be separated into multiple papers, or if the authors want to keep it all together, they (or perhaps one person) need(s) to work on the writing.

Similarly, the figures are difficult to gain meaningful information from, and at times are a bit misleading. They need substantial work noted below.

Additionally, I found it very challenging experimentally that there were co-incident fish attraction structures, but this community was not quantified (there was some notation of that effort in the supplemental material but it was either left out, or it was in refence to the algae hypothesis). But the fish community clearly could impact the coral that they are measuring. Both in terms of attraction of coral (so a confounding effect to the sound) as well as a potential corallivore. So it's actually quite important in this case, because you are putting out structures that are to attract fish, and there is a wealth of papers on fish larvae being attracted to sound/reefs, and we know fish species can influence coral communities, through predation, nutrients, etc… that fish abundances and species should be quantified and noted here, at least as a potential explanatory variable.

Overall, the figures and story need to be consolidated. As it reads now, it is just too complicated (or maybe disparate) to figure out what is going on. The overall 'story' of integration is a good idea but it is not working as it is written. This is further confused by the authors varying how the refer to the various settlement structures (which in a way is the new ideas). The authors either need to separate into multiple papers (which may be impossible given the experimental design) or re-write and redesign the paper. But separating, if possible, several smaller papers would help streamline each individual message. Otherwise, this paper needs a lot of restructuring.

Additionally: The discussion of direct/indirect algal effects is interesting but feels very difficult to get at. They mention that

acoustic enrichment could attract more herbivores and make settlement surfaces more attractive, but you could also attract corallivores, leading to more direct post-settlement mortality… It seems like something the authors felt (or were told) to address but it is so different from the rest of the paper/not really substantiated in a meaningful way (to support the hypothesis).

Additionally, I found it very challenging experimentally that there were co-incident fish attraction structures, but this community was not quantified (there was some notation of that effort in the supplemental material but it was either left out, or it was in refence to the algae hypothesis). But the fish community clearly could impact the coral that they are measuring. Both in terms of attraction of coral (so a confounding effect to the sound) as well as a potential corallivore. So it's actually quite important in this case, because you are putting out structures that are to attract fish, and there is a wealth of papers on fish larvae being attracted to sound/reefs, and we know fish species can influence coral communities, through predation, nutrients, etc… that fish abundances and species should be quantified and noted here, at least as a potential explanatory variable.

Finally, one of the challenges of the experiment is that it was setup as both a dose- based and a paired treatment vs. no treatment (sound vs. no sound; brink vs. no brink) and given the variability (0-22 in some cases) it is really really hard to discriminate impact. For example, if you test between 1m CSM at sound vs. no sound, there's no effect (t test). But agreed, 1 m brink vs. not brink does show impact-increased settlement create (similarly overall brink vs not-brink shows a positive effect). But basically 3 to 4 points drives the distance/dose regression. Without those points (and still including data at 1 m) the regression is basically flat. So while there is a dose-based effect it is not a very strong signal at all (at least acoustically). I am wondering whether the sound is confusing things. One thing that might put this variable settlement into context is to address natural settlement expected in Kaneohe Bay or natural Hawaii settlement overall (although I'd guess the Bay would be quite different than ocean coastline).
Overall, I would suggest the authors carry out some separate focused experiments that address these parameters individually. And then focus on the design with more replication to better discern patterns.

Figure needs:
For example, Fig 1, yes while larval settlement / m2 is technically correct the numbers measured were far lower (so this is scaling up/estimating what the numbers would be if they were measuring a meter squared). Hence a bit disingenuous. Per cm^2 would be more reflective of the reality of the experiment.
Fig 2, lettering and symbols are very small making them hard to read without blowing up figure (for example, the compass has bigger letters and that's probably the least important part of the figure). Why is the title 'Relative' larval counts? Isn't it actual? It is really hard to tell the symbol different between the oval of the microhabitat only and the rectangle microhabitat with (in this case it's called 'bacteria reef ink (Brink)'). The inset pic is almost helpful but too small to discern actual setup. Overall results are really hard to dissent from this figure…
You should also label on the figure the YY and XX transect lines.
Fig 3. PDD or PDF? Seems to use both. Fonts are much smaller than Fig 1 and hard to read again.
Fig 4. Same comments. These could also be supplemental. They don't seem very critical to the results or methods (but support the methods…)
Fig 5. Similar to Fig 1. Using per meter squared seems to convey much higher settlement than actually observed. Also the filled points are actual data. Seems like the regression should not have points. Similarly (with respect to analyses) the regression slope seems basically driving by the first 1 meter settlement rates. If those are removed is the settlement basically flat? This would seem to indicate that that there is very little (or no effect) beyond 1 m. CSM is also the superdome, correct? Pls keep consistent. Further, data here should be presented as CSM Transect YY model (vs. 'measured')

Fig 6. Seems like this could be combined with Fig 2 or 10 as they are all setup/location based figures…
Fig 7. Natural reef sound (not noise). Noise has a very specific definition/connotation in and outside of the acoustics world. See Art popper's work, as well as Noise Matters by Haven Wiley.
Fig 9, again units are super small and hard to read. A scale bar would be helpful. Y axis is not labelled in A. Just some careless things here.
Fig 10. Can this be combined with Fig 2? Again, with all the variables it's hard to discern what actually occurred and I found myself flipping back and forth between this fig and fig 2.
You could probably just make one figure with the symbols all bigger (or put together as an a and a 'b' and the actual results. Notably, re: results, Table 1 is quite important but it's buried (but all the figures are integrated?). Overall, I find myself looking for a figure that actually shows the results, but it's not really included. Fig 2 is almost impossible to read and shading doesn't really help as you have to go back and forth to the colour bar to figure out what colour means high vs. low numbers. Fig 5 is inflated numbers. I know it's not how you ran your stats, but just some simple bar or scatter plots
Fig 11 is helpful but also so different from the rest of the paper. It really reflects that the paper seems to be written and integrated by a number of authors without a true story or voice. Also note here that the names of the structures are inconsistent with how they are referred to or labelled in other parts of the paper. Alternatively, put all the two 'structure' figures together (11 and 12) so that we see the settlement surfaces all in one place.
Similarly, there is a wealth of papers on larval reef fish being attracted to sound. But this is not addressed entirely (in a paper that is otherwise seeking to be integrative). I get that the authors want to make a second paper on this but there is a relationship between fish and coral. I would think an alternative hypothesis to the coral + sound is that the authors added structures + sound that may have recruited juvenile or adult (or larval fish). See work by Tim Gordon and Steve Simpson and colleagues. And these fish are constantly producing metabolomics and nutrients and chemical cues. And these chemical cues attract the coral larvae.
See Lecchini and related work below. Some are pasted below. (I am not sure what to do with the Dixson papers).
Lecchini D, Nakamura Y. Use of chemical cues by coral reef animal larvae for habitat selection. Aquatic Biology. 2013 Oct

24;19(3):231-8.
Pawlik JR. Induction of marine invertebrate larval settlement: evidence for chemical cues. Ecological roles of marine natural products. 1992:189-236.
Slattery, Marc. "Chemical cues in marine invertebrate larval settlement." Fouling Organisms of the Indian Ocean. CRC Press, 2020. 135-157.
Quinlan, Zachary A., et al. "Coral larval settlement induction using tissue-associated and exuded coralline algae metabolites and the identification of putative chemical cues." Proceedings of the Royal Society B 290.2009 (2023): 20231476.
Rittschof D, Forward Jr RB, Cannon G, Welch JM, McClary Jr M, Holm ER, Clare AS, Conova S, McKelvey LM, Bryan P, Van Dover CL. Cues and context: larval responses to physical and chemical cues. Biofouling. 1998 Jan 1;12(1-3):31-44.
Gerlach G, Atema J, Kingsford MJ, Black KP, Miller-Sims V. Smelling home can prevent dispersal of reef fish larvae. Proceedings of the national academy of sciences. 2007 Jan 16;104(3):858-63.

Sweatman, Hugh. "Field evidence that settling coral reef fish larvae detect resident fishes using dissolved chemical cues." Journal of Experimental Marine Biology and Ecology 124.3 (1988): 163-174.

Specific comments:
A small note here, the numbers on the manuscript are not actually line numbers. They don't match up with the lines of text. Making it annoyingly challenging to address specific comments. And perhaps challenging for the reviewer to convey edits to the author. This should be fixed on the next version.
088/90 – This sentence, especially the first have, needs to be rewritten. It is unclear.
"A key challenge remains the limited success of natural coral recovery, necessitating innovative solutions to support reef resilience."

96/98 – what debate about relative importance of natural reef restoration versus artificial reef deployment? You probably need more context or a reference here. Or maybe those are the following refs (~lines 101/102?) but are those refs 'debating' or just offering alternative ideas. In either case this point does not seem very directly relevant to the paper. Why not just talk about the importance of supporting/restoring reefs in peril?

109/110 – techniques (plural)

119/120 – remove noise. This is not correct use of the word (as noted above).

122 – ref #21 does not refer to the sentence about orientation. It's about 'auditory sensitivity'. Seems like incorrect reference.

123 – are the anthropogenic noise impacts relevant here? Seems off-topic. Suggest removing.

130 – Refs 25,26 seem incorrect. 26 is not coral larvae. 25 is a field experiment.

164 – I believe the authors mean to suggest 'sub-metre' scale. Metre seems too large.

167 – oddly this Reichart bioarxiv paper is not numbered. Further, it is not peer reviewed. I think generally all journals ask for peer review references. Certainly Nature. Pls remove.

187 – 46 is similarly a bioarxiv un peer reviewed paper. Basically a webpage. This should be removed
192 – This paragraph is confusing. Are the authors suggesting biohybrid substrates are a 4th 'emerging approach for enhancing coral recruitment'? or is this a continuation of the thoughts of the previous paragraph. Sorry, it is just unclear.

199/202 – this sentence/ point was just stated in the previous paragraph… odd. pls remove/fix.

203 – what is R3D?

232/238 – This is all very confusing. If this is well established, in can stay in the intro but needs a reference or two about your CSMs, or move this to the methods with appropriate methodological figures to describe. Otherwise, it is not enough information and out of context. But you are basically describing methods and it is confusing to follow.
243/4 – new moon spawning of what species? Fish and coral species spawning times generally vary by species. You seem to have an idea which species you are targeting...

Results
[in the first half of the Results, it seems like authors do not have enough data for statistical significance… so are mostly describing trends which are not really robust for peer-reviewed publication (if you are trying to make the point of "effect"). There is a lot about 'notable' and trends ('twice as high') but without robust stats to back up their apparent point.
281 – "The enhanced densities are notable up to 21 m range" is this significant? Or just "notable"? seems really vague here. Pls clean up language.

300 – Authors switched to present tense here. Pls keep consistent. So was there a statistical difference here? Or general trends?

396 – you mean 'lack of settlers,' not lack of counts I think. Unclear.

403 paragraph – These results are unclear. For one, I am not sure how to pull out the 2.5x greater point from Fig 3c. My instinct is to go to the nearby figure 2 which has results (but is hard to read) but YY and XX are not labelled on that figure. Nor are the site letters. Fig 5 is largely a model. So discerning what you are talking about is a bit challenging. In the following sentence, I do not think site "I" is on the 1 m circle (as shown in Fig 10) so this sentence does not make sense. Finally, if you changed the data points, and found different results (last sentences) wouldn't that imply that the results (differences between transects) are real?

418 paragraph. Agreed, the unbalanced treatment is really unfortunate and effects the experimental design, and thus our interpretation of the results. The authors make the somewhat half developed point about algae. But we know coral larvae respond to chemical cues and structure. Thus making a design that is not balance in structure (and the cues that can then co-develop from attracted fish) really does impair how we determine the acoustic results. This is indeed unfortunate. I am not sure what to do about this. I would generally say this test should be repeated properly. At the minimum, it would be helpful to provide the fish data along with these results.
Also, note here, you are referring to the MHs as cinder blocks. This flipflopping of naming convention is very challenging to a reader to follow. Maybe call them cinder blocks+MH+Brink…and cinder block+MH (or some more explicit label).
442/3 – you note the fish data are in the SM but I did not see it there. Pls provide or clarify.

631/2 – It is great that you measured the received levels at this distance from 1-15 kHz. But wouldn't the acoustic energy be frequency dependent? Ie some frequencies may be louder the others (ie is your sound treatment flat?). Also, what about frequencies below 1 kHz? Seems like those should be quantified as well. There is a wealth of data that invertebrates respond to low frequency sound. Seems like you are missing analyses of the important frequency range.
655 - 'had a unique playlist'
658 – 'had a master schedule'
782 – the challenge here is that the "open-hexagon CSMs, used as artificial fish habitats" were part of this experiment… Not accounting for their potential influence to the community (given that we know fish respond to sound) seems like a real oversight and should be addressed more completely.

821/8 – this 2 m ring does not seem clear (or does not appear) on your (data) figure 2. It is also very confusing to call the 'coral settlement modules' that you exclude when you have other 'CSMs' that you count/include. Additionally you switch back to present tense here. (note that the structure labels are again different to what is on Fig 10).

912 – this bit was already addressed elsewhere.
936 – again, repetitive.
972 -now called 'volcano' microhabitats? Yet a new term…

Reviewer #2

(Remarks to the Author)
This study evaluates the combined effects of acoustic enrichment, custom-designed artificial reef structures, and a living microbial bio-coating (Brink) on enhancing in situ coral larval settlement in Kāneʻohe Bay, Hawaiʻi. Over a two-week field experiment, the authors aimed to demonstrate that proximity to reef sound playback, presence of structural complexity, and application of Brink significantly increase coral settlement densities, highlighting the potential of integrated ecological engineering for reef restoration.

While the study presents an innovative combination of acoustic enrichment, engineered habitats, and microbial coatings to enhance coral larval settlement, I have two major methodological concerns that fundamentally compromise the validity of its conclusions.

One major overarching concern with this study is the issue of pseudoreplication, particularly regarding the experimental design for acoustic enrichment. The use of a single underwater speaker to broadcast reef sounds means that all experimental units (settlement structures) exposed to the acoustic treatment are nested within a single sound field and reef system. Consequently, the effects attributed to acoustic enrichment may be confounded with site-specific factors or unmeasured environmental variables unique to that particular location or playback configuration. Furthermore, only one source reef was used (although over multiple recording days). Without replication of the sound treatment across multiple independent reefs and sound sources, it is not possible to disentangle the effect of acoustic enrichment from potential spatial or environmental covariates.
At this stage, the conclusions drawn regarding the effectiveness of acoustic enrichment on coral settlement cannot be considered robust or generalisable. I recommend that the authors clearly acknowledge this limitation and reframe their interpretations accordingly. To substantiate these findings, replication of the acoustic treatment across multiple independent sites is essential in future work.

A second major concern relates to the acoustic characterization and assumptions about larval hearing. The study aims to enhance coral settlement by broadcasting reef sound, yet it overlooks a critical aspect of underwater hearing: reef larvae, including coral larvae, are not known to detect sound pressure directly, but rather respond to particle motion, which is suggested as the only relevant acoustic cue for these organisms in water. In shallow marine environments, the relationship between sound pressure and particle motion is complex, highly variable, and strongly influenced by local bathymetry and substrate characteristics. Therefore, the calibration and modeling of sound pressure levels and propagation loss presented

in the manuscript do not accurately inform how the larvae perceive or respond to the playback signal. Without direct measurements of particle motion at relevant positions, the study lacks key information needed to support its central hypothesis. I strongly encourage the authors to acknowledge this gap and the limitations it imposes on interpreting the biological relevance of the acoustic stimulus. Future experiments should incorporate particle motion measurements to properly characterise the acoustic environment from the perspective of the larvae.

In light of these two major issues, I must recommend rejection of the manuscript in its current form. Substantial redesign, including true replication and biologically relevant acoustic measurements, would be necessary for these findings to be considered reliable and generalisable.

Some other, more minor comments at this stages are listed below to improve the manuscript:

Abstract L. 60: Consider not using abbreviations in abstract (MH)

Introduction:
L. 190 – 212: this first part of the paragraph seems to be a repetition of L. 174 – 183
L. 216: what is the difference between micro and mesoscale habitats?

Materials:
L. 500: what do you mean with 'surface expression'?
L. 505: include more specifications for the speaker (frequency range)
L. 626 – 633: I am a bit confused with this approach. Why using artificial sound (i.e. a 'song'? and tones) to calibrate transmission loss, rather than the playback tracks themselves.
Also, why integrating between 1 – 15kHz, while the most interesting component for coral reef larvae would be well below 1 kHz? Low frequency tones may have been more appropriate to use to estimate transmission loss, as these are the relevant frequencies, and these would travel further from the speaker than the used frequencies.

3.2.2 Recordings:
- How was the recorded reef deemed 'healthy'?
- While 8 days centered around the full moon phase were recorded on that reef, I believe that only one specific location was recorded to build playback tracks. How do you address the problem of pseudoreplication?

3.2.3 Playback schedule:
- Explain your rationale for only playing back at night time

L. 708: I don't think you can call 20 – 25 kHz as 'ultrasonic'
L. 726: what is 'AE' signal?
Figure 8: Again, it would be more useful to plot frequencies below 1 kHz as these would be relevant for coral reef larvae.
Figure 8: colour bar label and unit missing
Figure 9: colour bar + units missing, and axes fonts are very small to read

Version 1:

Reviewer comments:

Reviewer #2

(Remarks to the Author)
While the authors have revised the manuscript and clarified several aspects of the study, the major methodological concerns raised in the initial review remain insufficiently resolved. In particular, the issues of pseudoreplication and the lack of biologically relevant acoustic characterisation continue to limit the strength of the conclusions that can be drawn from this work, in my opinion.

Although the authors acknowledge the limitation of conducting a single field experiment, the fundamental issue remains that the acoustic treatment itself was not replicated, which they do not discuss in the manuscript. All settlement structures exposed to acoustic enrichment remain nested within a single sound field generated by one speaker. As a result, settlement structures cannot be considered independent replicates of the acoustic treatment.

While the authors argue that spatial spread of structures reduces this concern, this does not address the core statistical issue that the treatment unit is the playback source, not the individual settlement structures. Therefore, the study still cannot robustly separate acoustic effects from site-specific environmental factors.

Coral reef soundscapes are highly heterogeneous at very small spatial scales, and individual soniferous species can dominate local acoustic conditions. For example, territorial damselfish (e.g. Stegastes) can produce intense and frequent acoustic signals and strongly influence local soundscapes around their nests. If such a microhabitat feature was present near the original recording location, the playback may unintentionally reproduce a very specific ecological context rather than a general "healthy reef" soundscape. This is just an example. Using a single recording also prevents distinguishing

biological effects from potential artefacts introduced by the recording or playback chain (e.g. recorder noise floor, spectral bias, distortion, looping artefacts, or speaker response). Replication of playback tracks is therefore not about optimising which sounds attract larvae, but about ensuring that the observed effect is attributable to acoustic enrichment as a general mechanism rather than to idiosyncratic properties of a single recording. Replication using independent recordings is standard practice in acoustic ecology specifically to avoid confounding treatment effects with recording-specific artefacts.

I also disagree with the authors' interpretation that the broadband nature of reef soundscapes justifies focusing on frequencies above 1 kHz. The biological relevance of acoustic signals should be evaluated based on the sensory capabilities of the receiving organisms rather than the spectral dominance of environmental sound.

Current evidence suggests that coral larvae, like many marine invertebrates, are likely sensitive primarily to low-frequency particle motion rather than high-frequency sound pressure. Therefore, the presence of substantial acoustic energy at higher frequencies (e.g. crustacean snaps) does not necessarily imply biological relevance for coral larvae settlement behaviour.

Furthermore, without measurements of particle motion, it remains unclear whether the biologically relevant components of the acoustic stimulus were properly characterised. As a result, the ecological interpretation of the acoustic treatment remains uncertain. I remain unconvinced by the authors' response regarding the lack of particle motion measurements. While the authors argue that particle velocity is proportional to sound pressure based on modelling, best-practice guidance (see Nedelec et al. 2021) indicates that such assumptions only hold under simplified acoustic conditions such as plane waves or ideal monopole sources. In shallow coastal environments with boundaries (ie typical coral reef) and complex sound fields (underwater speaker is not a monopole), particle motion should ideally be measured directly or estimated from pressure gradient method rather than inferred from a single pressure measurement (note that the authors did not even tried to convert their pressure measurements into particle motion in their revision).

Furthermore, best-practice recommendations emphasise that particle motion measurements are particularly important in biological studies involving fishes and invertebrates, whose hearing is often mediated by particle motion rather than sound pressure. Therefore, relying solely on pressure measurements limits interpretation of the biological relevance of the acoustic stimulus.

The authors themselves describe the study as preliminary in their rebuttal. While preliminary field trials can be very valuable, the current experimental limitations mean that the study does not yet provide sufficiently robust or generalisable evidence for the broad conclusions presented. This level of inference may be more appropriate for a specialised or exploratory journal rather than a high-impact general biology journal.

Because these concerns affect the fundamental interpretation of the results rather than presentation or clarity, I do not believe the manuscript currently provides sufficiently robust evidence to support its conclusions regarding acoustic enrichment effects. Substantial experimental redesign would be required to address these issues.

For these reasons, I cannot recommend publication in its current form.

Ref: Nedelec, S.L., Ainslie, M.A., Andersson, M.H., Cheong S-H., Halvorsen, M.B., Linné, M., Martin, B., Nöjd, A., Robinson, S., Simpson, S.D., Wang, L. and Ward, J. (2021) Best Practice Guide for Underwater Particle Motion Measurement for Biological Applications. Exeter, UK, University of Exeter for the IOGP Marine Sound and Life Joint Industry Programme, 89pp. & Appendices. DOI: http://dx.doi.org/10.25607/OBP-1726

Reviewer #3

(Remarks to the Author)
I was a new reviewer invited after the first round of review. Following the editor's request, I have focused on whether the rebuttal adequately addresses the original Reviewer #1 concerns, with particular attention to acoustic monitoring and experimental design. I have also flagged a few technical points the previous reviews did not appear to raise.

Overall assessment
The authors appear to have made a substantive revision in response to the first round comments. The most meaningful additions are the parallel fish-camera analysis, acoustic propagation modeling, an explicit treatment of pressure vs. particle motion, consistent structure nomenclature, and a reorganized Discussion where they lay clear that alternative hypotheses may explain the pattern. Most concerns from the first round of review have been addressed adequately.
Pseudoreplication and single-site design.
The authors' give a good honest response to this and acknowledge the limitations of the data they were able to collect. Reframing the settlement structure as the unit of replication is reasonable given the scale, and the convergence of distance coefficients across the two independently fit regressions (CSM br = -0.08, MCB br = -0.10) provides some indication of robustness.
However, the distance effect is driven primarily by the high settlement counts on structures close (~<6 m) to the speaker. Several structures placed at intermediate distances between 11 and 22 m show only 0-2 larvae each, meaning the data thin out considerably in the range where the regression is supposed to be resolving the shape of the decline. The exponential slope is therefore driven by the near-speaker high counts on one end and the diffuse low background beyond 21 m on the

other, with sparse and noisy data in between. The 21 m influence radius comes from the acoustic propagation model identifying 21 m as the range at which the playback sound merges into background reef noise, and not the settlement data. The settlement data are consistent with that radius, but if the acoustic model had predicted 15 m or 30 m the same settlement data would appear consistent with those values as well because the intermediate-range counts are too sparse to discriminate. For this reason I would ask the authors to add a sensitivity analysis showing how the distance coefficient behaves when the closest (≤2 m) structures are excluded. If the slope holds up without those structures, that demonstrates the gradient is a property of the broader spatial pattern rather than just driven by a handful of high-count points near the source. I think it would strengthen the case for an acoustic enrichment effect substantially. If the slope collapses, the reader and the authors both deserve to know, since it would reframe the finding as "elevated settlement close to the speaker plus a uniform low background farther away" rather than as a smooth, model-supported distance-response curve.

The Conclusion leads with the claim that all three interventions produced "statistically significant effect sizes on coral settlement, both individually and cumulatively.". This puts AE, engineered structures, and Brink coatings on equal footing but in my view the three results don't warrant equal confidence. The Brink effect is supported by a directly paired comparison: at each of several distances, MCB+ and MCB- structures were placed adjacent to one another and the coated structures consistently collected larvae while the uncoated controls collected none. That comparison is internally controlled and doesnt depend on the overall site or acoustic environment. Similarly, the structure-type comparison (CSM vs. MCB+) is supported by both structure types being present at overlapping distances and acoustic conditions, so it too is internally controlled. The acoustic enrichment result is different. It rests on a single speaker at a single location during a single spawning event, and the inference that settlement responds to AE comes from a spatial gradient relative to that one source. Within-experiment statistical significance is real and defensible, but I'm not confident that generalizable significance has been established for the AE component specifically. Irecommend softening that opening sentence with respect to AE alone, while leaving the Brink and structure-type findings to carry the stronger framing they have earned. A version that distinguishes between "robustly demonstrated" (Brink, structure type) and "suggestive and warranting follow-up" (AE) would more accurately match the strength of the underlying evidence.

Fish herbivory and fish-larvae chemical cues. The fish-camera analysis goes some way to ruling out the mature-fish herbivory mechanism. The fish-larvae signal (4-10x more larvae at the treatment site across three deployments in the companion paper) is an interesting finding in its own right. It raises a speculative question about whether fish-larvae chemical cues could contribute to the coral settlement pattern. This is outside my area of expertise and I defer to the authors on biological plausibility, but as someone outside the direct subfield the claim that the fish-larvae signal is too small or brief to have any influence on coral settlement is surprising on its face. I would suggest a sentence in the Discussion noting that an indirect pathway via fish-larvae chemistry cannot be definitively ruled out without water-chemistry sampling, while acknowledging that no specific mechanism for such a pathway appears to be well-established in the coral settlement literature (from a brief look). That would close the loop cleanly without overstating the concern.

Particle motion vs. pressure. Reviewer #2's concern was that coral larvae detect particle motion rather than pressure. The authors' response is that in the far field of a source in open water, pressure and particle motion are proportional so a pressure-based 21 m influence range translates directly to a particle-motion range. This holds cleanly in the frequency band where the playback actually carries energy above background (1-15 kHz), where wavelengths are 10-150 cm and are small relative to the 5 m water depth. The proportionality would break down at lower frequencies (e.g., 500 Hz, where ~3 m wavelength makes the whole water column "near a boundary"), but the playback carries little energy below 1 kHz, so this regime is not directly relevant to the experiment. The authors also cite Jones et al. (2025) as empirical support and explicitly flag the absence of direct particle-motion measurement as a limitation. I find this adequately addresses the concern. Reviewer #2 may prefer direct measurement in future work, but for this study the pressure-based characterization is well-matched to the playback content.

Frequency range. This appears adequately resolved, though I don't have the expertise or time to assess this fully.

Paper organization. There have been a number of changes to improve readability: acronym list, consistent nomenclature, consolidated Figure 1, and Appendix A. I found the manuscript readable.

Additional points

MCB+ vs. MCB- coefficient interpretation. Every MCB- has zero settlement at every distance. The Brink coefficient is e^7.04 ≈ 1100 with a 95% CI of [1.97, 21.85] in log units. This is a saturation artifact of regressing against an all-zero comparison group. The multiplicative interpretation is essentially meaningless. I think it's defensible to use the posteriors to claim a directional effect, but wanted to flag that it's hard to interpret the scale.

Transect XX vs. YY systematic difference. The regression finds settlement on Transect XX is ~2.5 times higher than on YY. The authors use a categorical TRANSECT term that shifts the curve up or down without changing its slope. The authors treat this as a replicate effect, but the two transects aren't really equivalent replicates. Transect XX runs parallel to the reef, so all CSMs along it sit at roughly the same (relatively close) distance from natural coral. Transect YY points away from the reef into open sandy bottom, so CSMs along YY must get progressively farther from any natural reef as they get farther from the speaker. Proximity to the reef is itself a candidate covariate for larval supply and for chemical or acoustic cues from natural coral, and the two transects differ systematically on that.

This is a plausible mechanistic explanation for the intercept difference the TRANSECT term currently absorbs: XX gets a ~constant boost from reef proximity, YY doesn't. But if reef proximity is taken seriously, it may also bias the two slopes in opposite directions. Along XX, reef proximity is roughly constant with distance from the speaker, so the XX slope should reflect AE relatively cleanly. Along YY, reef proximity declines with distance from the speaker (the two are correlated), so the YY slope mixes a declining AE effect with a declining reef-proximity effect and may overstate the AE distance dependence. I don't think this damages the findings, but wanted to highlight it and recommend the authors mention this as a potential confound and consider this for future work.

Minor

~L568 "provided permitted" - check you intended both words.

~L626 "arose due from."
A final pass would catch these and a handful of similar issues.
Recommendation
Overall I find this a strong revision that warrants publication, conditional on some minor requests. The most important requests are (1) a sensitivity analysis on the distance coefficient with the closest (≤2 m) structures excluded, and (2) softening the Conclusion's opening sentence with respect to AE specifically, to distinguish robustly demonstrated findings (Brink, structure type) from the less strongly supported AE result. I also think it's reasonable to ask for brief acknowledgments in the Discussion of the Transect XX/YY proximity-to-reef confound and a final copyedit pass. None of these requires substantial re-analysis. The Brink and structure-type findings are well-supported; the AE finding is suggestive and, if appropriately scoped, is a meaningful contribution.

Version 2:

Reviewer comments:

Reviewer #3

(Remarks to the Author)
I thank the authors for a careful and responsive revision. My main concern in the first round was the AE result being presented with the same confidence as the Brink and structure-type results despite resting on a single source at a single site, and also that the distance gradient might be an artifact of a few near-speaker high counts. Both concerns have been addressed: the sensitivity analysis I requested was added, and the authors have reframed the AE result as suggestive in the Abstract, Discussion, and Conclusion.
I recommend minor revisions for a small number of items I have suggested below. None require new data or new analysis.

1 Sensitivity analysis / pseudoreplication.
The author's language frames their findings reasonably and this has been resolved as best it can be given the limitations of the data. Table 4 shows the distance coefficient is not an artifact of the ≤2 m structures, and it remains significant with everything inside 3 m excluded.
However there is an issue with Table 4. The final row of Table 4 states "All large counts (>8) removed," but this doesn't match their raw data in Table 2 and I would ask the authors to clarify. The table reports n = 14 for this roq, i.e. 5 of the 19 removed, but from Table 2 only three structures have counts above 8 (K = 22, I = 19, N = 19; M is exactly 8), so a ">8 removed" should have n = 16. I think the five-structure set that yields n = 14 is the one named in the text at L607: {K, L, M, N, and I}. This corresponds to the ≤3 m samples plus I. But this is clearly not CSMs that have been selected based on >8. The labeling should be made consistent to clarify why this set of recorders was chosen.

2 Framing of the Conclusion. Resolved. The revised opening separates the two robustly supported findings (Brink, structure type) from the AE distance effect. This is the correct resolution to the concern raised across reviews. The issue can't be resolved with this dataset, but the results are suggestive.

3 Fish-larvae chemical cue. Resolved.

4 Particle motion, frequency, organization. Resolved.
One small thing the authors might want to clarify for readers in Section 5.2.4. First, the 500 Hz–1 kHz band is described as high-SNR. Then the pressure-to-particle-motion proportionality is said to hold more than a few wavelengths from the source, which is what lets the 21 m range apply to particle motion as well as pressure. A reader might notice that at 500 Hz the wavelength is about 3 m in only 5 m of water, so the proportionality weakens there. I don't think this is a contradiction: if the 500 Hz–1 kHz band, though high-SNR, is low in absolute energy, the range is really set by the louder 1–15 kHz band, where the proportionality holds cleanly. The authors may wish to word this section so that the "high SNR but low absolute level" point is clear - it confused me.

7 Brink coefficient. Resolved.

8 Transect XX vs YY. Resolved.

9 Minor corrections. A few typos and grammatical errors remain e.g.
L696 "would seems to provide"
L698 "definitely" → "definitively"
L553 "dissapeared." → disappeared

One more proofreading pass should catch these.

Recommendation: minor revisions. The remaining items are editorial or one-sentence clarifications; the concerns from my first review have been addressed.

Open Access This Peer Review File is licensed under a Creative Commons Attribution 4.0 International License, which permits use, sharing, adaptation, distribution and reproduction in any medium or format, as long as you give appropriate credit to the original author(s) and the source, provide a link to the Creative Commons license, and indicate if changes were made.
In cases where reviewers are anonymous, credit should be given to 'Anonymous Referee' and the source.
The images or other third party material in this Peer Review File are included in the article's Creative Commons license, unless indicated otherwise in a credit line to the material. If material is not included in the article's Creative Commons license and your intended use is not permitted by statutory regulation or exceeds the permitted use, you will need to obtain permission directly from the copyright holder.
To view a copy of this license, visit https://creativecommons.org/licenses/by/4.0/

Rebuttal letter for "In-situ enhancement of coral larval settlement using acoustic enrichment, engineered habitats and living materials"

Dear Dr. Repetto:

Thank you for your patience with this resubmission and for the opportunity to address the reviewers' feedback. We apologize for the delay; we had additional data on fish presence that directly addressed several reviewer comments, and we needed time to analyze these data and prepare a second manuscript, which is now in revision with *Scientific Reports*.

Both reviewers had detailed and productive comments, and we reproduce most of them in *italics* below. Some minor comments regarding formatting or word choice have not been reproduced; if a particular comment is missing it means we have accepted the reviewer's suggestion. Any quotations from the revised manuscript are provided in ***bold italic*** style, and any page numbers, figure numbers and paragraphs described in our response refer to the revised (not the original) manuscript.

Both reviewers often made similar and/or repetitious points, so to avoid redundancy I've divided this rebuttal into two parts. The first part provides general responses to what we believe are the five fundamental concerns the reviewers raise about this manuscript, using a couple of representative reviewer comments to highlight the issue. Following that, the next part reproduces the complete reviewer comments (excepting minor comments on formatting that we have implicitly addressed), and we cross-reference our responses to the five general responses as needed.

General responses

Here we address five major concerns of the reviewers that emerge repeatedly in their comments: (1) The issue of limited field trials; (2) the possibility of fish herbivory skewing the observed results; (3) frequency range of playback; (4) the lack of measurements of particle velocity as well as acoustic pressure; and (5) paper organization.

1. Potential for Pseudo-replication

Both reviewers expressed concern that we are presenting the results of a single field experiment, best summarized by your editorial request and Reviewer 2:

Editor: *"Specifically, Reviewer #2 raises a concern about pseudoreplication which should be addressed. Additionally, we recommend more explicitly discussing limitations and toning down some conclusions in accordance with reviewer feedback."*

Example Reviewer #2 comment:
-*"The use of a single underwater speaker to broadcast reef sounds means that all experimental units (settlement structures) exposed to the acoustic treatment are nested within a single sound field and reef system. Consequently, the effects attributed to*

acoustic enrichment may be confounded with site-specific factors or unmeasured environmental variables unique to that particular location or playback configuration."
-"*To substantiate these findings, replication of the acoustic treatment across multiple independent sites is essential in future work*."

We agree we were able to deploy only one field experiment, and therefore we have revised manuscript language to emphasize that additional experiments should be conducted to verify our results over a variety of seasons and field conditions.

However, we also assert that the unusually large spatial scale of our experiment (700 square meters) addresses some key pseudoreplication concerns by effectively sampling the random variation of crucial uncontrolled factors. Specifically, for the key sources of uncontrolled variation, we believe that our independent sampling unit is the settlement structure, and <u>not</u> the acoustic playback.

When the reviewers express concern about "site-specific factors or unmeasured environmental variables," what are these specific variables? We believe the key uncontrolled site-specific variables, listed in decreasing order of importance, are as follows:

(1) Availability of larvae (spatial density of drifting larvae), or the "patchiness" of larvae distribution.
(2) Extraneous settlement cues (i.e. chemical or acoustic cues from nearby corals).
(3) Variability in ocean motion (waves and currents) hindering coral larvae settlement.
(4) Oceanographic factors that affect behavioral response (temperature, salinity).
(5) Differences in settlement response arising from seasonal or genetic factors.

We believe that Factor 1 is by far the most important uncontrolled factor, because it is highly variable over both space and time, directly translating into variability of settlement. However, by sampling over such a large area, our experiment helps control this factor by permitting each settlement structure to provide relatively uncorrelated samples of larvae presence that allow us to perform a statistical regression on the acoustic effect. Indeed, the large area sampling allowed us to estimate a baseline settlement rate (and standard deviation) at locations where the acoustic playback falls below ambient noise levels, as seen in Figure 2.

The experiment layout also effectively provided independent samples of Factor 2, as one transect of structures was deployed parallel to a fringing reef. Thus, they were equidistant from any natural coral structures that might have been generating any substantial attraction cues that could have possibly created bias. We believe this dense yet wide sampling in *space* is a much more efficient way of accounting for variability in Factors 1 or 2 than conducting a sequence of playback experiments at random reefs with only a few settlement structures scattered over a small spatial region.

The real weakness of our experiment is that a single deployment cannot address seasonal or genetic factors in organism response (Factor 5); nor can it determine whether acoustic playbacks work under less benign oceanographic conditions (waves and currents, Factor 3). However, the purpose of this study was not to optimize acoustic enrichment methods or evaluate the practicality of the approach under a wide variety of locations and conditions. Instead, this was a preliminary study to see whether acoustic enrichment on coral larvae works *at all* in field conditions, and its potential effect size when used in conjunction with other coral restoration technologies such as 3-D printed habitats optimized for settlement, and synthetic biofilms.

Some reviewer comments also claim that replicates need to be conducted using variations of our playback configuration or recordings. Once again, we believe these extra permutations are not relevant to the goal of this study, which is to show that acoustic enrichment produces a *measurable* effect, not that any specific equipment or recordings used are *optimal*.

The key lines in the revised manuscript are as follows:

(Introduction, line 212); The total area covered by the experiment was nearly 700 m^{2,} permitting the settlement structures to experience wide variations in acoustic playback level, while also accounting for uncontrolled variations in larval supply and potential fluctuations in chemical or other cues produced by the surrounding natural coral reef. One of the transects ran parallel to a fringing reef to address this latter uncontrolled factor. Over half the structures were placed in locations where no acoustic playback signal was detectable, permitting us to estimate background larval settlement rates in the experimental area.

(Discussion, line 624) Random settlement: Because only one experiment was conducted, it's possible that the observed effect arose due from a coral settlement 'hotspot' randomly occurring near the same location as the active speaker. However, the experimental design sampled a relatively large area (~700 m^2). The area beyond the estimated influence of the active speaker (distances >21 m, or 527 m^2) showed a consistent diffuse level of settlement of about 0.0025 larvae/cm^2 (Figure 2). Thus if the observed distance effect does arise from a random process, that process would need to be highly concentrated over a relatively small area less than 20 m in radius. Additional experiments with the active source placed in a different location will further reduce the likelihood of this alternative explanation.

(Discussion, line 639): We believe that a direct coral larvae behavioral response to acoustic enrichment is the most likely explanation for our observations, but further experiments are needed to eliminate the possibility of random "hotspot" settlements. Furthermore, additional experiments are also needed to better understand potential seasonal and physical oceanographic factors that may degrade responses to AE playback.

(Conclusion, line 1411):
These results arise from a single experiment, and additional experiments should be conducted to support these conclusions. However, this study monitored a much larger area (~700 m²) than previous published experiments, allowing us to account for certain uncontrolled factors like larval surface density and potential variations in chemical cues across the site. For example, 16 structures were far enough from the speaker such that they experienced no acoustic enrichment effect; therefore, we were able to measure background settlement rates that showed little variance or spatial variation.

2. <u>Possible indirect effects of fish herbivory</u>

The second major reviewer concern was that the high concentration of structures around our playback speaker might have encouraged greater fish presence, fish consumption of algae, and generation of chemical attractant cues, which in turn would have encouraged coral larvae settlement. The acoustic playbacks from the speaker might therefore not be responsible for the higher larvae settlement counts detected near the speaker.

An example of a typical comment from Reviewer 1 is as follows: "*Additionally, I found it very challenging experimentally that there were co-incident fish attraction structures, but this community was not quantified (there was some notation of that effort in the supplemental material but it was either left out, or it was in refence to the algae hypothesis). But the fish community clearly could impact the coral that they are measuring. Both in terms of attraction of coral (so a confounding effect to the sound) as well as a potential corallivore. So it's actually quite important in this case, because you are putting out structures that are to attract fish, and there is a wealth of papers on fish larvae being attracted to sound/reefs, and we know fish species can influence coral communities, through predation, nutrients, etc...¶ that fish abundances and species should be quantified and noted here, at least as a potential explanatory variable.*"

Fortunately, during the coral larvae experiment discussed in the paper, we had been running a parallel experiment to determine whether the acoustic playbacks attracted more fish and/or fish larvae. As illustrated in Figure 1 of the revised manuscript, we deployed three "Fish Habitat Modules" (FHM) around the active speaker, and an additional three FHMs around a dummy speaker placed 42 m away from the active speaker, but at the same distance from the fringing reef. We used autonomous cameras to document the number of mature and larval fish at both sites. We found no statistically significant difference in mature (grown) fish presence at both sites but did find greater numbers of fish larvae at the active speaker site. However, the absolute number of fish counted in 2024 were small at both sites, and persisted for only a few days around the new moon. The details of this analysis were complex enough that we submitted another manuscript detailing our analysis, which is now in revision with Scientific Reports. We believe these results provide strong evidence that large-scale fish herbivory and presence is higher around the acoustic speaker due to the number of structures present. A new ***Section 3.3***

and 5.7 in this revised manuscript detail the results and methods of the fish imagery analysis and also reference the in-revision manuscript (which we have also provided for convenience). The autonomous camera analysis also found no coral-eating evidence was observed in terms of CSM, nor was there any presence of coral-consuming fish (e.g parrotfish).

3. <u>Frequency of playback and propagation effects</u>

The third major reviewer concern was why the acoustic speaker focused on acoustic energy in the kHz range, when it is "known" that sounds below 1 kHz are important components of the sound, and which may propagate differently than the higher-frequency energy. Examples of such comments include

Reviewer 1: 631/2 – It is great that you measured the received levels at this distance from 1-15 kHz. But wouldn't the acoustic energy be frequency dependent? Ie some frequencies may be louder the others (i.e. is your sound treatment flat?). Also, what about frequencies below 1 kHz? Seems like those should be quantified as well. There is a wealth of data that invertebrates respond to low frequency sound. Seems like you are missing analyses of the important frequency range

Reviewer 2: Also, why integrating between 1 - 15kHz, while the most interesting component for coral reef larvae would be well below 1 kHz? Low frequency tones may have been more appropriate to use to estimate transmission loss, as these are the relevant frequencies, and these would travel further from the speaker than the used frequencies.

Our response to these concerns is that the distances involved for the acoustic playback (0 – 40 m range in 5 m deep water) were small enough that we see little frequency-dependence in the sound attenuation, an observation supported by the acoustic modeling between 100 Hz and 10 kHz (Section 5.2.4 in revised manuscript).

The reviewers also assume that the important components of the acoustic enrichment are "well below 1 kHz". This comment seems to be an assumption on the reviewer's part; the dominant sound source in our coral reef acoustic data are crustacean sounds in the kHz range, with fish sounds below 1 kHz providing only a small fraction of the acoustic energy (and time present). The coral reef ambient sound at our study site is dominated by energy greater than 1 kHz. Sound pressure levels below this frequency are generally 20 dB less intense (***Figure 8a***, revised manuscript). Regardless of these arguments, our acoustic speaker also produced significant energy between 1-3 kHz as well (***Figure 8c,*** revised manuscript).

The key lines in the revised manuscript concerning playback and propagation effects are as follows:

(Section 5.2.4, line 832):

The playback source level was then adjusted such that the root mean square (RMS) received levels, integrated between 1 and 15 kHz, would match ambient RMS levels (signal-to-noise ratio of 0 dB) at an estimated 21 m range from the speaker. We emphasized this frequency range because the dominant acoustic energy from the reef occurred over this band (Fig. 8a) due to impulsive crustacean sounds. We assumed this band would provide the best cue for larval attraction, given that lower-frequency fish sounds in the study area were far less prevalent than crustacean snaps, with one-second calls occurring only 2-3 times per minute.

Line 854: Figure 8c shows that the largest signal-to-noise ratios (SNR) occur between 5 and 10 kHz, a frequency range dominated by crustaceans. However, similar SNR values also occur at fish vocalization bands between 1 and 3 kHz, and some ultrasonic output with up to 5 dB SNR occurs between 20 and 25 kHz.

Line 864: Acoustic modeling was used to confirm the propagation range of the acoustic playback vs frequency. The flat bathymetry significantly simplified acoustic modeling of propagation loss, using the incoherent version of the ray-tracing program BELLHOP [27]. Inputs to the model include the directional properties of the Lubell speaker [25], a water depth of 5 m, and bottom properties associated with limestone (3000 m/s compressional speed; 2.4 g/cm^3 density). Although the bottom/water interface was composed of white sand, simulations using a deep sand bottom yielded higher propagation losses with range than measured in the field, suggesting that the sand is only a thin layer over a larger coral/limestone basement. Figure 9 shows a contour map of the predicted propagation loss for both 500 Hz and 10 kHz. The propagation loss varies little with depth, except for higher losses a few cm above the ocean floor. A 15-20 dB SNR measured in the data at one meter suggests that the acoustic enrichment broadcast at 15 dB SNR (Fig. 8c) would disappear into the background noise at ranges where the propagation loss was between 16 and 21 dB, which corresponds to 11-26 m range from the speaker. The 5 dB SNR of the ultrasonic component is predicted to merge into the ambient background at only 2-3 m range. Simulations at lower frequencies (100 Hz) showed similar loss characteristics as the 500 Hz results shown here.

4. <u>Particle velocity measurements</u>

Another significant reviewer concern is the argument that our experiment did not directly measure acoustic particle velocity, which may be a component of underwater sound more relevant to larval hearing, because to our best anatomical knowledge larvae have no capability of measuring acoustic pressure (e.g. Mann, 2007, in the revised references) . As Reviewer 2 wrote:

Reviewer 2: A second major concern relates to the acoustic characterization and assumptions about larval hearing. The study aims to enhance coral settlement by broadcasting reef sound, yet it overlooks a critical aspect of underwater hearing: reef larvae, including coral larvae, are not known to detect sound pressure directly, but rather

respond to particle motion, which is suggested as the only relevant acoustic cue for these organisms in water. In shallow marine environments, the relationship between sound pressure and particle motion is complex, highly variable, and strongly influenced by local bathymetry and substrate characteristics. Therefore, the calibration and modeling of sound pressure levels and propagation loss presented in the manuscript do not accurately inform how the larvae perceive or respond to the playback signal. Without direct measurements of particle motion at relevant positions, the study lacks key information needed to support its central hypothesis. I strongly encourage the authors to acknowledge this gap and the limitations it imposes on interpreting the biological relevance of the acoustic stimulus. Future experiments should incorporate particle motion measurements to properly characterise the acoustic environment from the perspective of the larvae.

Our response is that in this environment we have found that acoustic pressure is nearly proportional to particle velocity in our numerical modeling, and so the effective range of our acoustic playback remains the same, whether measured in terms of particle velocity or pressure.

More precisely, our acoustic modeling showed that particle velocity is proportional to pressure at most locations in our study area, except within a few wavelengths (1- 2 m) of the source and within a wavelength of the ocean surface (about 50 cm at 3 kHz). These results are consistent with the known physics of acoustic propagation, where particle velocity only becomes non-proportional to pressure when the acoustic wavefront is curved (i.e. close to source) or when experiencing constructive or destructive interference due to reflections close to a boundary. There is even some debate within the bioacoustics community whether the cost and difficulty of particle velocity measurements is justified for sounds propagating in the water column (vs seafloor interface and substrate, where particle velocity is often not proportional to pressure). The most recent published papers that measure both pressure and particle velocity in the field find they typically are proportional (Jone et al 2025).

The consequence for our paper is that the 21 m range of detectable speaker sound is the same for both acoustic pressure and particle velocity, and so the mechanism for sound detection for larvae (which remains unknown) is irrelevant to our conclusions.

The key lines in the revised manuscript concerning this topic are as follows:

(Section 5.2.4, Line 895): Acoustic modeling also showed that the acoustic particle velocity of the broadcast was typically proportional to the pressure field at ranges more than a few wavelengths for the source, except within a wavelength of a reflecting boundary. These conclusions are consistent with experimental guidance (Nedelec et al 2016) and recent high-quality field measurements (Jones et al 2025). Thus the 21 m range modeled here is consistent for both pressure and particle velocity, an important point given that larvae are expected to be more sensitive to particle velocity than acoustic pressure (e.g. fish larvae in (Mann et al 2007)).

(New bibliography additions):

Nedelec, S.L., Campbell, J., Radford, A.N., Simpson, S.D., Merchant, N.D.: Particle motion: the missing link in underwater acoustic ecology. Methods in Ecology and Evolution 7(7), 836–842 (2016).

Jones, I.T., Martin, S., Miksis-Olds, J.: Exploring offshore particle motion sound-scapes. The Journal of the Acoustical Society of America 157(1), 149–168 (2025).

Mann, D.A., Casper, B.M., Boyle, K.S., Tricas, T.C.: On the attraction of larval fishes to reef sounds. Marine Ecology Progress Series 338, 307–310 (2007).

5. <u>Paper Organization</u>

A final major concern is the organization of the manuscript. This is a complex experiment with a lot of components provided by multiple researchers, and we appreciate the reviewer suggestions on how to organize the manuscript to clearly delineate the various aspects of the setup. Specific steps we have taken in this revision include the following:

(1) We added a list of acronyms for the various structures and field components, which have renamed with more intuitive labels and applied consistently throughout the manuscript, including figures and figure captions.
(2) We moved material reviewing previous research in the three main attractant methods (structures, synthetic biofilms, acoustic enrichment) from the Introduction into an Appendix A.
(3) We consolidated figures and removed the original heat map figure, which the reviewers found more confusing than helpful. Figure 1 now shows the complete experimental layout with all relevant structures, with colors used to help distinguish structure types. Raw results are now also displayed in the figure.
(4) We updated references that refer to individual components of the experiment:
    a. Reichert, J., et al, "Helix recesses boost coral larvae settlement and survival. " Biological Conservation 311, 111407 (2025) https://doi. (for settlement structures)
    b. Levy, Natalie, et al. "Microbial living materials promote coral larval settlement." *PNAS nexus* 4.9 (2025): pgaf268. (for synthetic biofilms)

(5) We have used our fish photograph measurements to address questions about the potential interaction between fish and coral larvae settlement (***Sections 3.3 and 5.7)*** and relegated our direct algal measurements to an Appendix B, as we feel that the fish results better address these questions.
(6) Acoustic measurements and comprehensive modeling have been merged into a single section.

We disagree with the suggestion to break up the paper into separate mini-papers, because we feel the integration is a strength of the experiment, and the statistical design allowed us to cleanly distinguish synthetic biofilm effects from potential acoustic enrichment effects.

We do, however, have our fish presence analysis assigned to a separate paper in revision with Scientific Reports.

Detailed responses to complete reviewer comments

Original reviewer comments are in *italic;* our responses are in plain text, quotes from revised manuscript are in ***bold italic.***

Reviewer #1 (Remarks to the Author):

Review of:
In situ enhancement of coral larval settlement using acoustic enrichment, engineered habitats and living materials
Thode and colleagues.

Overall, a very interesting study.
I have quite a few points that would need attention before I can recommend publishing, especially publishing in Nature Communications.

General Points:
One is that the text is generally confusing to read.

The names of the devices/structures are a bit jargony (Brinks, CSMs, MHs, microhabitats, superdomes, mini superdome CSM) and they are used a bit interchangeably, or at least variably, making it all a bit hard to follow. And there is a decent amount of repetitive text. Perhaps this was cowritten? But the text needs a strong read/edit from just one (perhaps new) set of eyes to streamline the message.

We have added a table of acronyms to the beginning of the paper and revised the text to be consistent in the naming of structures. Please also see comments under "Section 5. Organization" in the general comments above.

Additionally, re: the multiple factors, while novel, contribute to this tangled storyline. While there is certainly potential in putting all methods together the writing needs to be improved to convey the details and nuances. Sure, it's easy to gain the topline message ,"It all works," but which devices, how and precise setup are confusing. This could be separated into multiple papers, or if the authors want to keep it all together, they (or perhaps one person) need(s) to work on the writing. Similarly, the figures are difficult to

gain meaningful information from, and at times are a bit misleading. They need substantial work noted below.

We disagree with the suggestion to break up the paper into separate mini-papers, because we feel the integration is a strength of the experiment, and the statistical design allowed us to cleanly distinguish synthetic biofilm effects from potential acoustic enrichment effects. The one exception is the fish presence analysis, which has been submitted as a separate manuscript, with summaries provided in the current manuscript.

For more responses on paper organization, please see General Comments, Section 5, "Paper Organization". Figures have been consolidated and redesigned.

Additionally, I found it very challenging experimentally that there were co-incident fish attraction structures, but this community was not quantified (there was some notation of that effort in the supplemental material, but it was either left out, or it was in refence to the algae hypothesis). But the fish community clearly could impact the coral that they are measuring. Both in terms of attraction of coral (so a confounding effect to the sound) as well as a potential corallivore. So it's actually quite important in this case, because you are putting out structures that are to attract fish, and there is a wealth of papers on fish larvae being attracted to sound/reefs, and we know fish species can influence coral communities, through predation, nutrients, etc... that fish abundances and species should be quantified and noted here, at least as a potential explanatory variable.

Please see General Comments, Section 2, "Possible indirect effects of fish herbivory."

Overall, the figures and story need to be consolidated. As it reads now, it is just too complicated (or maybe disparate) to figure out what is going on. The overall "story" of integration is a good idea but it is not working as it is written. This is further confused by the authors varying how the refer to the various settlement structures (which in a way is the new ideas). The authors either need to separate into multiple papers (which may be impossible given the experimental design) or re-write and redesign the paper. But separating, if possible, several smaller papers would help streamline each individual message. Otherwise, this paper needs a lot of restructuring.

Please see General Comments, Section 5, "Paper Organization".

Additionally: The discussion of direct/indirect algal effects is interesting but feels very difficult to get at. They mention that acoustic enrichment could attract more herbivores and make settlement surfaces more attractive, but you could also attract corallivores, leading to more direct post-settlement mortality... It seems like something the authors felt (or were told) to address but it is so different from the rest of the paper/not really substantiated in a meaningful way (to support the hypothesis).

This comment concerns an analysis that has been superseded by analysis of fish presence using autonomous cameras. Please see General Comments, Section 2, "Possible indirect effects of fish herbivory."

Finally, one of the challenges of the experiment is that it was setup as both a dose- based and a paired treatment vs. no treatment (sound vs. no sound; brink vs. no brink) and given the variability (0-22 in some cases) it is really really hard to discriminate impact. For example, if you test between 1 m CSM at sound vs. no sound, there's no effect (t test). But agreed, 1 m brink vs. not brink does show impact-increased settlement create (similarly overall brink vs not-brink shows a positive effect). But basically 3 to 4 points drives the distance/dose regression. Without those points (and still including data at 1 m) the regression is basically flat. So while there is a dose-based effect it is not a very strong signal at all (at least acoustically). I am wondering whether the sound is confusing things. One thing that might put this variable settlement into context is to address natural settlement expected in Kaneohe Bay or natural Hawaii settlement overall (although I'd guess the Bay would be quite different than ocean coastline).
Overall, I would suggest the authors carry out some separate focused experiments that address these parameters individually. And then focus on the design with more replication to better discern patterns.

Settlement was observed on multiple structures (both CSM and MCB) at distances greater than 1 m, and the resulting coefficients in our statistical regression were significantly different from zero. Furthermore, independent regressions on two different structures (the CSM and MCB) yielded virtually the same regression coefficient values.

The large numbers of structures with little to no settlement were part of the experimental design in that they allowed us to estimate natural background settlement levels in the sandy area in the absence of acoustic enrichment. Please also see General Comments, Section 1, "Potential for Pseudo-Replication."

Figure needs:
For example, Fig 1, yes while larval settlement / m2 is technically correct the numbers measured were far lower (so this is scaling up/estimating what the numbers would be if they were measuring a meter squared). Hence a bit disingenuous. Per cm^2 would be more reflective of the reality of the experiment.

We had used counts per square meter to be consistent with MKS standards. However, we have now converted all results in terms of counts per square centimeter.

Fig 2, lettering and symbols are very small making them hard to read without blowing up figure (for example, the compass has bigger letters and that's probably the least important part of the figure). Why is the title "Relative" larval counts? Isn't it actual? It is really hard to tell the symbol different between the oval of the microhabitat only and the rectangle microhabitat with (in this case it's called "bacteria reef ink (Brink),"). The inset pic is almost helpful but too small to discern actual setup. Overall results are really hard to dissent from this figure...
You should also label on the figure the YY and XX transect lines.

We have redesigned the revised Figure 1 to address all these issues. We no longer show a heat-map type figure because it was considered confusing.

Fig 3. PDD or PDF? Seems to use both. Fonts are much smaller than Fig 1 and hard to read again.

Fixed label and size inconsistencies.

Fig 4. Same comments. These could also be supplemental. They don't seem very critical to the results or methods (but support the methods ...)

Showing independent regression results for the MCB and CSM surfaces is an important part of our argument to demonstrate that acoustic enrichment creates a real effect in our analysis.

Fig 5. Similar to Fig 1. Using per meter squared seems to convey much higher settlement than actually observed. Also the filled points are actual data. Seems like the regression should not have points. Similarly (with respect to analyses) the regression slope seems basically driving by the first 1 meter settlement rates. If those are removed is the settlement basically flat? This would seem to indicate that that there is very little (or no effect) beyond 1 m. CSM is also the superdome, correct? Pls keep consistent. Further, data here should be presented as CSM Transect YY model (vs. "measured,")

Settlement was observed on multiple structures (both CSM and MCB) at distances greater than 1 m, and the resulting coefficients in our statistical regression were significantly different from zero. Furthermore, independent regressions on two different structures (the CSM and MCB) yielded virtually the same regression coefficient values.

The large numbers of structures with little to no settlement were part of the experimental design in that they allowed us to estimate natural background settlement levels in the sandy area in the absence of acoustic enrichment. Please also see General Comments, Section 1, "Potential for Pseudo-Replication."

Fig 6. Seems like this could be combined with Fig 2 or 10 as they are all setup/location based figures...¶

Some figures have been consolidated.

Fig 7. Natural reef sound (not noise). Noise has a very specific definition/connotation in and outside of the acoustics world. See Art popper's work, as well as Noise Matters by Haven Wiley.

Done.

Fig 9, again units are super small and hard to read. A scale bar would be helpful. Y axis is not labelled in A. Just some careless things here.

Fixed.

Fig 10. Can this be combined with Fig 2? Again, with all the variables it's hard to discern what actually occurred and I found myself flipping back and forth between this fig and fig 2.
You could probably just make one figure with the symbols all bigger (or put together as an a and a "b," and the actual results.

Figure 10 has been combined with Figure 2 to become Figure 1 in the revised manuscript. The "heat-map" figure has been removed.

Notably, re: results, Table 1 is quite important but it's buried (but all the figures are integrated?). Overall, I find myself looking for a figure that actually shows the results, but it's not really included. Fig 2 is almost impossible to read and shading doesn't really help as you have to go back and forth to the colour bar to figure out what colour means high vs. low numbers. Fig 5 is inflated numbers. I know it's not how you ran your stats, but just some simple bar or scatter plots.

Figure 1 in the revised manuscript now shows the raw data also displayed in Tables 1 and 2.

Fig 11 is helpful but also so different from the rest of the paper. It really reflects that the paper seems to be written and integrated by a number of authors without a true story or voice. Also note here that the names of the structures are inconsistent with how they are referred to or labelled in other parts of the paper. Alternatively, put all the two "structure" figures together (11 and 12) so that we see the settlement surfaces all in one place.

We have consolidated all structure figures into revised Figures 10 and 11.

Similarly, there is a wealth of papers on larval reef fish being attracted to sound. But this is not addressed entirely (in a paper that is otherwise seeking to be integrative). I get that the authors want to make a second paper on this but there is a relationship between fish and coral. I would think an alternative hypothesis to the coral + sound is that the authors added structures + sound that may have recruited juvenile or adult (or larval fish). See work by Tim Gordon and Steve Simpson and colleagues. And these fish are constantly producing metabolomics and nutrients and chemical cues. And these chemical cues attract the coral larvae.

See Lecchini and related work below. Some are pasted below. (I am not sure what to do with the Dixson papers).

Lecchini D, Nakamura Y. Use of chemical cues by coral reef animal larvae for habitat selection. Aquatic Biology. 2013 Oct 24;19(3):231-8.

Pawlik JR. Induction of marine invertebrate larval settlement: evidence for chemical cues. Ecological roles of marine natural products. 1992:189-236.
Slattery, Marc. "Chemical cues in marine invertebrate larval settlement." Fouling Organisms of the Indian Ocean. CRC Press, 2020. 135-157.
**Quinlan, Zachary A., et al. "Coral larval settlement induction using tissue-associated and exuded coralline algae metabolites and the identification of putative chemical cues." Proceedings of the Royal Society B 290.2009 (2023): 20231476.*
Rittschof D, Forward Jr RB, Cannon G, Welch JM, McClary Jr M, Holm ER, Clare AS, Conova S, McKelvey LM, Bryan P, Van Dover CL. Cues and context: larval responses to physical and chemical cues. Biofouling. 1998 Jan 1;12(1-3):31-44.
Gerlach G, Atema J, Kingsford MJ, Black KP, Miller-Sims V. Smelling home can prevent dispersal of reef fish larvae. Proceedings of the national academy of sciences. 2007 Jan 16;104(3):858-63.

Sweatman, Hugh. "Field evidence that settling coral reef fish larvae detect resident fishes using dissolved chemical cues." Journal of Experimental Marine Biology and Ecology 124.3 (1988): 163-174.

Please see General Comments, Section 2, "Possible indirect effects of fish herbivory." By using analysis of autonomous cameras at two locations within the study area (the subject of a second paper in revision), we have addressed these concerns directly.

We have added Lecchnini and Quninlan as references to Appendix A, with provides a literature review of larvae recruitment enhancement techniques.

Specific comments:
A small note here, the numbers on the manuscript are not actually line numbers. They don't match up with the lines of text. Making it annoyingly challenging to address specific comments. And perhaps challenging for the reviewer to convey edits to the author. This should be fixed on the next version.

The slight misalignment between line numbers and text is a consequence of using the double-spaced option in our software (Overleaf.com) to produce a reviewer-friendly manuscript. We feel that keeping the double spacing is our highly priority and feel that the line numbers are sufficiently well aligned with the text to avoid any confusion.

088/90 -This sentence, especially the first have, needs to be rewritten. It is unclear. "A key challenge remains the limited success of natural coral recovery, necessitating innovative solutions to support reef resilience."

96/98 -what debate about relative importance of natural reef restoration versus artificial reef deployment? You probably need more context or a reference here. Or maybe those are the following refs (~lines 101/102?) but are those refs ," debating," or just offering alternative ideas. In either case this point does not seem very directly relevant to the paper. Why not just talk about the importance of supporting/restoring reefs in peril?

The introduction has been revised to incorporate these suggestions.

130 -Refs 25,26 seem incorrect. 26 is not coral larvae. 25 is a field experiment.

References have been reviewed and corrected. The entire section has been moved to Appendix A.

164 -I believe the authors mean to suggest "sub-metre," scale. Metre seems too large.

Done.

167 -oddly this Reichart bioarxiv paper is not numbered. Further, it is not peer reviewed. I think generally all journals ask for peer review references. Certainly Nature. Pls remove.

187 -46 is similarly a bioarxiv un peer reviewed paper. Basically a webpage. This should be removed

These papers have now been published in peer-reviewed journals and are now cited correctly. Please see General Comments, Section 5, "Paper Organization".

192 -This paragraph is confusing. Are the authors suggesting biohybrid substrates are a 4th"emerging approach for enhancing coral recruitment,"? or is this a continuation of the thoughts of the previous paragraph. Sorry, it is just unclear.

Appendix A now contains the literature overview and clarifies that biohybrid substrates are one of the three technologies being focused on by the paper.

203 -what is R3D?

Acronym removed and R3D is now defined in the acknowledgements and Introduction.

232/238 -This is all very confusing. If this is well established, in can stay in the intro but needs a reference or two about your CSMs, or move this to the methods with appropriate methodological figures to describe. Otherwise, it is not enough information and out of context. But you are basically describing methods and it is confusing to follow.

The journal requirement to put results before methods was challenging. We have rewritten the latter part of the Introduction to give a sufficient overview of the experimental layout to provide context for the Results section. A much more detailed description of the experimental structures and layouts is provided in the Methods section.

243/4 -new moon spawning of what species? Fish and coral species spawning times generally vary by species. You seem to have an idea which species you are targeting…

The revised text clarifies that the dominant coral species in Kaneohe Bay is <u>Montipora capitata.</u>

Results

[in the first half of the Results, it seems like authors do not have enough data for statistical significance...so are mostly describing trends which are not really robust for peer-reviewed publication (if you are trying to make the point of ”effect,”). There is a lot about ”notable,” and trends (”twice as high,”) but without robust stats to back up their apparent point.

281 -”The enhanced densities are notable up to 21 m range,” is this significant? Or just ”notable,”? seems really vague here. Pls clean up language.

300 -Authors switched to present tense here. Pls keep consistent. So was there a statistical difference here? Or general trends?

The text referenced by the comments above were reorganized into Section 3.1 “Raw results,” which gives a qualitative overview of the results before Section 3.2, “Statistical regression,” which provides a quantitative evaluation of the various experimental factors on coral settlement.

403 paragraph -These results are unclear. For one, I am not sure how to pull out the 2.5x greater point from Fig 3c. My instinct is to go to the nearby figure 2 which has results (but is hard to read) but YY and XX are not labelled on that figure. Nor are the site letters. Fig 5 is largely a model. So discerning what you are talking about is a bit challenging. In the following sentence, I do not think site ”I” is on the 1 m circle (as shown in Fig 10) so this sentence does not make sense. Finally, if you changed the data points, and found different results (last sentences) wouldn’t that imply that the results (differences between transects) are real?

The revised line now reads “**The CSM regression also found that settlement rates along transect XX were roughly 2.5 [$e^{0.94}$] times greater than along Transect YY**”, allowing the reader to connect the 0.94 value from Fig. 3(c) to the ratio between Transects XX and YY.

418 paragraph. Agreed, the unbalanced treatment is really unfortunate and effects the experimental design, and thus our interpretation of the results. The authors make the somewhat half developed point about algae. But we know coral larvae respond to chemical cues and structure. Thus making a design that is not balance in structure (and the cues that can then co-develop from attracted fish) really does impair how we determine the acoustic results. This is indeed unfortunate. I am not sure what to do about this. I would generally say this test should be repeated properly. At the minimum, it would be helpful to provide the fish data along with these results.

Please see General Comments, Section 2, “Possible indirect effects of fish herbivory.”

By using camera analysis at the site (the subject of a second paper in revision) we have addressed these concerns directly.

Also, note here, you are referring to the MHs as cinder blocks. This flipflopping of naming convention is very challenging to a reader to follow. Maybe call them cinder blocks+MH+Brink...¶and cinder block+MH (or some more explicit label).

Experimental structures are now labeled more intuitively and consistently.

442/3 -you note the fish data are in the SM but I did not see it there. Pls provide or clarify.

Fish data are now incorporated into the paper. Please see General Comments, Section 2, “Possible indirect effects of fish herbivory,” and the related manuscript provided with this revision.

631/2 -It is great that you measured the received levels at this distance from 1-15 kHz. But wouldnt the acoustic energy be frequency dependent? Ie some frequencies may be louder the others (ie is your sound treatment flat?). Also, what about frequencies below 1 kHz? Seems like those should be quantified as well. There is a wealth of data that invertebrates respond to low frequency sound. Seems like you are missing analyses of the important frequency range.

Please see General Comments, Section 3, “Frequency of playback and propagation effects,” for detailed discussion.

782 -the challenge here is that the ”open-hexagon CSMs, used as artificial fish habitats,” were part of this experiment... Not accounting for their potential influence to the community (given that we know fish respond to sound) seems like a real oversight and should be addressed more completely.

The open-hexagon CSMs, renamed Fish Habitat Modules (FHM) in the revised manuscript, were a key part of our analysis using autonomous cameras to evaluate whether higher levels of fish presence were associated with the active speaker location. Please see General Comments, Section 2, “Possible indirect effects of fish herbivory.”

821/8 -this 2 m ring does not seem clear (or does not appear) on your (data) figure 2. It is also very confusing to call the ,”coral settlement modules,” that you exclude when you have other ”CSMs,” that you count/include. Additionally you switch back to present tense here. (note that the structure labels are again different to what is on Fig 10).

All structures now have a consistent nomenclature, and the revised Figure 1 now clearly shows the FHM locations.

Reviewer #2 (Remarks to the Author):

This study evaluates the combined effects of acoustic enrichment, custom-designed artificial reef structures, and a living microbial bio-coating (Brink) on enhancing in situ coral larval settlement in Kane'ohe Bay, Hawai'i. Over a two-week field experiment, the authors aimed to demonstrate that proximity to reef sound playback, presence of structural complexity, and application of Brink significantly increase coral settlement densities, highlighting the potential of integrated ecological engineering for reef restoration.

While the study presents an innovative combination of acoustic enrichment, engineered habitats, and microbial coatings to enhance coral larval settlement, I have two major methodological concerns that fundamentally compromise the validity of its conclusions.

One major overarching concern with this study is the issue of pseudoreplication, particularly regarding the experimental design for acoustic enrichment. The use of a single underwater speaker to broadcast reef sounds means that all experimental units (settlement structures) exposed to the acoustic treatment are nested within a single sound field and reef system. Consequently, the effects attributed to acoustic enrichment may be confounded with site-specific factors or unmeasured environmental variables unique to that particular location or playback configuration. Furthermore, only one source reef was used (although over multiple recording days). Without replication of the sound treatment across multiple independent reefs and sound sources, it is not possible to disentangle the effect of acoustic enrichment from potential spatial or environmental covariates.
At this stage, the conclusions drawn regarding the effectiveness of acoustic enrichment on coral settlement cannot be considered robust or generalisable. I recommend that the authors clearly acknowledge this limitation and reframe their interpretations accordingly. To substantiate these findings, replication of the acoustic treatment across multiple independent sites is essential in future work.

Please see General Comments, Section 1, "Potential for Psuedo-replication," for relevant discussion of these concerns.

A second major concern relates to the acoustic characterization and assumptions about larval hearing. The study aims to enhance coral settlement by broadcasting reef sound, yet it overlooks a critical aspect of underwater hearing: reef larvae, including coral larvae, are not known to detect sound pressure directly, but rather respond to particle motion, which is suggested as the only relevant acoustic cue for these organisms in water. In shallow marine environments, the relationship between sound pressure and particle motion is complex, highly variable, and strongly influenced by local bathymetry and substrate characteristics. Therefore, the calibration and modeling of sound pressure levels and propagation loss presented in the manuscript do not accurately inform how the larvae perceive or respond to the playback signal. Without direct measurements of particle motion at relevant positions, the study lacks key information needed to support its central hypothesis. I strongly encourage the authors to acknowledge this gap and the limitations it imposes on interpreting the biological relevance of the acoustic stimulus.

Future experiments should incorporate particle motion measurements to properly characterise the acoustic environment from the perspective of the larvae.

Please see General Comments, Section 4, "Particle velocity measurements," for relevant discussion of these concerns.

Introduction:
L. 190 -212: this first part of the paragraph seems to be a repetition of L. 174 -183.
L. 216: what is the difference between micro and mesoscale habitats?

Text has been revised and/or removed.

Materials:
L. 500: what do you mean with "surface expression,"?

Revised to "surface buoy".

L. 505: include more specifications for the speaker (frequency range)

Section 5.2.1 now contains these details.

L. 626 -633: I am a bit confused with this approach. Why using artificial sound (i.e. a "song,"? and tones) to calibrate transmission loss, rather than the playback tracks themselves.
Also, why integrating between 1 -15kHz, while the most interesting component for coral reef larvae would be well below 1 kHz? Low frequency tones may have been more appropriate to use to estimate transmission loss, as these are the relevant frequencies, and these would travel further from the speaker than the used frequencies.

The actual playback tracks contained mostly pulsive sounds; tones and song provided a better means to characterize potential frequency-dependent effects in the playback. Playing back reef noise in the presence of other reef noise would have required application of a matched filter to identify the sound sequence. If the broadcast signals cover the same bandwidth as the reef playback, any derived propagation loss is the same.

Please also see General Comments, Section 3, "Frequency of playback and propagation effects," for relevant discussion of these concerns.

3.2.2 Recordings:
- How was the recorded reef deemed "healthy,"?

One of our co-authors, Dan Schar, has snorkeled this area for years and noted it was an area of high fish diversity and corals that showed no signs of bleaching or otherwise ill health.

- While 8 days centered around the full moon phase were recorded on that reef, I believe that only one specific location was recorded to build playback tracks. How do you address the problem of pseudoreplication?

Please see General Comments, Section 1, "Potential for Psuedo-replication," for relevant discussion of these concerns, where we state our opinion that the details of the recording are irrelevant to concerns about pseudoreplication.

3.2.3 Playback schedule:
- Explain your rationale for only playing back at night time

Coral and fish recruitment in this area takes place during the night, and previous acoustic enrichment literature has focused on nighttime playbacks. For example (Gordon et al 2019) conducted their playbacks at night.

L. 708: I don't think you can call 20 -25 kHz as "ultrasonic,"

Frequencies about 20 kHz are defined as "ultrasonic" in the literature because they are beyond the range of most human hearing. (https://www.merriam-webster.com/dictionary/ultrasonic)

Figure 8: Again, it would be more useful to plot frequencies below 1 kHz as these would be relevant for coral reef larvae.

We have now plotted 500 Hz instead of 1 kHz in Figure 9 (revised). No effective difference in propagation loss is observed, as discussed in General Comments, Section 3, "Frequency of playback and propagation effects".

On behalf of all co-authors, I sincerely appreciate the feedback provided thus far from reviewers and the Editor.

Sincerely,
Aaron Thode and Co-Authors

Rebuttal letter for "In-situ enhancement of coral larval settlement using acoustic enrichment, engineered habitats and living materials"

Dear Dr. Repetto:

Thank you for your patience with this resubmission and for the opportunity to address the reviewers' feedback.

Both reviewers had detailed and productive comments, and we reproduce most of them in *italics* below. Some minor comments regarding formatting or word choice have not been reproduced; if a particular comment is missing it means we have accepted the reviewer's grammatical suggestion. Any quotations from the revised manuscript are provided in **bold** style, and any page numbers, figure numbers and paragraphs described in our response refer to the revised (not the original) manuscript.

In essence, we have addressed and incorporated both your and Reviewer 3's recommendations. In terms of Reviewer 2, while we disagree with some of the comments, we feel the most productive response was to adjust the Discussion and Conclusion to emphasize the tentative nature of the acoustic enrichment results, and to recommend how future experiments can be improved. We have also included a PDF with changes highlighted.

Editor comments

1. Sensitivity analysis of AE effect

-Editor 1: *Include a sensitivity analysis of the distance effect to assess the robustness of the observed pattern, as suggested by Reviewer #3.*

Reviewer #3: "For this reason I would ask the authors to add a sensitivity analysis showing how the distance coefficient behaves when the closest (≤2 m) structures are excluded. If the slope holds up without those structures, that demonstrates the gradient is a property of the broader spatial pattern rather than just driven by a handful of high-count points near the source. I think it would strengthen the case for an acoustic enrichment effect substantially. If the slope collapses, the reader and the authors both deserve to know, since it would reframe the finding as "elevated settlement close to the speaker plus a uniform low background farther away" rather than as a smooth, model-supported distance-response curve."

We agree this is a good suggestion and reran the analysis while removing various CSM samples, to see whether the counts at < 2 m range dominated the resulting exponential fit. The new Table 4 in the manuscript (reproduced below) summarizes the distribution of the distance coefficient (e.g. the variable plotted in Figure 3a) when various CSM samples are excluded. We also reproduce the existing Table 2 for convenience.

Table 4: Sensitivity of CSM distance coefficient $b_{r,CSM}$ to various data points in Table 2.

Description	Samples	Estimate	Error	Lower 95% CI	Upper 95% CI
All samples	19	-0.08	0.01	-0.10	-0.06
Remove all samples <= 2 m range	16	-0.11	0.02	-0.15	-0.07
Remove all samples <= 3 m range	15	-0.09	0.02	-0.13	-0.05
Remove CSM I only	18	-0.08	0.01	-0.11	-0.06
All large counts (>8) removed	14	-0.02	0.03	-0.07	0.04

Table 2: Settlement results on CSM surfaces measured on July 18th, expressed in terms of raw counts and settlement density. The distance column shows the distance of CSMs from playback speaker, and the last column indicates the fraction of CSMs covered by algae during an August 20th survey.

CSM	Transect	July 18 Raw counts	July 18 counts/m^2	Distance (m)	Algae August 20 Mean fraction
A	XX	2	24.5	41.8	0.92
B	XX	1	12.2	40.8	0.98
C	XX	0	0.0	40.8	0.95
D	XX	3	36.7	39.9	N/A
E	XX	1	12.2	34.4	0.93
F	XX	2	24.5	28.7	0.97
G	XX	1	12.2	22.9	0.97
H	XX	5	61.2	17.1	N/A
I	XX	19	232.6	11.6	1.00
J	XX	0	0.0	5.8	0.92
K	XX	22	269.4	0.9	0.95
L	XX	0	0.0	0.9	0.15
M	YY	8	98.0	0.9	0.60
N	YY	19	232.6	3.0	0.93
O	YY	1	12.2	5.8	1.00
P	YY	0	0.0	8.2	0.95
Q	YY	2	24.5	11.6	0.87
R	YY	0	0.0	17.1	0.90
S	YY	2	24.5	22.9	0.63

We find that we need to remove 5 samples, including the samples from CSM I (11.6 m range) and N (3 m range), to get a coefficient estimate whose 95% confidence interval includes zero (not a significant effect). The reviewers tend to focus on the samples just two meters away from the speaker, but it turns out that Samples I and N are also key data points in the regression. For example, even if all samples less than 4 m range are removed from the regression (Row 3 in the table below) the resulting regression coefficients are still numerically similar to the situation where all data points are retained, due to the results on CSM I. Other diagnostic metrics from the regressions look healthy; R_hat was always 1, indicating good mixing from the Bayesian inversion, and the Bulk ESS was always greater than 3000, indicating samples were sufficiently uncorrelated to permit a reasonable regression.

We have added this sensitivity analysis to the Discussion of the revised manuscript (Lines 599-617) along with a new Table 4 (Lines 2642-2649).

2. <u>Other editor comments</u>

Editor 2: Refine the framing of the acoustic enrichment (AE) result, particularly in the Conclusions, to better distinguish between findings that are strongly supported within the current design and those that are more preliminary.

We have revised the Abstract, Discussion and Conclusion to reframe the AE result. Specifically, the start of the Discussion (567-583) now separates the strong conclusions of the Brink and settlement surface results from results associated with the speaker distance effect (585-617). A new subsection 4.1 "Interpretation of the speaker distance effect" (lines 637-722) clarifies that while the distance effect observed is real, interpreting the result as AE is preliminary. The revised Conclusion (1533-1585) also reframes the AE results separately from the other technologies and clarifies which findings are strongly supported vs. preliminary. For convenience, the key lines from the Conclusion are reproduced below:

"An integrated field test of three techniques for boosting coral larval settlement during natural spawning found robust and statistically significant effect sizes on coral larvae settlement for two of them: synthetic "Brink" biofilms, and customized settlement structures. Synthetic biofilm-coated surfaces (MCB+) resulted in coral settlement, while corresponding control surfaces (MCB-) showed no settlement at all. CSM surfaces displayed higher settlement densities than MCB. For practical applications, these surfaces are not exclusive, and a scaled-up CSM design is currently incorporating smaller MCB+ structures at spots along its surface to leverage the settlement enhancing benefits of both techniques.

"This study also identified a statistically significant effect of settlement with respect to distance from an acoustic enrichment speaker. However, since these results arose from a single deployment using a single speaker, alternative explanations for the observed distance effect remain viable. These explanations include the possibility of a random settlement hotspot that may be generated by natural chemical or acoustic cues released from natural reef structures that happened to coincide with the placement of the acoustic speaker. Another alternative explanation is that fish herbivory was greater near the acoustic speaker, due to the high concentration of structures there. As discussed in Section \ref{sec: Discussion and Interp}, evidence against this latter explanation was provided by autonomous cameras that showed that no significant difference in fish presence at 1 m or 42 m away from the speaker, although there was a difference in fish larvae presence near the speaker for a few days surrounding the full moon. Full details of that analysis are provided in Ref. [18].

A single AE deployment also cannot address seasonal or genetic variability in potential organism responses; nor can it determine whether acoustic enrichment would work under less benign oceanographic conditions with higher currents and/or wave action. Therefore, additional acoustic enrichment experiments, with a speaker in different locations, would be required to reduce the possibility of the observed distance effects being the result of a random settlement hotspot, and to demonstrate replication across a wider variety of locations that span different ocean conditions and reef environments. Acoustic recordings from other locations should also be randomly seeded in the replicate deployments to confirm that any attractant effect arises from coral reef ambient sound and not playback artifacts arises from a particular recording."

Editor 3: Expand the discussion of limitations, including the single-site/single-source design,

This is addressed in revised lines 1568-1581.

Editor 4: the chosen frequency range,

Section 5.2.4 (Playback field measurements) has expanded the description of the playback and ambient sound frequency range, placing it in context with previous studies. We have added a new subsection 4.2 in the Discussion ("Low vs. high-frequency components of playback")

Lines 947-994 of Section 5.2.4 now reads as follows:

The playback source level was then adjusted such that the root mean square (RMS) received levels, integrated between 1 and 15 kHz, would match ambient RMS levels (signal-to-noise ratio of 0 dB) at an estimated 21 m range from the speaker. The dominant acoustic energy from the reef occurred over the 1-15 kHz band (Fig. 8a) due to impulsive crustacean sounds. However, the playback also included significant energy between 500 Hz and 1 kHz, a band typically associated with fish sounds and dubbed the 'low-frequency range' in previous coral reef acoustic literature [25,26].

During the full experiment on July 2024 the same SoundTrap was placed 1 m away from the speaker to independently verify playback levels. Figures 7a and 7b show spectrograms of the sound field a few minutes before and after playback began on July 5th, the evening of the new moon. Figure 8 shows the measured percentile spectra of both the ambient (Subplot a) and playback sound expressed in terms of 1 m range from the speaker (Subplot b), with units expressed as power spectral density (PSD). The percentile curves span 30 dB, indicating the high impulsive content of both the ambient sound and playback data. Both the ambient and playback sounds are dominated by crustacean snaps over a frequency band between 2 and 15 kHz. Sounds below 1 kHz are 5-10 dB lower than the crustacean band. The high intensities visible below 100 Hz seem to arise from flow and other non-acoustic artifacts; manual reviews of fish sounds in the recordings find little energy below 100 Hz.

Figure 8c shows that the largest signal-to-noise ratios (SNR) of the playbacks, relative to ambient sound, are between 15 and 20 dB, occurring between 500 Hz and 1 kHz and between 5 and 10 kHz. Thus frequency bands associated with both fish and crustaceans have similar playback SNRs relevant to ambient background sound. The frequency-dependent SNR characteristics of this output, including the high-SNR 3-10 kHz components, are similar to those detected during another AE experiment at the Virgin Island sites in Ref. [26], Figure 3, although the Hawaii site

has higher levels of low-frequency reef noise, so the lowest frequency attaining a 5 dB SNR is 500 Hz, instead of 200 Hz at the Virgin Island site. Some ultrasonic output with up to 5 dB SNR occurs between 20 and 25 kHz."

Editor 5: the absence of direct particle motion measurements,

Under future experimental recommendations in the Conclusion, we have added lines 1614-1619:

"Acoustic vector sensors should be deployed for at least one deployment at a new location to confirm that acoustic pressure and particle velocity are proportional to each other, validating acoustic modeling predictions."

Editor 6: and potential spatial or environmental confounds (e.g., transect differences).

Lines 619-634 discuss the confounding effects of transects:

"The CSM settlement results were also influenced by transect, with the XX transect associated with higher settlement. This result may simply be an artifact of how the K,L, and M CSMs next to the speaker were arbitrarily assigned to different transects. A more intriguing possibility is that the XX transect was parallel to a reef wall, and thus may have been more exposed to possible chemical or acoustic natural reef cues than the YY transect. Unfortunately, the spatial extent of the sandy patch used by this particular experimental setup was too small to permit a second transect to be placed in the opposite direction of the XX transect, also parallel to the reef wall. Now that the effective range of the distance effect has been established to be around 17- 20 m, a future experiment may be able to accommodate a different transect design."

Under future experimental recommendations in the Conclusion, we have added lines 1592-1600:

"The biggest single change we would advise is better transect design, to better capture confounding spatial and/or environmental variability. The transects should be arranged so that the speaker is centered inside all structure locations, with at least two transect lines deployed in parallel with a fringing reef, one traveling east from the speaker, and the other west, to ensure equal exposure to potential natural acoustic or chemical cues in the environment."

Please also see our quoted lines in response to Editor comment #2.

We ask that you also address any remaining reviewer comments to the extent possible with the current dataset, including clarifications of interpretation and minor revisions to improve clarity.

Please see responses to specific reviewer comments below.

Responses to individual reviewers

Original reviewer comments are in *italic;* our responses are in plain text, quotes from revised manuscript are in ***bold italic.***

Reviewer #2 (Remarks to the Author):

While the authors have revised the manuscript and clarified several aspects of the study, the major methodological concerns raised in the initial review remain insufficiently resolved. In particular, the issues of pseudo replication and the lack of biologically relevant acoustic characterisation continue to limit the strength of the conclusions that can be drawn from this work, in my opinion.

2.1: Although the authors acknowledge the limitation of conducting a single field experiment, the fundamental issue remains that the acoustic treatment itself was not replicated, which they do not discuss in the manuscript. All settlement structures exposed to acoustic enrichment remain nested within a single sound field generated by one speaker. As a result, settlement structures cannot be considered independent replicates of the acoustic treatment.

While the authors argue that spatial spread of structures reduces this concern, this does not address the core statistical issue that the treatment unit is the playback source, not the individual settlement structures. Therefore, the study still cannot robustly separate acoustic effects from site-specific environmental factors. Coral reef soundscapes are highly heterogeneous at very small spatial scales, and individual soniferous species can dominate local acoustic conditions.

We feel we've given our best response to this in our previous rebuttal. Although structures are exposed to the same acoustic speaker, they receive different levels of exposure, likely experience different levels of background noise and chemical cues, and experience different levels of larval supply. We believe these are more significant sources of environmental and uncontrolled variation than the nature of the acoustic playback itself. We believe that using one speaker with many settlement structures is a more efficient experimental design than many deployments with one speaker and few structures per deployment, in terms of independent samples per unit effort. We agree that additional deployments with replicated speakers in different locations would further strengthen the AE inference and have flagged this as a key priority for future experiments (lines 1570-1585).

"A single AE deployment also cannot address seasonal or genetic variability in potential organism responses; nor can it determine whether acoustic enrichment would work under less benign oceanographic conditions with higher currents and/or wave action. Therefore, additional acoustic enrichment experiments, with a speaker in

different locations, would be required to reduce the possibility of the observed distance effects being the result of a random settlement hotspot, and to demonstrate replication across a wider variety of locations that span different ocean conditions and reef environments. Acoustic recordings from other locations should also be randomly seeded in the replicate deployments to confirm that any attractant effect arises from coral reef ambient sound and not playback artifacts associated with a particular recording."

2.2: For example, territorial damselfish (e.g. Stegastes) can produce intense and frequent acoustic signals and strongly influence local soundscapes around their nests. If such a microhabitat feature was present near the original recording location, the playback may unintentionally reproduce a very specific ecological context rather than a general "healthy reef" soundscape. This is just an example.

This comment states that certain acoustic recordings may be more effective than others in recruiting coral larvae. As we stated in previous rebuttal, our study is more concerned in demonstrating that acoustic enrichment works, and not what particular components or features of the playback are responsible for prompting settlement. We've reviewed the literature again and no study has addressed the question of what components of reef sound are most valid. The acoustic recordings we used are not dominated by fish sounds; they are continuous 24 hour chunks of acoustic data that are dominated by crustacean sounds in terms of time fraction and intensity, with fish calls generally occurring only once or twice a minute. However, we think a more valid objection is that an acoustic enrichment speaker might inadvertently be placed next to a natural reef acoustic hotspot. To that end, see our response to comment 2.1 above. We have also added lines 1553-1558 to the conclusion:

"These explanations include the possibility of a random settlement hotspot that may be generated by natural chemical or acoustic cues released from natural reef structures that happened to coincide with the placement of the acoustic speaker."

2.3 Using a single recording also prevents distinguishing biological effects from potential artefacts introduced by the recording or playback chain (e.g. recorder noise floor, spectral bias, distortion, looping artefacts, or speaker response). Replication of playback tracks is therefore not about optimising which sounds attract larvae, but about ensuring that the observed effect is attributable to acoustic enrichment as a general mechanism rather than to idiosyncratic properties of a single recording. Replication using independent recordings is standard practice in acoustic ecology specifically to avoid confounding treatment effects with recording-specific artefacts.

Please see our response to Comment 2.1. We also note that some recent AE field experiments (e.g. Aoki et al. 2024) have used a consistent set of edited playback files across deployments. In our case, each day's playback consisted of a distinct 24-hour unedited recording from the same site, dominated by crustacean snaps with fish calls occurring only intermittently. Nevertheless, we agree that incorporating independent

recordings from other locations in future deployments would help distinguish general AE effects from recording-specific artefacts. Although we note that if a sound, regardless of whether it is natural reef sound or some artificial acoustic glitch of the equipment, is shown to be associated with increased larval presence, doesn't that still illustrate the basic principle that sound is a statistically significant cue to larvae?

2.4: I also disagree with the authors' interpretation that the broadband nature of reef soundscapes justifies focusing on frequencies above 1 kHz. The biological relevance of acoustic signals should be evaluated based on the sensory capabilities of the receiving organisms rather than the spectral dominance of environmental sound.

Current evidence suggests that coral larvae, like many marine invertebrates, are likely sensitive primarily to low-frequency particle motion rather than high-frequency sound pressure. Therefore, the presence of substantial acoustic energy at higher frequencies (e.g. crustacean snaps) does not necessarily imply biological relevance for coral larvae settlement behaviour.

The reviewer is incorrect in stating that our playbacks were primarily above 1 kHz, but the fault is our own, because our original Figure 8 displayed data on a linear frequency scale, making it difficult to read levels below 1 kHz. We have revised Figure 8 (reproduced below) to display data on a log frequency scale, which now shows significant energy being broadcast between 500 and 1 kHz:

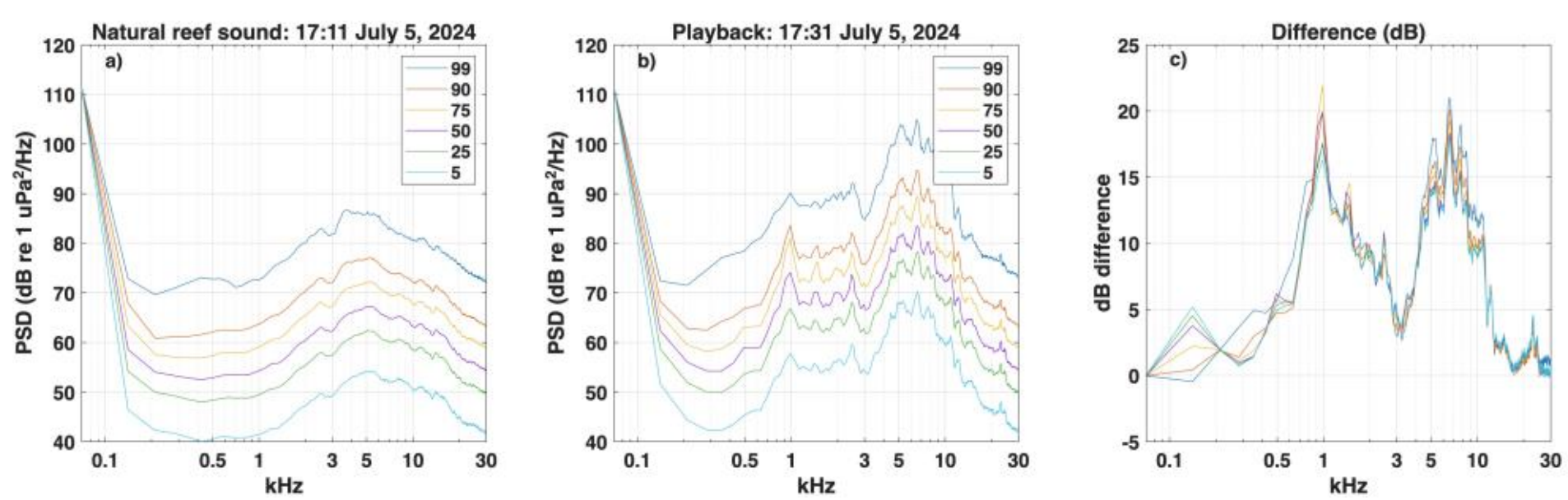

Figure R1: Reproduction of Figure 8 of paper.

We have also revised text in Section 5.2.4 (Playback field measurements) to highlight the similarities between our playback and the only other field AE experiment on coral larvae (lines 978-994):

"Figure 8c shows that the largest signal-to-noise ratios (SNR) of the playbacks, relative to ambient sound, are between 15 and 20 dB, occurring between 500 Hz and 1 kHz and between 5 and 10 kHz. Thus frequency bands associated with both fish and crustaceans have similar playback SNRs relevant to ambient background sound. The frequency-dependent SNR characteristics of this output, including the high-SNR 3-10 kHz components, are similar to those detected during another AE experiment at the Virgin Island sites in Ref. [25], Figure 3, although the Hawaii site has higher levels of low-frequency reef noise, so the lowest frequency attaining a 5

dB SNR is 500 Hz, instead of 200 Hz at the Virgin Island site. Some ultrasonic output with up to 5 dB SNR occurs between 20 and 25 kHz."

For convenience, we reproduce Fig. 3 from Aoki et al (2024) below. Their effective signal to noise ratio (SNR) (red curve in b subtracted from red curve in a) is like the SNR of our playback (Fig. 8c).

We've also added a new section 4.2, which discusses why we feel that coral reef sounds above 1 kHz (even into the ultrasonic range) should be incorporated into AE studies, and monitored.

[figure redacted]

Figure R2: Reproduction of Figure 3 From Aoki et al (2024).

2.5 Furthermore, without measurements of particle motion, it remains unclear whether the biologically relevant components of the acoustic stimulus were properly characterised. As a result, the ecological interpretation of the acoustic treatment remains uncertain. I remain unconvinced by the authors' response regarding the lack of particle motion measurements. While the authors argue that particle velocity is proportional to sound pressure based on modelling, best-practice guidance (see Nedelec et al. 2021) indicates that such assumptions only hold under simplified acoustic conditions such as plane waves or ideal monopole sources. In shallow coastal environments with boundaries (ie typical coral reef) and complex sound fields (underwater speaker is not a monopole), particle motion should ideally be measured directly or estimated from pressure gradient method rather than inferred from a single pressure measurement (note that the authors did not even tried to convert their pressure measurements into particle motion in their revision).

Furthermore, best-practice recommendations emphasise that particle motion measurements are particularly important in biological studies involving fishes and invertebrates, whose hearing is often mediated by particle motion rather than sound pressure. Therefore, relying solely on pressure measurements limits interpretation of the biological relevance of the acoustic stimulus.

Ref: Nedelec, S.L., Ainslie, M.A., Andersson, M.H., Cheong S-H., Halvorsen, M.B., Linné, M., Martin, B., Nöjd, A., Robinson, S., Simpson, S.D., Wang, L. and Ward, J. (2021) Best Practice Guide for Underwater Particle Motion Measurement for Biological Applications. Exeter, UK, University of Exeter for the IOGP Marine Sound and Life Joint Industry Programme, 89pp. & Appendices. DOI: http://dx.doi.org/10.25607/OBP-1726

We have responded to this concern in detail in our previous response, and in Editor comment #5 above.

Under future experimental recommendations in the Conclusion, we have added lines 1614-1619:

"Acoustic vector sensors should be deployed for at least one deployment at a new location to confirm that acoustic pressure and particle velocity are proportional to each other, validating acoustic modeling predictions."

We only add here that our acoustic modeling incorporated the frequency-dependent directional properties of the speaker. We chose the sandy bottom of the experimental site in part to help ensure the propagation characteristics of the field were straightforward to model. We don't disagree that particle velocity is likely a major means that coral larvae detect the world, but we also point that no one knows how coral larvae detect sound, outside of speculation.

Reviewer #3 (Remarks to the Author):

I was a new reviewer invited after the first round of review. Following the editor's request, I have focused on whether the rebuttal adequately addresses the original Reviewer #1 concerns, with particular attention to acoustic monitoring and experimental design. I have also flagged a few technical points the previous reviews did not appear to raise.

3.1 Pseudo replication and single-site design.

The authors' give a good honest response to this and acknowledge the limitations of the data they were able to collect. Reframing the settlement structure as the unit of replication is reasonable given the scale, and the convergence of distance coefficients across the two independently fit regressions (CSM br = -0.08, MCB br = -0.10) provides some indication of robustness.

However, the distance effect is driven primarily by the high settlement counts on structures close (~<6 m) to the speaker. Several structures placed at intermediate distances between 11 and 22 m show only 0-2 larvae each, meaning the data thin out

considerably in the range where the regression is supposed to be resolving the shape of the decline. The exponential slope is therefore driven by the near-speaker high counts on one end and the diffuse low background beyond 21 m on the other, with sparse and noisy data in between. The 21 m influence radius comes from the acoustic propagation model identifying 21 m as the range at which the playback sound merges into background reef noise, and not the settlement data. The settlement data are consistent with that radius, but if the acoustic model had predicted 15 m or 30 m the same settlement data would appear consistent with those values as well because the intermediate-range counts are too sparse to discriminate. For this reason I would ask the authors to add a sensitivity analysis showing how the distance coefficient behaves when the closest (≤2 m) structures are excluded. If the slope holds up without those structures, that demonstrates the gradient is a property of the broader spatial pattern rather than just driven by a handful of high-count points near the source. I think it would strengthen the case for an acoustic enrichment effect substantially. If the slope collapses, the reader and the authors both deserve to know, since it would reframe the finding as "elevated settlement close to the speaker plus a uniform low background farther away" rather than as a smooth, model-supported distance-response curve.

3.1: We appreciate the insight in this comment and have conducted the sensitivity analysis. Please see our detailed comments under Editor comment 1, "Sensitivity analysis of AE effect". The key point is that we find the same distance effect when excluding points less than 4 m range from the speaker, due to the samples recorded on two other more distant CSMs.

3.2: The Conclusion leads with the claim that all three interventions produced "statistically significant effect sizes on coral settlement, both individually and cumulatively.". This puts AE, engineered structures, and Brink coatings on equal footing but in my view the three results don't warrant equal confidence. The Brink effect is supported by a directly paired comparison: at each of several distances, MCB+ and MCB- structures were placed adjacent to one another and the coated structures consistently collected larvae while the uncoated controls collected none. That comparison is internally controlled and doesnt depend on the overall site or acoustic environment. Similarly, the structure-type comparison (CSM vs. MCB+) is supported by both structure types being present at overlapping distances and acoustic conditions, so it too is internally controlled.

The acoustic enrichment result is different. It rests on a single speaker at a single location during a single spawning event, and the inference that settlement responds to AE comes from a spatial gradient relative to that one source. Within-experiment statistical significance is real and defensible, but I'm not confident that generalizable significance has been established for the AE component specifically. I recommend softening that opening sentence with respect to AE alone, while leaving the Brink and structure-type findings to carry the stronger framing they have earned. A version that distinguishes between "robustly demonstrated" (Brink, structure type) and "suggestive and warranting follow-up" (AE) would more accurately match the strength of the underlying evidence.

3.2: This is a reasonable suggestion, and please see our detailed response to Editor Comment #2 above.

3.3: Fish herbivory and fish-larvae chemical cues. The fish-camera analysis goes some way to ruling out the mature-fish herbivory mechanism. The fish-larvae signal (4-10x more larvae at the treatment site across three deployments in the companion paper) is an interesting finding in its own right. It raises a speculative question about whether fish-larvae chemical cues could contribute to the coral settlement pattern. This is outside my area of expertise and I defer to the authors on biological plausibility, but as someone outside the direct subfield the claim that the fish-larvae signal is too small or brief to have any influence on coral settlement is surprising on its face. I would suggest a sentence in the Discussion noting that an indirect pathway via fish-larvae chemistry cannot be definitively ruled out without water-chemistry sampling, while acknowledging that no specific mechanism for such a pathway appears to be well-established in the coral settlement literature (from a brief look). That would close the loop cleanly without overstating the concern.

3.3: We have added a paragraph in the Discussion (lines 694-708) that addresses this point:

"The lack of observed difference in mature reef fish presence at the treatment and control site would seems to provide evidence against this alternate hypothesis. However, we cannot definitely rule out the possibility that increased fish larvae presence at the AE site might indirectly affect coral settlement, either through increased herbivory or even the generation of potential chemical cues by the fish larvae, that could then attract coral larvae. No specific chemical mechanism between fish presence and coral larvae response seems to exist in the literature, but future experiments in this vein might consider collecting water samples to test for possible differences in chemical cue levels."

3.4: Particle motion vs. pressure. Reviewer #2's concern was that coral larvae detect particle motion rather than pressure. The authors' response is that in the far field of a source in open water, pressure and particle motion are proportional so a pressure-based 21 m influence range translates directly to a particle-motion range. This holds cleanly in the frequency band where the playback actually carries energy above background (1-15 kHz), where wavelengths are 10-150 cm and are small relative to the 5 m water depth. The proportionality would break down at lower frequencies (e.g., 500 Hz, where ~3 m wavelength makes the whole water column "near a boundary"), but the playback carries little energy below 1 kHz, so this regime is not directly relevant to the experiment. The authors also cite Jones et al. (2025) as empirical support and explicitly flag the absence of direct particle-motion measurement as a limitation. I find this adequately addresses the concern. Reviewer #2 may prefer direct measurement in future work, but for this study the pressure-based characterization is well-matched to the playback content.

No changes made in response to these comments. See also our response to comment 2.5 from Reviewer #2.

3.5: Frequency range. This appears adequately resolved, though I don't have the expertise or time to assess this fully.

Please see our response to Comment 2.4.

3.6 Paper organization. There have been a number of changes to improve readability: acronym list, consistent nomenclature, consolidated Figure 1, and Appendix A. I found the manuscript readable.

No changes made.

3.7: Additional points:
MCB+ vs. MCB- coefficient interpretation. Every MCB- has zero settlement at every distance. The Brink coefficient is $e^{7.04} \approx 1100$ with a 95% CI of [1.97, 21.85] in log units. This is a saturation artifact of regressing against an all-zero comparison group. The multiplicative interpretation is essentially meaningless. I think it's defensible to use the posteriors to claim a directional effect, but wanted to flag that it's hard to interpret the scale.

The revised paragraph in question now reads as follows (lines 409-412):

"The exact magnitude of the coefficient has little meaning, given that no settlement was detected on the MCB- structure, but the implied scale of the results (i.e. a factor of e^{7.04}=1100 increase in density) indicates the strength of the BRINK effect."

3.8: Transect XX vs. YY systematic difference. The regression finds settlement on Transect XX is ~2.5 times higher than on YY. The authors use a categorical TRANSECT term that shifts the curve up or down without changing its slope. The authors treat this as a replicate effect, but the two transects aren't really equivalent replicates. Transect XX runs parallel to the reef, so all CSMs along it sit at roughly the same (relatively close) distance from natural coral. Transect YY points away from the reef into open sandy bottom, so CSMs along YY must get progressively farther from any natural reef as they get farther from the speaker. Proximity to the reef is itself a candidate covariate for larval supply and for chemical or acoustic cues from natural coral, and the two transects differ systematically on that.
This is a plausible mechanistic explanation for the intercept difference the TRANSECT term currently absorbs: XX gets a ~constant boost from reef proximity, YY doesn't. But if reef proximity is taken seriously, it may also bias the two slopes in opposite directions. Along XX, reef proximity is roughly constant with distance from the speaker, so the XX slope should reflect AE relatively cleanly. Along YY, reef proximity declines with distance from the speaker (the two are correlated), so the YY slope mixes a declining AE effect

with a declining reef-proximity effect and may overstate the AE distance dependence. I don't think this damages the findings, but wanted to highlight it and recommend the authors mention this as a potential confound and consider this for future work.

We appreciate the insights of this comment. The following paragraph has been added to the Discussion (lines 619-634).

"The CSM settlement results also seemed influenced by the transect, with the XX transect associated with higher settlement. This result may simply be an artifact of how the K, L, and M CSMs next to the speaker were arbitrarily assigned to different transects. A more intriguing possibility is that the XX transect was parallel to a reef wall and thus may have been more exposed to possible chemical or acoustic natural reef cues than the YY transect. Unfortunately, the spatial extent of the sandy patch used by this particular experimental setup was too small to permit a second transect to be placed in the opposite direction of the XX transect, also parallel to the reef wall. Now that the effective range of the distance effect has been established to be around 17- 20 m, a future experiment may be able to accommodate a different transect design."

3.9: Minor
~L568 "provided permitted" - check you intended both words.
~L626 "arose due from."
A final pass would catch these and a handful of similar issues.

Minor grammatical mistakes have been reviewed and corrected, and another full read of the manuscript has been conducted to catch minor typos.

On behalf of all co-authors, I sincerely appreciate the feedback provided thus far from reviewers and the Editor.

Sincerely,
Aaron Thode and Co-Authors
